# Single-cell RNA sequencing coupled to TCR profiling of large granular lymphocyte leukemia T cells

**DOI:** 10.1038/s41467-022-29175-x

**Published:** 2022-04-11

**Authors:** Shouguo Gao, Zhijie Wu, Bradley Arnold, Carrie Diamond, Sai Batchu, Valentina Giudice, Lemlem Alemu, Diego Quinones Raffo, Xingmin Feng, Sachiko Kajigaya, John Barrett, Sawa Ito, Neal S. Young

**Affiliations:** 1 Hematology Branch, National Heart, Lung, and Blood Institute, National Institutes of Health, Bethesda, MD USA; 2 Division of Hematology-Oncology, Department of Medicine, University of Pittsburgh, Pittsburgh, PA USA

**Keywords:** Transcriptomics, Next-generation sequencing, T cells, Cancer, Haematological diseases

## Abstract

T-cell large granular lymphocyte leukemia (T-LGLL) is a lymphoproliferative disease and bone marrow failure syndrome which responds to immunosuppressive therapies. We show single-cell TCR coupled with RNA sequencing of CD3^+^ T cells from 13 patients, sampled before and after alemtuzumab treatments. Effector memory T cells and loss of T cell receptor (TCR) repertoire diversity are prevalent in T-LGLL. Shared TCRA and TCRB clonotypes are absent. Deregulation of cell survival and apoptosis gene programs, and marked downregulation of apoptosis genes in CD8^+^ clones, are prominent features of T-LGLL cells. Apoptosis genes are upregulated after alemtuzumab treatment, especially in responders than non-responders; baseline expression levels of apoptosis genes are predictive of hematologic response. Alemtuzumab does not attenuate TCR clonality, and TCR diversity is further skewed after treatment. Inferences made from analysis of single cell data inform understanding of the pathophysiologic mechanisms of clonal expansion and persistence in T-LGLL.

## Abstract (web summary)

T cell large granular lymphocyte leukemia (T-LGLL) and the cellular phenotype underlying response to therapy is not well understood. Here the authors use single cell sequencing to better understand changes in T cell clonal frequency and gene expression before and after therapy in T-LGLL.

## Introduction

T-cell large granular lymphocyte leukemia (T-LGLL) is a lymphoproliferative disease usually presenting with cytopenia, and typically characterized by clonal expansion of terminally differentiated effector-memory cytotoxic T lymphocytes (CTL). T-LGLL has been hypothesized to be driven by chronic antigen exposure, resistance to Fas-FasL-mediated apoptosis, and constitutive activation of signaling proteins in the Janus-kinase (JAK) and other survival pathways^1–3^. JAK-STAT pathway activation is present in almost all cases, and gain-of-function mutations in *STAT3* and *STAT5B* have been identified in about half of patients^3^. Sequencing of the antigen-binding region (complementarity-determining region 3, CDR3) and measurement of the variable β chain (Vβ) of the T cell receptor (TCR) by flow cytometry provides clinical evidence of T cell clonal expansion in T-LGLL^4,5^. Vβ flow cytometry and TCR**γ**-polymerase chain reaction (PCR) analysis are well correlated, but current panels of monoclonal antibodies only include about 75% of the Vβ spectrum^4^. Application of next-generation sequencing (NGS) to both Vβ and Vα chains has not been routine, and paired chain information is lost in the sequencing of bulk cell populations, which can only be paired using statistical algorithms^6,7^. Therefore, high-resolution profiling of the TCR repertoire in T-LGLL is lacking. Efforts to characterize the transcriptome of clonal CTL using whole peripheral blood or after enrichment of specific clones by flow cytometry have not provided satisfying resolution and scale, due to heterogeneity among patients.

T-LGLL is treated successfully with a variety of immunosuppressive drugs, mainly cyclosporine, and lymphocytotoxic agents, such as methotrexate; inhibition of JAK-STAT signaling also appeared effective in a pilot trial^1^. While therapies directed at the T-LGLL cell populations are efficacious, the basis for response and lack of response in some cases is unknown. Peculiarly, improvement in blood counts can occur without eradication of the CTL clones, which frequently persist after discontinuation of treatment, as in our recent study of alemtuzumab, a monoclonal anti-lymphocyte antibody, in refractory T-LGLL^1^.

Single-cell TCR V(D)J sequencing coupled with RNA sequencing enables profiling of paired TCRα and TCRβ chains at single-cell resolution at high-throughput^7,8^, as well as coupled global gene expression in the same cell, making it possible to characterize T cell clonal expansion in steady state and in disease, and to track transcriptome changes of the same clone over the course of the disease and with treatment^7,9–13^. Studies employing single-cell RNA sequencing (scRNA-seq) analysis have helped to define immune cell subsets in tumors and in checkpoint inhibitor treatment^14–17^, and in autoimmune diseases^18^, but no such studies have been performed in T-LGLL.

We applied single-cell TCR sequencing (scTCR-seq) coupled with scRNA-seq to CD3^+^ T cells obtained from a relatively large cohort of T-LGLL patients, who have been well characterized clinically and by conventional laboratory testing as part of a clinical protocol to a prospectively test therapy with alemtuzumab in refractory disease. We sought to characterize the TCR repertoire and to define pathophysiologic mechanisms at the single-cell level. Further, we wished to determine mechanisms of action of an immune therapy in this disease. Our approach should be applicable to other syndromes characterized by a T cell pathophysiology but more subtle clonal expansion.

## Results

### scRNA-seq of T cells in T-LGLL patients demonstrates expansion of CD8^+^ effector T cells

**Fig. 1:**
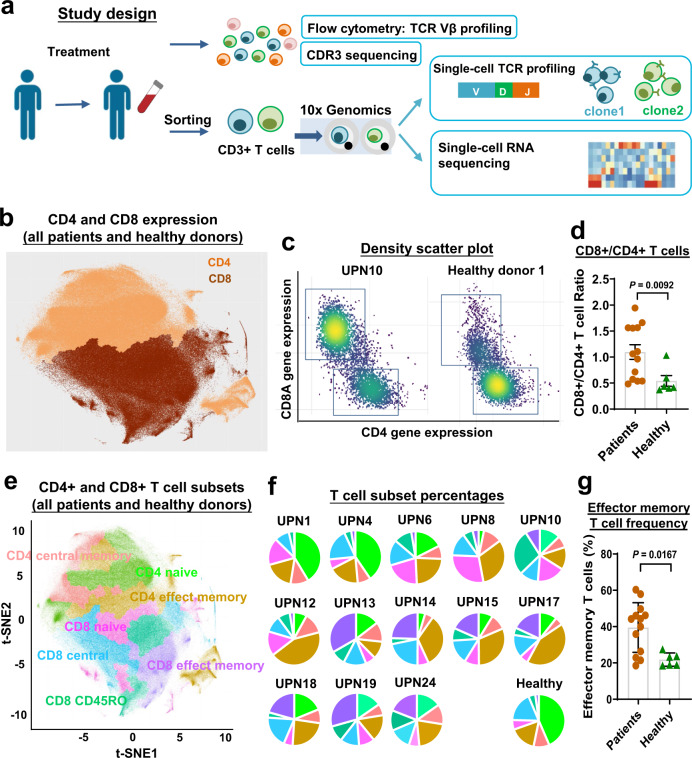
A T cell landscape in T-LGLL patients. **a** A scheme of experimental study design. **b** A t-distributed Stochastic Neighbor Embedding (t-SNE) plot of single-cell gene expression of T cells in all patients and healthy donors, colored by CD4^+^ and CD8^+^ T cell clusters. **c** Density scatter plots of CD4 and CD8A expression in T cells, generated using scRNA-seq results. *x*-axis, CD4 gene expression; *y*-axis, CD8A gene expression; dots were colored by cell density. **d** A CD8^+^/CD4^+^ T cell ratio was compared between patients (*n* = 13) and healthy donors (*n* = 6). Data are presented as mean values ± SEM; two-sided unpaired Mann–Whitney test. *P* value = 0.0092. **e** The same t-SNE plot in (**b**), color coded for CD4^+^ and CD8^+^ T cell subsets. **f** Pie charts showing percentages of T cell subsets in individual patients and healthy donors; color scheme as in (**e**). **g** Percentages of effector memory T cells were compared to patients (*n* = 13) and healthy donors (*n* = 6). Data are presented as mean values ±  SEM; two-sided unpaired Mann–Whitney test, *P* value = 0.0167.

To depict a landscape of phenotype, functional state, and clonality of T cells in T-LGLL patients, we constructed an atlas comprising ~500,000 CD3^+^ T cells collected from 13 patients (M/F, 7/6; median age 51 years, range 26–85) whose blood samples were obtained before and after a 3–6 months course of alemtuzumab, and from blood donated by seven matched healthy controls (Supplementary Table 1). Our study is schematized in Fig. 1a. Sequencing metrics are shown in Supplementary Data 1.

To enable systematic comparison across patients' pre- and post-treatment, we merged data from all individuals and different time points^19^ (Supplementary Methods and Results). Batch effects were corrected by the sva package^20^, as demonstrated by a high entropy-based measure for quantifying mixing of samples. We further verified cell annotation and sample mixing by expression of signature genes in CD4^+^ and CD8^+^ T cells^21^ (Fig. 1b and Supplementary Fig. 1). CD4^+^ and CD8^+^ T cells were expected to constitute the majority of enriched CD3^+^ T cell populations, and they clustered distinctively. We observed a large degree of variation in CD4/CD8 compositions among individuals: CD8^+^ subsets ranged 32.6–87.5% in patients prior to alemtuzumab treatment, and there was a generally elevated CD8^+^ subset in T-LGLL patients (Fig. 1c, d). We then verified that major T cell subsets were identifiable in each patient using PhenoGraph clustering^11^. We annotated clusters by genome-wide correlations between cluster mean expression and previously characterized transcriptional profiles of sorted bulk datasets (GSE93777 in GEO) ^22^, and identified naive, central memory, and effector memory T cell clusters (Fig. 1e). Annotations were confirmed and refined using the expression of canonical markers (Supplementary Fig. 2). There was variable composition of subclusters among patients, with effector memory T cells more prevalent in patients (Fig. 1f, g), in agreement with the known expansion of phenotypical effector memory T cells^23^.

### Loss of TCR repertoire diversity in T-LGLL

TCR clonality has been historically measured using TCRVβ monoclonal antibodies in flow cytometry and CDR3 sequencing^4,24^. We first studied resolution of scTCR-seq in identifying clonality in T-LGLL. Among all patient samples, we detected at least one productive α-chain in 27–89% (median 61%), and at least one productive β-chain in 79–98% (median 95%) of cells, of which 27–80% (median 58%) of cells had only one productive α-chain, and 60–95% (median 87%) had only one productive β-chain. There were 6–73% (median 45%) cells with paired productive αβ chains, and some cells with multiple TCRα- and/or β-chains (Supplementary Table 2). These data are similar to previous single-cell results based on TCR sequence^7^. α chains are less likely to be detected by all current approaches of TCR sequencing^7^, partially due to lower expression of *TRAV* genes than of *TRBV* genes (Supplementary Fig. 3a).

**Fig. 2:**
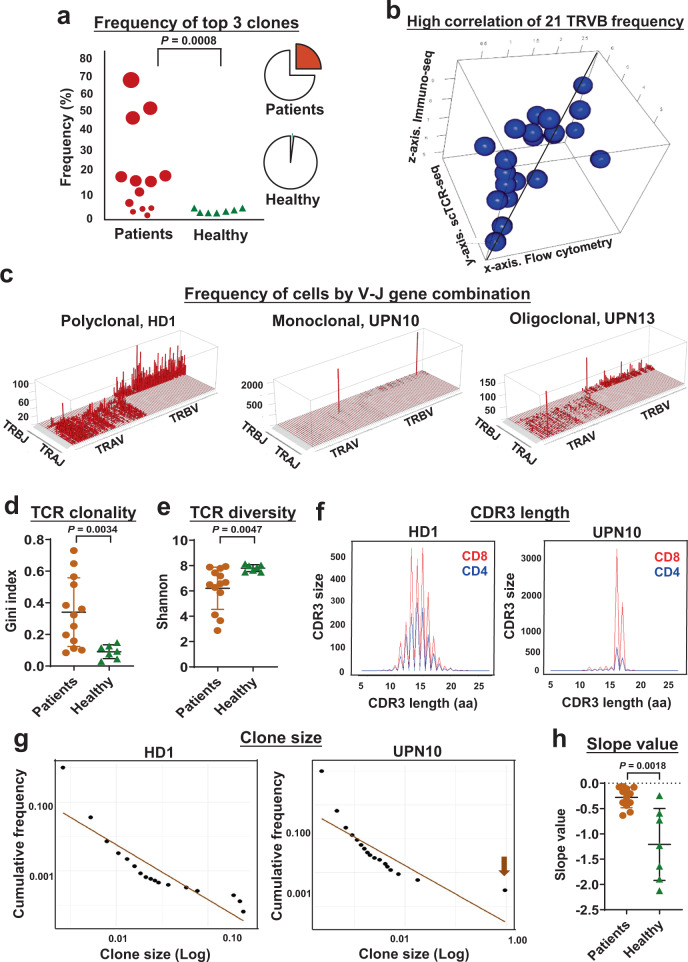
Loss of TCR repertoire diversity in T-LGLL patients. **a** Dot plot showing frequency of a sum of top 3 clones in individual patients and healthy donors. Dot sizes are proportional to frequency of top 3 clones. Pie charts on the right show medium percentages of a sum of the top 3 clones (red parts) in patients (top) and healthy donors (bottom). Two-sided unpaired *t*-test. *P* value =  0.0008. **b** Three-dimensional dot plot showing high correlation of frequency of 21 TRVB detected by flow cytometry (*x*-axis), scTCR-seq (*y*-axis) and immuno-seq (*z*-axis) in individual patients with an average correlation coefficient of 0.503. **c** Skyscraper plots showing Vβ/Vα and matching Jβ/Jα in healthy donor 1 (HD1) and representative patients (UPN10 and UPN13). Gini index (**d**) and Shannon index (**e**) of TCR clonality were compared in patients (*n* = 13) and healthy donors (*n* = 7). Data are presented as mean values ±  SEM; two-sided unpaired Mann–Whitney test. *P* value = 0.0167 (**d**) and *P* value = 0.0047 (**e**). **f** CDR3 lengths of representative HD1 and patient UPN10 were plotted, with CDR3 lengths in amino acid (aa) on the *x*-axis and frequency (CDR3 size) on the *y*-axis (overlapped curves showing CD8^+^ and CD4^+^ T cells in red and blue, respectively). **g** Clone sizes were plotted in HD1 and UPN10, with log clone sizes on the *x*-axis and log cumulative frequency on the *y*-axis. **h** Slope values of power-law fitting plots were compared in all patients (*n* = 13) and healthy donors (*n* = 7); plots of all individuals are shown in Supplementary Figs. 6 and 7. Data are presented as mean values ± SEM; two-sided unpaired Mann–Whitney test. *P* value = 0.0018.

Here, we define “expanded clones” as ≥10 T cells with identical TCRα- and β-chains^25^. The top three expanded TCR clones comprised up to 70% of a sequenced CD3^+^ T cell population in patients, but there was variablity among cases (Fig. 2a). scTCR-seq in the current study showed agreement with results in the same cases from flow cytometry and CDR3 sequencing in defining top clones, but provided much higher resolution (Fig. 2b). There was a positive correlation (*R* = 0.503) between reads for each Vβ/Vα in TCR sequencing, matching Jβ/Jα (defined in www.imgt.org) and values with flow cytometry. By plotting the number of cells of each V and J gene match, patients showed clonal expansion (Fig. 2c and Supplementary Figs. 4 and 5)^4^. Unlike the multiclonal T cell repertoire of healthy donors, plots of patients’ results showed dominance of one or a few specific CDR3 sequences, indicative of the expected clonal expansion of CTLs in T-LGLL. The Gini index measures equality of distribution^26,27^; for TCR diversity, a Gini index ranges between zero to one, and is positively correlated with T cell clonality. There was a significantly higher Gini index of TCR in patients’ samples (0.340 ± 0.217) compared with the index in healthy donors (Fig. 2d; 0.091 ± 0.043, *P* = 0.014, *t*-test). We also calculated a Shannon’s entropy (H) index to evaluate the diversity of the TCR repertoire of each sample^27^. The H index, which positively correlates with T cell diversity, was significantly lower in patients than in healthy donors (Fig. 2e). These results indicated markedly less diversity of the TCR repertoire in T-LGLL. Loss of TCR repertoire diversity was also demonstrated by the abnormal size distribution of CDR3 and a larger scale power-law distribution of clone sizes (Fig. 2f–h and Supplementary Figs. 6–9).

### Absence of common TCRA and TCRB clonotypes in T-LGLL patients

**Fig. 3:**
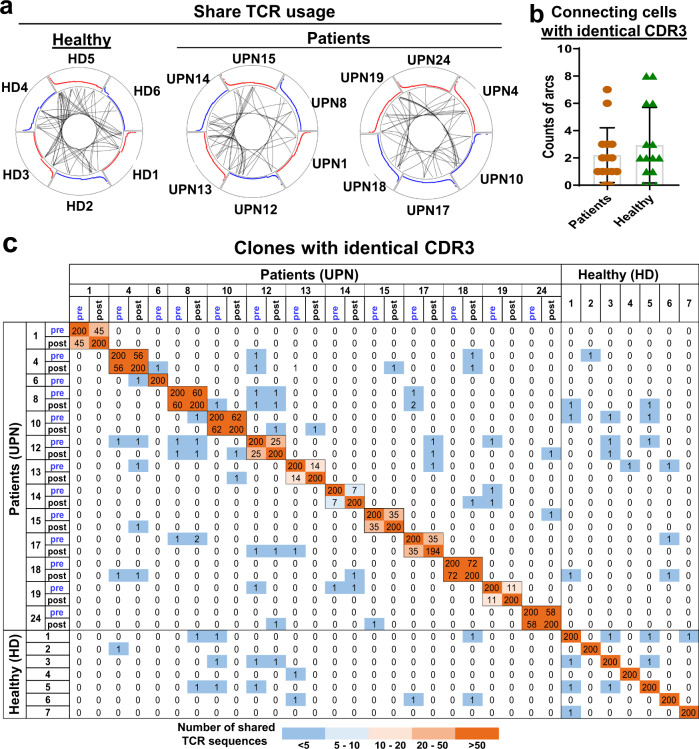
Lack of common TCRA and TCRB clonotypes in T-LGLL patients. **a** Circos plots are shown: segments in circles represent individual cells yielding rearranged TCR sequences. Black lines connected clones sharing identical CDR3 sequences among individuals. Sharing of identical CDR3 sequences among six healthy donors (HD1–HD6) is shown on the left; sharing of identical CDR3 sequences among patients (UPNs 1, 8, 12, 13, 14, and 15) and patients (UPNs 4, 10, 17, 18, 19 and 24) on the right. Red and blue curves are proportional to clone sizes. **b** Counts of arcs connecting cells with identical CDR3 sequences among patients (*n* = 13) and healthy donors (*n* = 7) were compared pair-wisely, and there was no significant difference. Data are presented as mean values ± SEM; two-sided unpaired Mann–Whitney test. **c** Heatmap plot showing shared CDR3 sequences among the top 200 TCR clones of patients and healthy donors. On both *x*- and *y*-axes, samples of patients and healthy donors are listed, and adjacent grids show paired samples (pre- and post-treatments) of the same patients. Counts of identical TCR clones shared among samples are shown. Color scheme, ranging from dark orange to dark blue, indicates the number of shared TCR CDR3 sequences, from high to low.

Clonal T-LGLL expansion has been hypothesized to derive from chronic antigenic stimulation, and we examined for evidence of CDR3 homology that might implicate a potential common antigen. We used the circlize package (https://cran.r-project.org/web/packages/circlize) to visualize shared TCR usage among subjects^24,28^, and observed that junctions in circuit plots were shared between subjects at low levels, similar to healthy donors (Fig. 3a, b; *P* = 0.41, *t*-test). Two samples shared TCR clones with an identical nucleotide CDR3 sequence, shown as arcs within circus plots. Dominant clones in patients were expressed at low levels in a few other patients, but they were also present in healthy donors (including for top 200, top 500, and top 1000 clones among individuals; Fig. 3c and Supplementary Figs. 10 and 11). In comparison with three independent datasets^6,24,29^, TCR usage overlapped with dominant sequences in our patients and were also present in healthy donors; dominant TCR clones were shared only at low levels among 20 T-LGLL patients who have been recently reported (Supplementary Figs. 12 and 13 and Supplementary Data 2). Collectively, these results imply CD8^+^ T cell clonal expansion to be subject-specific, or “private” to individual patients, and TCR usage as not disease-specific. Such a lack of common TCR clonotypes among T-LGLL patients has been previously observed^5^.

### Homologous T-LGLL specific CDR3 and effects on transcriptional phenotypes

**Fig. 4:**
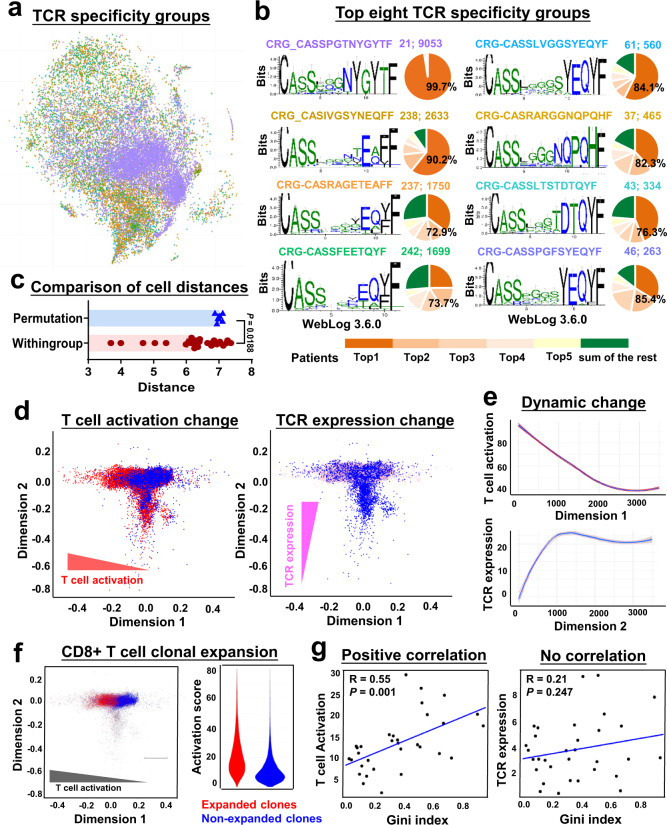
TCR usage and activation stages shape T cell phenotypes. **a** The same t-SNE plot in Fig. 1b with single T cells from all patients and healthy donors, colored by TCR specificity groups identified by GLIPH. **b** Sequences and corresponding weblogs of top eight TCR specificity groups with more than five different CDRs. In each panel, pie chart on the right indicates percentages for this TCR specificity group in top 5 patients (number in a black color) and in all other patients. In numbers A; B following CRG sequences, A indicates the number of clones contained in the CRG, B indicates the frequency of this CRG in all cells. **c** Distances of cells within the same TCR specificity group were compared to permutated distances of any random cells in the t-SNE plot; two-sided unpaired Mann–Whitney test. *P* value = 0.0188. **d** Diffusion maps to visualize components of CD8^+^ T cell phenotype variation from all patients and healthy donors. Each dot represents a CD8^+^ T cell. T cell activation, TCR expression, and all other cells are colored in red, pink, and blue, respectively, with dimension 1 on *x*-axis and dimension 2 on *y*-axis. Diffusion maps showing dynamic changes of T cell activation on dimension 1 (left, in red) and TCR expression on dimension 2 (right, in pink). **e** Curves indicate dynamic changes of T cell activation and TCR expression along on dimension 1 and dimension 2 revealed on diffusion maps, respectively. *x*-axis, dimensions on diffusion maps; *y*-axis, imputed expression of T cell activation (top, in red) and TCR expression (bottom, in pink) components. Solid lines in red and pink represent 5–95% interval, illustrated by shaded areas; blue line shows a medium. **f** Diffusion map of CD8^+^ T cells on dimension 1 and dimension 2, and colored by clonal expansion (red for expanded clones). Higher activation is observed in expanded clones than in non-expanded clones (a violin plot on the right). **g** Gini index positively correlated with T cell activation (left) but not with TCR expression (right). Each dot indicates one sample. A Pearson correlation test.

Due to the lack of common clonotypes among T-LGLL patients, we grouped enriched CDRs in order to identify clustered groups of TCRs present in many different samples, which might be selected for binding to a potential common antigen. For more comprehensive evaluation of CDR3 motifs, detailed in silico analysis was performed on top 500 combined TCRB CDR3 sequences. We used GLIPH (grouping of lymphocyte interactions by paratope hotspots) to identify ‘TCR specificity groups’: clusters of distinct TCR sequences that are likely to recognize common antigens via shared motifs in CDR3 sequences^30^. We identified 49 TCR specificity groups with more than five different CDR sequences (Fig. 4b, Supplementary Figs. 14 and 15 and Supplementary Table 3). Top TCR specificity groups occupied a confined region in a t-SNE projection (Fig. 4a). Analagous to T cells of a given clonotype (sharing identical TCR sequences) having a similar transcriptional phenotype, T cells expressing distinct TCRs but within a TCR specificity group tended to have more transcriptome similarity (a shorter distance in a t-SNE plot) compared to those with randomly grouped TCRs (Fig. 4c; *P* < 0.0002, unpaired *t*-test). The pairwise distances of cells in a t-SNE plot (based on transcriptome) positively correlated with TCR sequence dissimilarity (Damerau-Levenshtein distance; Supplementary Fig. 16a). These results suggested clonally expanded T cells highly correlated to transcriptional phenotypes.

We sought convergence groups (CRGs) that were shared by subjects and thus more likely to reflect selection by potential common antigens. Within individual CRGs, we graphed percentages of cells from top five patients (only among samples obtained prior to treatment) whose TCRs comprised the majority of the CRG. Top eight CRGs with highest cell numbers were enriched in effector memory and CD45RO^+^CD8^+^ clusters (Fig. 4a and Supplementary Figs. 16b, c and 17). A sequence CRG_CASSPGTNYGYTF was dominant in cells from UPN15; there were 73% of cells in CRG-CASRAGETEAFF that were contributed by these five patients, and they displayed high usage of TRBV5-6 and TRBJ2-2; in the remaining top CRGs, cells from the five patients contributed the most cell numbers constituted 74–90% of individual CRGs. From these findings, we inferred that CD8^+^ T cell expansion in T-LGLL might be driven by similar antigens across some but not most patients^31^. To understand common transcriptional signatures of clonally expanded T cells in each CRGs, we compared gene expression of T cells in a specific CRG with that of all other cells, and plotted top CRG-specific genes of those 49 TCR specificity groups (Supplementary Fig. 18a and Supplementary Data 3), followed by Gene Ontology (GO) term enrichment of differentially expressed genes in each CRGs. T cell activation and immune response genes, and cell cycle genes, were upregulated in the majority of CRGs. We inferred that expanded clones were activated, and may have acquired growth, survival and functional advantages due to upregulation of cell cycling and immune response genes (Supplementary Fig. 18b and Supplementary data 4).

Among top CRGs, top four CRGs enriched in patients rather than in healthy donors were CRG-CASSPGTNYGYTF, CRG-CASIVGSYNEQFF, CRG-CASRAGETEAFF and CRG-CASSLVGGSYEQYF (Supplementary Fig. 19a). Some CDR3s in CRG-CASIVGSYNEQFF and CRG-CASRAGETEAFF groups were present in clones with sizes larger than ten in 10X donor 3 (included in 10x Genomics sample VDJdb datasets and is CMV seropositive)^32^. These CRGs were overrepresented in T-LGLL patients who were seropositive for CMV than among others in our cohort. A recent study^30^ has identified five CMVpp65 amino acid motifs (TGT, ATN, FQ, SSA and QTG) of CDR3s in CMV-seropositive individuals with HLA*0201. In our samples, these motifs of HLA-A*0201, CMV^+^ CDR3s were more represented in expanded clones of CMV^+^ subjects than in CMV^−^ cases (Fisher test; *P* = 0.033). These results added to validity of our current analysis and to a hypothesis that chronic CMV antigen stimulation is a potential driver of T cell clonal expansion^33^.

We input β-chain CDR3 sequences of T-LGLL patients into TCRmatch^34,35^ in order to identify epitopes and related antigens. Within the expanded TCR sequences from T-LGLL patients, there were four epitopes derived from common viral pathogens: EBV, CMV and influenza A virus (Supplementary Fig. 19b and Supplementary Data 5-1). We specifically examined for putative antigens of dominant clones (top 10 clones) in T-LGLL patients using TCRmatch (Supplementary Data 5-2). However, among these very top clones, the majority of TCR sequences could not be mapped to any epitopes of any antigen. Further, the majority of top clones in patients with monoclonal expansion could not be mapped to any epitopes in TCRmatch. However, our analysis was not comprehensive, as inevitably limited by the small number of subjects and epitopes in reference databases, compared with the potential huge number of TCRs. In addition, matching our CDR3 sequences with virus-specific CDR3 sequences in a second database VDJdb^32^ showed that most CDR3s derived from CMV and other common viruses. Good correlation of CDR3 compositions (grouped by viruses) in patients and in healthy donors indicated that clonal expansion could not be explained by exposure to common viruses (Supplementary Fig. 20 and detailed in Supplementary Results).

### TCR usage and activation states contribute to T cell phenotypes

Our data (Fig. 4a) indicated that, at least in part, TCR utilization impacted T cell phenotypes. Using integrated data from all individuals and at all time points, we used diffusion maps^11,12^ to visualize T cell phenotype variation, and to highlight expression of components related to T cell activation and T cell terminal differentiation, in order to examine their contribution to T cell phenotypes using regression analysis^11^. We observed T cell activation to be the most informative contributing component on dimension 1, and there was a continuous pattern of altered T cell activation (Fig. 4d, e). Other components, including T cell terminal differentiation, proinflammatory, and cytolytic effector pathways, contributed in the same direction as did T cell activation on dimension 1, perhaps due to a moderate overlap of genes in each pathway but also likely from coordination of genes of related functions (Supplementary Fig. 21a–d). When denoted by colors with T cell subpopulation signature genes in CD8^+^ T cells, the differentiation trajectory appeared from naive T cells to central and effector memory T cells (Supplementary Fig. 21e, f). By correlation analysis, 22% of variation across T cell phenotypes was attributed to TCR expression, and TCR expression showed a continuous increase on dimension 2 in the diffusion map (Fig. 4e)^8,12^. Together, these results suggested that T cell phenotypes were contributed by a combination of antigenic TCR stimulation and environmental stimulation. On the same diffusion map with color coding of expanded and nonexpanded clones, expanded clones clustered at the left side of dimension 1, indicating an impact of clonal expansion on the transcriptome in T-LGLL (Fig. 4f). In patients and healthy donors, there was positive correlation between TCR diversity and T cell activation, but not with TCR expression (Fig. 4g). In summary, CTL expansion in T-LGLL associated with decreased TCR diversity and increased T cell activation.

### Deregulation of cell survival and apoptosis gene programs in T-LGLL

**Fig. 5:**
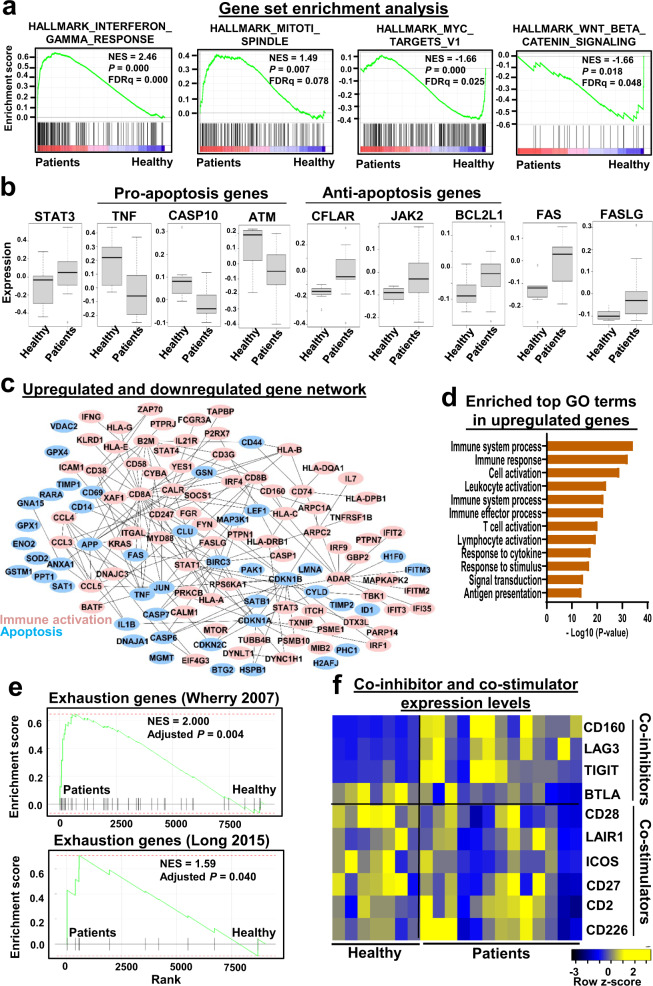
Dysregulated gene programs in T-LGLL. **a** Gene Set Enrichment Analysis (GSEA) plots of differentially expressed genes in T-LGLL patients compared with those in healthy donors. GESA based on a Kolmogorov Smirnov test. **b** Boxplots showing expression of pro-apoptosis and anti-apoptosis genes in T-LGLL patients (*n* = 13) compared with those in healthy donors (*n* = 7). Shown are 25–75% response ranges (top and bottom lines of boxes) and minima and maxima (bars). A the two-sided unpaired *t*-test. **c** A network of upregulated genes involved in the immune response and cell survival, and downregulated genes involved in apoptosis in T-LGLL patients. **d** Bar chart showing top GO terms enriched in upregulated genes in expanded clones compared to those in non-expanded clones in T-LGLL, Fisher’s exact test. **e** GSEA plots of T cell exhaustion genes in T-LGLL patients compared with those in healthy donors. T cells in patients consistently expressed higher levels of exhaustion markers, gene lists were from two previous publications^42,43^. GESA is based on a Kolmogorov Smirnov test. **f** T cells in patients did not consistently express higher levels of co-stimulators or lower levels of co-inhibitors.

To understand deregulated gene programs in T-LGLL, we first compared gene expression in patients prior to alemtuzumab and healthy donors, and utilized gene set enrichment analysis to explore global changes in gene programs in T-LGLL^3,36,37^. Upregulated genes in patients were highly enriched in the immune response and signaling pathways of cell survival (hallmark gene sets including interferon response, PI3K_AKT_MTOR and IL6_JAK_STAT3 signaling) and cell proliferation (mitotic spindle), while genes less expressed in T-LGLL were enriched in the apoptosis pathways (wnt beta_catenin signaling, MYC_targets, and apoptosis) (Fig. 5a and Supplementary Data 6). Downregulation in apoptosis gene sets was particularly marked in CD8^+^ T cells. These results, consistent with earlier publications, indicated imbalance of immune activation, cell survival and cell apoptosis gene programs in T-LGLL^2,3^. We then utilized Gene Ontology^38^ to study enriched functional terms of top differentially expressed genes in T-LGLL. Upregulated genes were highly enriched in immune response and cell activation (Supplementary Figs. 22 and 23). Our group previously measured expression of 84 genes in the JAK-STAT pathway by RT-qPCR in patients included but not limited to the current patient cohort, and we again observed activation of these genes in T-LGLL patients; results from scRNA-seq and qPCR showed good correlation (*R* = 0.31, *P* < 0.0001; Supplementary Fig. 24). Cell apoptosis genes including *TNF*, *CASP10*, and *ATM* were downregulated (GO:0006915 apoptotic process, GO:0008219 cell death and GO:0012501 programmed cell death), anti-apoptosis genes (*CFLAR*, *JAK2*, and *BCL2L1*) were upregulated, although *Fas* and *FasL* were overexpressed in patients (Fig. 5b). These results support previously proposed mechanisms of dysregulation of apoptosis and constitutive activation of survival pathways in T-LGLL^3,23^. We mapped top differentially expressed genes to a protein-protein interaction network using STRING data^39^, followed by jActiveModulesTopo^40,41^ to identify biologically meaningful gene subnetworks (Fig. 5c). In this subnetwork, CD8A was the most distinct hub gene, indicative of activation of cytotoxic T cells and immune responses.

To understand the biology of expanded clones in T-LGLL, we compared gene expression of expanded clones versus remaining cells in individuals in a pair-wise manner in individuals, and top differentially expressed genes were used for pathway analysis by Genomatix^36^. Higher expressed genes in expanded clones were markedly enriched in immune response and cell activation (Fig. 5d), and deregulation of apoptosis pathways was apparent as several key genes in the apoptosis cascade (*FOS*, *BCL2*, and *BIRC3*) were underrepresented in expanded clones. In summary, expanded clones were highly activated and may have acquired growth, survival and functional advantage, partly due to deregulation of apoptosis. The expression characteristics of expanded clones suggest targets for novel therapeutics.

We specifically examined the expression of T cell exhaustion genes and T cell co-inhibitory receptor expression in T-LGLL samples, as these pathways are frequently abnormal in cancers. We consistently observed augmented T cell exhaustion in T-LGLL, using two different reference gene lists^42,43^, but variable alterations of co-inhibitory and co-stimulatory receptors in T cells (Fig. 5e, f)^2,9,44^.

### Immunosuppressive treatment modulates clonality and gene expression in T-LGLL

**Fig. 6:**
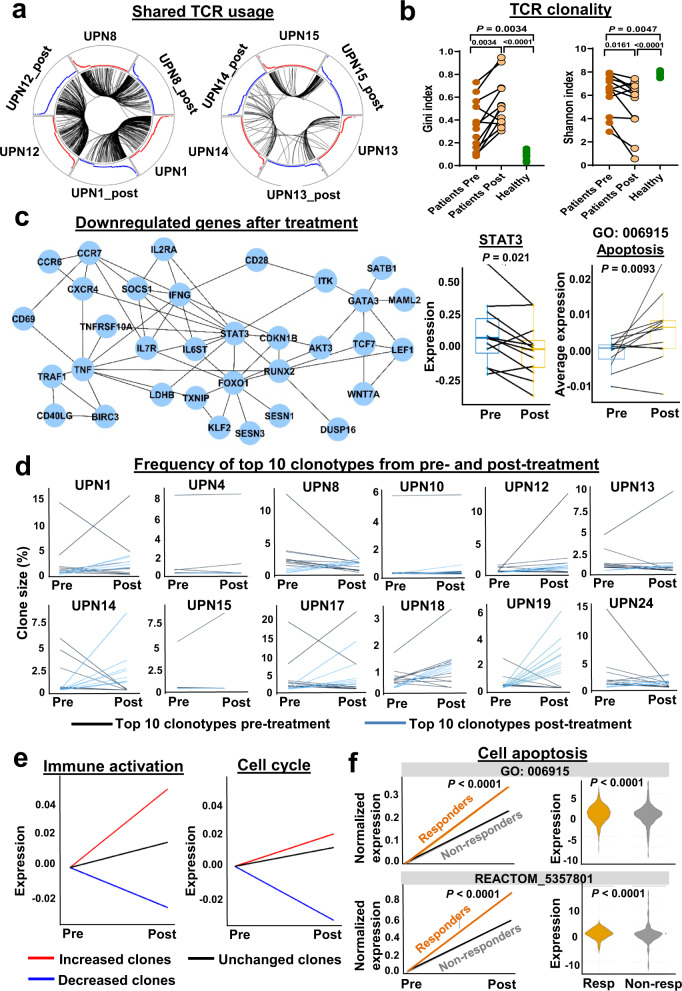
Immunosuppressive treatment modulates clonality and gene expression in T-LGLL. **a** Shown were circos plots, where segments in circles represent individual cells yielding rearranged TCR sequences among patients or between two visits of patients. Black lines indicate arcs connecting cells sharing identical CDR3 sequences. Left and right plots show sharing of identical CDR3 sequences among UPNs 1, 8, and 12, and UPNs 13, 14 and 15, respectively. Red and blue curves are proportional to clone sizes in samples before and after treatment, respectively. **b** Gini index and a Shannon index of TCR clonality were compared in patients (pre- and post-treatment) and with healthy donors. Gini index was still significantly higher (0.547 ± 0.215, *P* < 0.001) and Shannon index significantly lower (5.21 ± 2.25, *P* = 0.002) when compared with those of healthy controls. A two-sided Wilcoxon test between patients’ samples before and after treatment (*n* = 12); two-sided unpaired Mann–Whitney between patients (*n* = 13) and healthy donors (*n* = 7); *P* values shown in the figure. **c** A module of downregulated genes identified by jActiveModulesTopo in T-LGLL patients after treatment, including *STAT3*; dynamic changes of expression levels of *STAT3* and apoptosis genes (GO: 006915) pre- and post-treatment in T-LGLL patients (*n* = 12). Shown are 25–75% response ranges (top and bottom lines of boxes) and minima and maxima (bars). *P* values with two-sided paired t-test were shown in figures. **d** Shown are percentages of top ten TCR clonotypes from pre- and post-treatment samples at different time points. Black lines indicate top ten clones pre-treatment; blue lines indicate top ten clones post-treatment those were not among top ten pre-treatment. **e** Expression changes of immune activation genes and cell cycle genes of increased, decreased and stable clones after alemtuzumab. **f** Left, expression changes of apoptosis genes (averaged) in responders and non-responders after treatment with alemtuzumab. *x*-axis, two time points, pre- and post-treatments; *y*-axis, adjusted expression levels of apoptosis genes to set pre-treatment values of apoptosis gene expression as zero in both responders and non-responders. Right, expression levels of apoptosis genes before treatment in responders (Resp) and non-responders (Non-resp); two-sided unpaired *t*-test; *P* < 0.0001 [as software generated *P* < 0.0001, exact *P* value not available].

By comparing T cell subsets before and after treatments (3 and 6 months) with the monoclonal antibody alemtuzumab, we observed that there was no attenuation of CD8 dominance or of T cell expansion dominated by effector T cells. Alemtuzumab did not significantly change T cell subset proportions nor inflammatory cytokines; the proportion of CD45RO^+^CD8^+^ T cells increased (Supplementary Fig. 25). We examined changes in T cell clonality after alemtuzumab. Many rearranged CDR3 junctions were shared predominantly within subjects, before and after treatment, but not between subjects. Larger clones appeared more likely to persist after treatment (Fig. 6a and Supplementary Fig. 26). Immunosuppression did not attenuate TCR clonality; treatment further skewed TCR diversity, as evidenced by a lower H diversity index compared with prior to treatment (Fig. 6b).

We explored for transcriptomic changes after alemtuzumab by pair-wise comparison of gene expression. Globally, we observed decreased expression of some immune gene sets, including *TNF*, *NFkB*, *KRAS*, *IL2* and *STAT5*, while *MYC* and cell cycle (G2M checkpoint, DNA repair and mitotic spindle) genes were to be upregulated (Supplementary Fig. 27a). However, pathway analysis of top differentially expressed genes showed many immune pathways were upregulated (Supplementary Fig. 27b). *STAT3* was one prominently downregulated genes after treatment and the hub gene in the subnetwork (identified by jActiveModulesTopo) of genes downregulated post-alemtuzumab. Several of its neighboring genes, including *TNFRSF10A, CDKN1B, CXCR4, IL6ST, IL2RA*, and *FOXO1* (all involved in apoptosis), were also downregulated, but average expression of apoptosis genes (GO: 006915) was upregulated after treatment (Fig. 6c). Genes involved in T cell differentiation and proteasome activity were downregulated after treatment. Except for antibody-dependent cytotoxicity and lymphodepletion, alemtuzumab treatment appeared to modulate the STAT3 pathway and to inhibit T cell differentiation; all likely contribute to restrict expansion and activation of residual lymphocytes.

To further characterize clone dynamics after alemtuzumab, we broadly classified the kinetics of T cell repertoire patterns into three groups, based on proportional changes of clones: increased (clone size increased > 20%), unchanged (clone size remained ±20%), and decreased clones (clone size decreased >20%). In 12 T-LGLL patients for whom there were paired data, we observed 26 increased clones (size changes ranging 11–3104), 7 unchanged clones, and 24 decreased clones (size changes ranging 31–4950). The majority of expanded clones persisted after treatment, in responder and non-responder cases. Dynamic changes of top clones in individual patients (Fig. 6d and Supplementary Figs. 28–32) showed four general patterns after treatment: (I) top clones increased or decreased, with new dominant clones (UPNs 1, 13, and 17); (II) top clones replaced by new dominant clones (UPNs 8, 14, 19, and 24); (III) subtle changes of dominant clones (UPNs 4 and 10); (IV) dominant clones further expanded (UPNs 12, 15, and 18). None of these patterns correlated with response to treatment^1^.

To understand how treatment modified CTL clone behavior, we compared gene expression before and after treatment of the same clone in a pair-wise manner, for clones following the three dynamic patterns. With treatment, increased clones showed upregulation of genes enriched in immune response and cell activation (IFNγ, KRAS, MTOCR, IFNα, and JAK/STAT3 pathways), but genes involved in immune response, lymphocyte activation, and cell metabolism (translation initiation, protein localization, respiratory electron transport and cell cycle) were downregulated in unchanged and decreased clones (Fig. 6e and Supplementary Figs. 33 and 34). With immunosuppressive therapy, clones that had increased in sizes retained active immune functions and cell metabolism, while genes with these functions in stable and decreased clones were depressed^23^. In brief, paired scRNA-seq and scTCR-seq showed that alemtuzumab modulated T cell clonality and global transcriptome signatures in T-LGLL. Given minimal alterations in T cell subsets and extensive changes within TCR clones following treatment, we inferred transcriptome alterations in a single cell subset to reflect features of the pathogenesis of T-LGLL.

After alemtuzumab, increased plasma cytokines in T-LGLL patients were similar to pre-treatment levels (Supplementary Figs. 35 and 36). Changes of T cell subsets and TCR clonality also did not differ in responders and non-responders. We sought a discriminator of response to alemtuzumab in dynamic gene expression changes with treatment. We compared gene expression before and after treatments in a pair-wise manner in responders and non-responders, respectively, and observed cell apoptosis genes increased more significantly in responders than in non-responders (Fig. 6f, left). Next, to identify predictors for response to alemtuzumab in our cohort, gene expression of responders and non-responders at baseline was compared. Genes involved in immune response and cell activation (KRAS, IL6_JAK_STAT3, and TP53 pathways) were highly expressed in responders. Expression of genes in cell apoptosis (GO: 0006915 and REACTOM_5357801; Fig. 6f, right). Less skewed activation-induced-cell-death might predict the response to an immunosuppressive therapy, and apoptosis of T cells was a contributor to success of treatment. Alemtuzumab efficacy appeared to be a result of increased expression of apoptosis genes and consequent suppression of clonal expansion.

## Discussion

We utilized coupled T cell transcriptome and TCR repertoire analyses at single-cell resolution to characterize T cell clonal expansion and gene expression in patients with T-LGLL, who had been treated with alemtuzumab in a phase 2 clinical research study. Our results confirmed the clonal expansion of T cells and the lack of common TCR clonotypes in T-LGLL, with paired α and β chain information from single cells. We defined expanded clones (more than ten T cells with an identical TCR clonotype), which usually are diluted in bulk samples, and identified several potential common convergence groups of CDR3 sequences that were more prevalent in T-LGLL patients, but there were no apparent common antigens, at least using currently available databases. TCR repertories in T-LGLL followed the power law distribution, and there was a combined effect of TCR usage and activation states on T cell phenotypes. We noted absence of a common clonotype among patients, as clones dominant in some cases were present in other cases only at low levels, and the same clonotypes also were found in healthy controls. We confirmed deregulation of cell survival and apoptosis gene programs in T-LGLL. We defined four patterns of clonal kinetics with treatment, which correlated to treatment responses. Aleumtuzumab did not attenuate clonal expansion of T cells but increased apoptosis in these cells. Immune activation persisted after treatment, but baseline expression and the extent of apoptosis gene dysregulation correlated with response to treatment, regardless of clonal dynamic pattern.

An (unknown) initiating antigen has been hypothesized to be the etiology of T-LGLL^2^, but we and others have not observed shared clonotypes in CD8^+^ T-LGLL, even in large patient cohorts and among HLA-matched individuals^5^. In GLIPH, some common CDR3 convergence groups were shared, by only a few patients. Imputation of potential antigens corresponding to common expanded CDR3 clusters was not successful due to limits of current reference databases. The majority of top CRGs shared common transcriptome features, particularly upregulated immune activation and cell cycling; these features were also observed in clones that increased after alemtuzumab, concordant with a presumed survival and functional advantage for expanded clones in the stressful environment induced by a monoclonal antibody therapy.

The mechanism of action of alemtuzumab in T-LGLL remains uncertain. Hematologic response is accompanied by reduced total T cell numbers, but persistence of abnormal clones and high cytokine levels^1^. Cytopenias in T-LGLL likely are due to cellular rather than humoral mediation of hematopoietic cell destruction, and alemtuzumab may act by diminution rather than elimination of pathogenic clones. Indeed, we and others have observed persistent pathogenic T cell clones despite effective treatment; for example, in one study reversal of clonal expansion occurred only some years following therapy^24^. More skewed TCR diversity after treatment was initially surprising. However, the T cell repertoire appeared dynamic; fluctuations in clonal dominance, as imputed from CDR3 sequences, were present in about one-third of patients^24^. Increased clonality post-treatment may be alternatively interpreted as the absence of attenuation of clonal expansion, especially due to the high resolution of scTCR-seq. Apparently, decreased clonal diversity may not entail progressive clonal expansion. A broadly reactive monoclonal antibody likely reduces small as well as large clones, and reductions in T cells could be disproportional were there a survival advantage to the pathogenic LGL clone. Indeed, our data implied expanded clones to exhibit an immune activation profile and proliferative genetic programs.

We observed activation of multiple genes in survival signaling pathways and global deregulation of apoptosis in T-LGLL, consistent with published findings^2,3^, with the central hub of this survival network of *STAT3*. STAT3 has a critical function in repressing apoptosis, and inhibition of STAT3 signaling induces apoptosis and decreases survivin expression^45^. Alemtuzumab treatment reduced *STAT3* expression, increased apoptosis, and inhibited T cell differentiation. Suppression of the JAK/STAT3 pathway has been effective in clinical trials in immune-mediated diseases^1,46^. In addition to antibody-dependent cytotoxicity, lymphocytopenia after alemtuzumab treatment may be attributable to inhibition of lymphocyte differentiation and suppression of lymphocyte survival. Among discrete changes of gene expression, increased expression of apoptosis genes with treatment was more pronounced in responders than in non-responders, and responders tended to have higher expression levels of apoptosis genes at baseline. Activation of the apoptosis signaling pathways would be a reasonable mechanism of alemtuzumab efficacy, slowing but not halting CTL proliferation.

Our results and their interpretation of data have limitations. First, the clinical spectrum and sample size of our cohort were necessarily limited, given the rarity of patients, pretreatment before enrollment, the broad clinical heterogeneity of T-LGLL (*STAT3* mutations and HLA background) and the cost of experiments. Second, conclusions concerning common epitopes and potential antigens of TCRs were limited by available databases. Third, the effects of MYC in cell proliferation and apoptosis were contextual. We interpret MYC to be pro-apoptotic in T-LGLL, but its function might be better assessed in vitro. Exactly how alemtuzumab treatment altered STAT3 signaling could also be sought in tissue culture. Fourth, we based our analysis exclusively on T cell compartment, excluding other cell types. In future, if feasible additional single-cell work in larger cohorts with multiple cell types would be desirable.

Consistent and complementary results were obtained by the Helsinki group utilizing high throughput transcriptome and TCR profiling of single cells from T-LGLL patients, and appear in a companion article^47^. Both works complement prior studies and advanced modeling of T cell clonal expansion, and they should provide a comprehensive database of clones and transcriptomes useful in the understanding of cellular and molecular dynamics of T-LGLL and other immune-mediated diseases.

## Methods

### Patient enrollment and sample collection

Blood samples were obtained from 13 T-LGLL patients (www.clinicaltrials.gov NCT00345345) as detailed in Supplementary Methods. Patients were treated with alemtuzumab. Seven age- and sex-matched healthy donors were enrolled as controls after written informed consent^1^. Peripheral blood mononuclear cells (PBMCs) were isolated by Ficoll-Hypaque density gradient centrifugation followed by lymphapheresis. Isolated PBMCs were cryopreserved in liquid nitrogen according to standard protocols until use. T cells were enriched with the EasySep Human T cell Isolation kit (Catalog #100-0695, Stemcells Technologies).

### Flow cytometry analysis of the TCR Vβ repertoire

TCR Vβ repertoires of patients and healthy donors were determined using flow cytometry with the IOTest Beta Mark TCR Repertoire kit (Beckman Coulter), coupled with anti-human CD3 monoclonal antibody in Qdot605 (1:50 dillution, clone UCHT1, Catalog #Q10054, ThermoFisher), anti-human CD4 in V500 (1:50 dillution, clone RPA-T4, Catalog # 560768, BD Biosciences) and anti-human CD8 in Alexa Fluor 750 (1:50 dillution, clone 37006, Catalog # FAB1509S-025, R&D system). Data acquisition was performed on a Becton Dickinson Fortessa and data were analyzed using FlowJo software (Tree Star Inc.).

### Whole transcriptome amplification (WTA), cDNA library preparation, and sequencing

scRNA-seq and scTCR-seq analyses were performed using the 10x Genomics Single Cell Immune Profiling Solution V1.0 according to the manufacturer’s protocols (10x Genomics V(D)J + 5′ Gene Expression). The scRNA libraries were sequenced on an Illumina HiSeq 3000 system using read lengths of 26 bp read 1, 8 bp i7 index, 98 bp read 2. The scTCR libraries were sequenced on an Illumina HiSeq 3000 using read lengths of 150 bp read 1, 8 bp i7 index, 150 bp read 2.

### Preprocessing of paired scRNA-seq and scTCR-seq data

Gene expression of patients’ samples was analyzed individually. Sequencing data from individual samples (patients at baseline and after treatment of 3 and 6 months, and healthy donors) were preprocessed separately using Cell Ranger 2.1.1, including fastq file generation, read alignment, and gene-cell expression matrix calculation^11^. TCR reads were aligned to the GRCh38 reference genome and consensus TCR annotation was performed using the cellranger vdj program. Barcodes with a higher number of Unique Molecular Identifier (UMI) counts more than simulated background were considered as cell barcodes. For each barcode, cellranger performed de novo assembly, and identified productive contigs and their corresponding CDR3 regions and V, D, J, C genes.

### Analysis of the scTCR-seq repertoire

Shannon entropy and Gini index for diversity analysis were calculated with the R package of tCR (https://imminfo.github.io/tcr/)^48^. To identify epitopes and related antigens, we input β-chain CDR3 sequences of T-LGLL patients into TCRmatch^34^, a tool that uses comprehensive k-mer matching approach to identify similar sequences annotated in the Immune Epitope Database (IEDB)^35^. Specifically, we downloaded the docker version of TCRmatch, and all annotations of IEDB, which collected the published TCRs and corresponding epitopes and antigens. TCRmatch calculated the similarity of the input TCR sequence with those in IEDB, and a similar TCR and a corresponding epitope were retrieved. CDR3β amino acid sequences of the top 500 most abundant CDRs of all patients were pooled and used to construct clone network analysis using GLIPH^30^.

### Data dimensionality reduction and clustering with PhenoGraph

Doublets were removed before further analyses. Cells with UMIs (molecular tags that can be applied to detect and quantify the unique transcripts) over 10,000 (potential doublets) and under 500 (potential fragments), or a mitochondrial proportion higher than 10% (potential apoptotic) were excluded. Downstream analyses were performed using the R software package Seurat (http://satijalab.org/seurat/, v2.3.4). Raw reads in each cell were first scaled by library sizes to 10,000 and then log-transformed. To improve downstream dimensionality reduction and clustering, regression out in the Seurat package was used to remove unwanted sources of variation brought by the number of UMIs and percentages of mitochondrial genes^19^. Highly variable genes identified with y.cutoff = 0.5 and selected genes (~1300) were used for principal component analysis (PCA) of high-dimensional data. Top 30 principal components were selected for unsupervised clustering of cells with a Graph-based clustering approach^11^.

Dimensionally reduction and clustering were performed by PCA and visualized with t-distributed stochastic neighbor embedding (t-SNE). We applied sva/Combat for batch correction and found that samples were well mixed after correction, by evaluation with an entropy-based approach. R-package “Rtsne” was used to run the t-SNE algorithm for the batch-corrected data using, the parameters: initial dimensions = 10 and perplexity = 31.

### Cell cluster annotation

We downloaded raw data of GSE93777 to obtain signature genes for the identification of naive, central, and effector T cell populations^22^. Top 250 most population-specific genes were as signatures of subtypes. We used this gene set to define cell types at cell and cluster levels. CD4+, CD8+, and related subtypes were assigned to each cluster based on significance in overlap between T cells and cluster-specific genes (a Fisher’s exact test).

### Diffusion component analysis

A diffusion map was used as a nonlinear dimensionality reduction technique to identify major non-linear components of variation across cells associated with biological processes^11^. The regression analysis between the AUC scores (calculated with signature genes of biological processes, such as differentiation and T cell activation) was performed against an order of cells by diffusion maps to examine their contributions on components of diffusion maps.

### Gene ontology, pathway, and network analyses

Differentially expressed genes were defined with FindMarkers in Seurat. Gene ontology analysis was performed with the R package topGO v2.26 using the algorithm elim, a minimum node size of 10 and genes that passed the filtering threshold^38^, and further included in the STRING network, as a background gene list^39^. Gene Set Enrichment Analysis (GSEA; http://software.broadinstitute.org/gsea) is a widely used pathway analysis tool that determines whether pre-defined gene sets show statistically significant, concordant differences between two biological states. Cytoscape was used to visualize differentially expressed genes and their interactions^40^.

### Reporting summary

Further information on research design is available in the Nature Research Reporting Summary linked to this article.

## Supplementary information

Supplementary information

Description of Additional Supplementary Files

Supplementary Data 1

Supplementary Data 2

Supplementary Data 3

Supplementary Data 4

Supplementary Data 5

Supplementary Data 6

Reporting Summary

## Data Availability

Raw and analyzed sequencing data in this study have been deposited in the NCBI’s Gene Expression Omnibus (under series accession code GSE168859) and Sequence Read Archive (under accession code SRP310547). All other relevant data supporting the findings of this study are available within the article and its Supplementary Information files or from the corresponding author upon reasonable request. Source data are provided with this paper.

## Source data

Source Data

## Code availability

Code supporting this study is available at a dedicated Github repository [https://github.com/shouguog/TLGL] and Zenodo (10.5281/zenodo.5713623).
