# Supplementary material for: Single-cell RNA sequencing coupled to TCR profiling of large granular lymphocyte leukemia T cells
Source: Nat Commun. 2022 Apr 11;13:1982. doi: 10.1038/s41467-022-29175-x (PMC9001664; doi:10.1038/s41467-022-29175-x)
Supplement: Supplementary file 1 — Supplementary information [file 41467_2022_29175_MOESM1_ESM.pdf]

Supplementary Information

for

Single-cell RNA sequencing coupled to TCR profiling of large granular lymphocyte leukemia T cells

Shouguo Gao[1,*], Zhijie Wu[1,*], Bradley Arnold[1], Carrie Diamond[1], Sai Batchu[1], Valentina Giudice[1], Lemlem Alemu[1], Diego Quinones Raffo[1], Xingmin Feng[1], Sachiko Kajigaya[1], John Barrett[1], Sawa Ito[2] & Neal S. Young[1]

[1] Hematology Branch, National Heart, Lung, and Blood Institute, National Institutes of Health, Bethesda, MD, United States

[2] Division of Hematology-Oncology, Department of Medicine, University of Pittsburgh, Pittsburgh, PA, United States

* S.G. and Z.W. contributed equally to this work.

Contents

- **Supplementary Methods**
- **Supplementary Results**
- **Supplementary Tables**

Supplementary Table 1 Clinical characteristics of patients.

Supplementary Table 2 α and β chains detected in single cells.

Supplementary Table 3 TCR specificity groups defined by GLIPH analysis.

- **Supplementary Figures**

Supplementary Fig. 1 Data merging and removal of batch effects.

Supplementary Fig. 2 Characterization of T cell subsets.

Supplementary Fig. 3 Expression of *TRAV* and *TRBV* genes in scRNA-seq.

Supplementary Fig. 4 Skyscraper plots showing Vβ/Vα and matching Jβ/Jα in healthy donors and patients.

Supplementary Fig. 5 Skyscraper plots showing Vβ/Vα and matching Jβ/Jα in patients.

Supplementary Fig. 6 Distribution of CDR3 lengths in healthy donors and patients.

Supplementary Fig. 7 Clone sizes in $CD8^+$ T cells of healthy donors and patients.

Supplementary Fig. 8 Clone sizes in $CD8^+$ T cells of patients.

Supplementary Fig. 9 Clone sizes in $CD4^+$ T cells of healthy donors and patients.

Supplementary Fig. 10 Lack of common T cell clonotypes in T-LGLL patients in our study: top 500 TCR clones.

Supplementary Fig. 11 Lack of common T cell clonotypes in T-LGLL patients in our study: top 1000 TCR clones.

Supplementary Fig. 12 Lack of common T cell clonotypes in T-LGLL patients of three independent studies.

Supplementary Fig. 13 Lack of common T cell clonotypes in T-LGLL patients in ours and an independent study.

Supplementary Fig. 14 Sequences and corresponding weblogs of top TCR specificity groups with more than five different clones.

Supplementary Fig. 15 Sequences and corresponding weblogs of top TCR specificity groups with more than five different clones (continued from Supplementary Fig. 14).

Supplementary Fig. 16 TCR usage shapes T cell phenotypes.

Supplementary Fig. 17 Clonally expanded T cells are mostly effector memory T cells.

Supplementary Fig. 18 Transcriptome analysis of GLIPH-clustered TCRs.

Supplementary Fig. 19 T-LGLL specific CRGs and imputed potential common antigens.

Supplementary Fig. 20 Lack of specific common antigens in T-LGLL.

Supplementary Fig. 21 Gene modules work synergistically in shaping T cell phenotypes.

Supplementary Fig. 22 Dysregulated gene programs in T-LGLL.

Supplementary Fig. 23 Dysregulated pathways in T-LGLL.

Supplementary Fig. 24 Consistency of gene expression detected using scRNA-seq and qPCR.

Supplementary Fig. 25 Changes of T cell subsets after treatment.

Supplementary Fig. 26 Immunosuppressive treatment modulates clonality in T-LGLL.

Supplementary Fig. 27 Pathway analysis of differentially expressed genes in patients post-treatment vs. pre-treatment.

Supplementary Fig. 28 Four patterns of clonal kinetics of patients pre- and post-treatments (Pattern I).

Supplementary Fig. 29 Four patterns of clonal kinetics of patients pre- and post-treatments (Pattern II).

Supplementary Fig. 30 Four patterns of clonal kinetics of patients pre- and post-treatments (Pattern II, continued from Supplementary Fig. 29).

Supplementary Fig. 31 Four patterns of clonal kinetics of patients pre- and post-treatments (Pattern III).

Supplementary Fig. 32 Four patterns of clonal kinetics of patients pre- and post-treatments (Pattern IV).

Supplementary Fig. 33 Upregulated GO terms in dynamically changed clones.

Supplementary Fig. 34 Downregulated GO terms in dynamically changed clones.

Supplementary Fig. 35 Cytokine levels in healthy donors, and patients pre- and post-treatments.

Supplementary Fig. 36 Top four cytokine genes significantly higher in T-LGLL patients.

- **Supplementary References**

Supplementary Methods

Patient enrollment and sample collection. Blood samples were obtained from 13 T-LGLL patients (71/F, 61/M, 29/F, 72/F, 77/M, 43/M, 51/F, 82/M, 51/M, 27/F, 66/F, 48/F and 39/M) after written informed consent under the protocol (www.clinicaltrials.gov NCT00345345) approved by the Institutional Review Boards of National Heart, Lung, and Blood Institute, in accordance with the Declaration of Helsinki. Patients consented to deidentified used of clinical and research data for publication. Recruitment of patients, diagnostic procedures, treatment and clinical criteria for response to treatment have been described in our alemtuzumab trial[1]. Patients were treated with alemtuzumab (administered at 10 mg/day intravenously for 10 days). A primary endpoint was hematologic response at three months after treatment. A complete response (CR) was defined as normalization of all affected lineages, and a partial response (PR) was defined in neutropenic subjects as 100% increase in the ANC to > 500/uL, and in those with anemia, any increase in hemoglobin of 2 g/dL or more observed in at least two serial measurements one week apart and sustained for one month or more without exogenous growth factors' support or transfusions. Clinical and laboratory characteristics of patients are shown in Supplementary Table 1. Seven age- and sex-matched healthy donors (39/F, 71/F, 55/F, 68/M, 41/F, 60/M and 41/M) were enrolled as controls after written informed consent.

Peripheral blood mononuclear cells (PBMCs) were isolated by Ficoll-Hypaque density gradient centrifugation followed by lymphapheresis in patients before enrollment, 3 or 6 months after alemtuzumab administration and in healthy donors. Isolated PBMCs were cryopreserved in liquid nitrogen according to standard protocols until use. T cells were enriched with the EasySep Human T cell Isolation kit (Stemcell Technologies), with purity (detected by flow cytometry staining with anti-human CD3) after enrichment > 95% (Supplementary Fig. 1a).

Flow cytometry analysis of the TCR Vβ repertoire. TCR Vβ repertoires of patients and healthy donors were determined using flow cytometry with the IOTest Beta Mark TCR Repertoire kit (Beckman Coulter), coupled with CD3, CD4 and CD8 expression. Data acquisition was performed on a Becton Dickinson Fortessa and data were analyzed using FlowJo software (Tree Star Inc.). At least 500 events per $CD4^+$ or $CD8^+$ cell population were acquired per TCRBV to ensure that a sufficient number of T cells were obtained. T-LGLL clones were identified based on large clonal $CD8^+$ or $CD4^+$ TCRBV expansion when compared to a normal range of TCRBV values generated with healthy donors' data. TCRBV clonal analysis was part of exploratory analysis.

***STAT3* mutation analysis.** $CD8^+CD57^+$ cells of patients were isolated using the MACS CD8 T cell isolation kit, followed by positive selection with CD57 microbeads (Miltenyi Biotec), according to the manufacturer's instructions. Subsequently, DNA was extracted using the Maxwell 16 blood DNA purification kit (Promega, Madison, Wisconsin). Sanger sequencing was utilized to analyze *STAT3* mutations. PCR primers were designed to amplify all coding exons (Exons 19 – 32) of the SH2 (Src homology 2) domain of the *STAT3* gene. The extracted DNA was subjected to PCR amplification with adequate primers using the TaKaRa LA Taq polymerase kit (Takara Bio), followed by DNA purification. Using the purified products, a sequencing reaction was performed with adequate sequencing primers and the BigDye Terminator v3.1 Cycle Sequencing kit. After removal of excess dye terminators, sequencing analysis was carried out using the 3130xl Genetic Analyzer (Applied Biosystems). All *STAT3* mutations were detected by bidirectional sequencing. The following primers were used for both PCR amplification and Sequencing. Exons 19 and 20 (F, 5′-AGGGAAGGGCTGGGATGGCA-3'; R, 5'-ATCTCCACCCACCAGGGGGC-3'); exon 21 (F, 5'-GCCAGGCCACTGAACAGGGTG-3'; R, 5'-TCCCATCGGTCACCCCAACA-3'); and exon 22 (F, 5'-TCCTGCCGAGGCAGATGGCT-3'; R' 5'- AGAGCATCACACAAAGGGGACCA-3').

Whole transcriptome amplification (WTA), cDNA library preparation and sequencing. Single-cell RNA sequencing (scRNA-seq) and single-cell TCR sequencing (scTCR-seq) analyses were performed using the 10x Genomics Single Cell Immune Profiling Solution V1.0 according to the manufacturer's protocols (10x Genomics V(D)J + 5′ Gene Expression). In brief, enriched $CD3^+$ T cells were washed and resuspended in PBS + 0.04% fetal bovine serum. Following reverse transcription and cell barcoding in droplets, emulsions were broken and cDNA was purified using Dynabeads MyOne SILANE, followed by PCR amplification. Amplified cDNA was then used for both 5′ gene expression library construction and TCR enrichment. For the gene expression library construction, the amplified cDNA was fragmented, end-repaired and double-sided size-selected with SPRIselect beads. For TCR library construction, TCR transcripts were enriched from the amplified cDNA by PCR. Then, the enriched PCR product was fragmented, end-repaired and size-selected with SPRIselect beads. The scRNA libraries were sequenced on an Illumina HiSeq 3000 system using read lengths of 26 bp read 1, 8 bp i7 index, 98 bp read 2. The scTCR libraries were sequenced on an Illumina HiSeq 3000 using read lengths of 150 bp read 1, 8 bp i7 index, 150 bp read 2.

Preprocessing of paired scRNA-seq and scTCR-seq data. We first analyzed gene expression of all patients' samples individually. Sequencing data from individual samples (patients at baseline and after treatment of 3 and 6 months, and healthy donors) were preprocessed separately using Cell Ranger 2.1.1, available from 10x Genomics (https://support.10xgenomics.com/single-cell-gene-expression/software/pipelines/latest/what-is-cell-ranger), including fastq file generation, read alignment and gene-cell expression matrix calculation. After preprocessing raw data, alignment and enumeration of

reads, a single gene-cell expression matrix was obtained for each sample. The estimated cell number was 800-45,000 per sample.

TCR reads were aligned to the GRCh38 reference genome and consensus TCR annotation was performed using the cellranger vdj program (10x Genomics, version 2.1.0). TCR libraries were sequenced at a depth of over 2000 reads per cell, with a final average of 20798 reads per cell. On average, 81% of reads were mapped to either the TRA or TRB loci in each cell. TCR annotation was performed using the 10x cellranger vdj pipeline as described at https://support.10xgenomics.com/single-cell-vdj/software/pipelines/latest/using/vdj. Barcodes with a higher number of Unique Molecular Identifier (UMI) counts more that simulated background were considered as cell barcodes. V(D) J read filtering and assembly were implemented as a previous study[2]. In brief, cellranger firstly trimmed known adaptors and primer sequences from the 5′ and 3′ ends of reads and then filtered away reads that lacking at least one 15 bp exact match against at least one reference segment (TCR, TRA and TRB gene annotations in Ensembl version 87). Next, for each barcode, cellranger performed de novo assembly by building a De Bruijn graph of reads independently. The assembler outputs the contig sequences which are assigned at least one UMI. Finally, each assembled contig was aligned against all of the germline segment reference sequences of V, D, J, C and 5′ UTR regions. cellranger searched a CDR3 motif (Cys-to-FGXG/WGXG) in a frame defined by the start codon in the L+V region or all 6 frames when the L+V region was absent. A contig was kept and considered as productive if: 1) it fully spanned the V and J segments; 2) there was a start codon in the V region; 3) it contained a CDR3 region in-frame with the V start codon; 4) there was no stop codons in the V-J spanning region. Most cell barcodes contained two matching productive contigs, comprising either a TCRA or a TCRB though it is of biological possibility that fewer productive contigs (low sensitivity) or > 2 productive contigs (some cells do contain more than one TCRB or TCRA chains) were associated with one cell barcode[3].

Data dimensional reduction and clustering with PhenoGraph. Doublets were removed before further analyses. Cells with UMIs (molecular tags that can be applied to detect and quantify the unique transcripts) over 10,000 (potential doublets) and under 500 (potential fragments), or a mitochondrial proportion higher than 10% (potential apoptotic) were excluded. Downstream analyses were performed using the R software package Seurat (http://satijalab.org/seurat/, v2.3.4). Raw reads in each cell were first scaled by library sizes to 10,000 and then log-transformed. To improve downstream dimensionality reduction and clustering, regressionOut in the Seurat package was used to remove unwanted sources of variation brought by the number of UMIs and percentages of mitochondrial genes[4]. Then, highly variable genes identified with y.cutoff = 0.5 and selected genes (~1300) were used for Principal Component Analysis (PCA) of high-dimensional data. Top 30 principal components were selected for unsupervised clustering of cells with a Graph-based clustering approach[2,4].

Dimensionally reduction and clustering were performed by PCA and visualized with t-distributed stochastic neighbor embedding (t-SNE). With t-SNE plots of cells from all samples, cells of the same subject tended to gather together due to subject specificity and batch effects (Supplementary Fig. 1b)[5,6], and therefore identification and removal of batch effects and other unwanted sources of variation were needed. This was as expected as large-scale scRNA-seq data sets that were produced with different libraries and at different times contained batch effects that may compromise integration of the data for comparisons of samples. Though canonical correlation analysis (CCA) in Seurat and MNN algorithms are widely used in scRNA-seq data analysis, they cannot handle data with millions of cells, due to huge memory usage and computation complexity[6]. We applied sva/Combat for batch correction and found that samples were well mixed after correction, by evaluation with an entropy-based approach (Supplementary Fig. 1f, g. R-package "Rtsne" was used to run the t-SNE algorithm on the batch corrected data using the following parameters: initial dimensions = 10 and perplexity = 31.

After batch correction, $CD4^+$ and $CD8^+$ cells were grouped together and the clustering was little contributed by individual subject effects. After correction for batch effects and individual sample variation, $CD4^+$ and $CD8^+$ cells formed two groups, and cells from different subjects were well mixed and separated by cell type categories (Fig. 1b and Supplementary Fig. 1c, d).

Automated phenotypic description of cell clusters. To obtain cell clusters, PhenoGraph (a clustering method designed for high-dimensional single-cell data by creating a graph representing phenotypic similarities between cells and then identifying communities in this graph) was run on a batch corrected dataset, using k = 30 nearest neighbors (a default parameter). This resulted in 125 clusters, and the number was high for annotation. To simplify the cluster annotation, we conducted a second application of PhenoGraph to the previously defined PhenoGraph clusters from all cells[2]. Expression of each cluster was represented by its centroid computed by taking a median of genes across all cells in the cluster. PhenoGraph was run on a cluster expression matrix with a parameter k = 15. Finally, PhenoGraph partitioned 125 clusters into 10 metaclusters, which all had a mixed patient composition. With annotated $CD4^+$ and $CD8^+$ signature genes, metacluster 0, 4, 5, 6 and 8 were assigned for $CD8^+$ cells and metacluster 1, 2, 3, 7 and 9 were assigned for $CD4^+$ cells (Supplementary Fig. 1d).

Cell clusters annotation. We downloaded raw data of GSE93777 for signature gene identification of naïve, central and effector T cell populations[7]. In specific, we used one-sided t test to compare gene expression of subtypes against the rest samples to define top 250 most population specific genes as signatures of subtypes. We used this gene set to define cell types at cell and cluster levels. $CD4^+$, $CD8^+$ and related subtypes were assigned to each cluster based on significance in overlapping between T cells and cluster-specific genes (a Fisher's exact test)[7]. More specifically, top 250 overexpressed genes in each T cell subtype population were obtained from GSE93777 of GEO and were considered as cell type

specific signature genes. Subsequently, one-tailed Fisher's exact test was utilized to assert enrichment of T cell subset signature genes in a cluster marker gene list for each cluster, and a top associated cell type was assigned to each cluster.

AUCell for signature assessment of individual cells. Besides assigning cell types at cluster levels, we also used the AUCell package in Bioconductor, which computes the "Area Under the Curve" (AUC) of gene sets for individual single cells, using the same gene sets from GSE93777 as above to annotate cell types at cell levels[7]. This AUCell score reflects the possibility of each cell for a certain cell type. The input to AUCell is a gene set, and the output is the gene set 'activity' in each cell. In brief, the scoring method is based on a recovery analysis which considers ranking of all genes based on expression levels (genes with the same expression values, e.g., '0', are randomly sorted). AUCell then uses AUC to calculate whether a critical subset of the input gene set is enriched at a top of the ranking for each cell. In this way, the AUC represents proportions of expressed genes in the signature and their relative expression values compared to the other genes within the cell. AUCell assigns an AUCell score to each cell which shows how a critical subset of the input gene set is enriched within the expressed genes for each cell. In assigning cell types, results using the AUCell method (at cell levels) and the above method on cluster levels showed good consistency (Supplementary Fig. 1d, e). Further, we computed AUCs for gene sets associated with the signaling and immune pathways to quantify cell's pathway activity (termed a transcriptional phenotype in this study).

Entropy metric to evaluate batch effect correction and mixing of samples in clusters. To evaluate combat/sva's ability to correct batch effects across data from all healthy donors and T-LGLL patients, we devised an entropy-based metric that quantifies mixing of the normalized data across samples. The entropy-based metric was computed as follows: We constructed a k-NN graph (k = 30) on the

normalized data using Euclidean distance and clustered cells with phonographs in patients and health controls $m = 1, \dots 32$. For each cluster j, we calculated a fraction of cells from a subject m (normalized by the cell number in each sample), denoted as q_j^m. Then we computed Shannon entropy $H_j = -\sum_{m=1}^{32} q_j^m \, log q_j^m$ as a measure of mixing between patients. We observed that clusters displayed differing amounts of mixing between samples before batch correction. The mixture of samples was highly increased after batch correction with sva (Supplementary Fig. 1f, g).

A diversity index and power law curve fitting. There are many ways of defining the diversity of a population, clonal types in this study, with each method providing a different representation of the number of clones (identical TCR chains), present (richness) and of their relative frequency (evenness). Shannon entropy weighs both of these aspects of diversity equally is an intuitive measure whereby the maximum value is determined by a total size of the repertoire. Entropy values decrease with increasing inequality of frequency as a result of clonal expansion. The Shannon entropy in a population of N clones with frequency $p_{1,2,\dots,N}$ is defined by equation (1):

$$H(P) = -\sum_{i=1}^{N} p_i log_2 p_i \qquad (1)$$

A Gini coefficient is a number aimed at measuring the inequality in a distribution. It is most often used in economics to measure a country's wealth distribution and has been widely used in diversity assessment of TCRs[8]. The Gini coefficient is usually defined mathematically based on the Lorenz curve or Relative mean absolute difference[9].

The Shannon entropy and Gini index for diversity analysis were calculated with the R package of tCR (https://imminfo.github.io/tcr/).

Inferring power law exponents from empirical data are non-trivial due to severe biases incurred by linear regression on bi-logarithmic scales, especially considering the heavy trail distribution[10]. We employed a maximum likelihood framework with an iterative numerical optimization method to fit the

power law distributions and infer the power law exponents. A slope of fitted lines, i.e. an estimated power law exponent was used to evaluate expansion scales of TCR clones in certain subjects. We fitted the power law distributions with paired AB chains, or A and B chains, respectively. Since the clonal expansion of T-LGLL mainly happens in a $CD8^+$ population, we separated $CD4^+$ and $CD8^+$ cells, respectively, in analysis.

Other T-LGLL TCR datasets and epitope identification. Due to the small number of patients in this study, we collected the published T-LGLL TCR datasets for integrative analysis. Top sequences of β chains were retrieved from two papers[11,12]. The Immunoseq dataset of the third study was downloaded from https://clients.adaptivebiotech.com/pub/kerr-2019-bloodadvances. We used these three datasets to validate our results of clonality and lack of common TCR clones. To identify epitopes and related antigens, we input β-chain CDR3 sequences of T-LGLL patients into TCRmatch[13], a tool uses comprehensive k-mer matching approach to identify similar sequences annotated in the Immune Epitope Database (IEDB)[14]. Specifically, we downloaded the docker version of TCRmatch, and all annotation of IEDB, which collected the published TCRs and corresponding epitopes and antigens. TCRmatch calculated the similarity of the input TCR sequence with those in IEDB, and a similar TCR and a corresponding epitope were retrieved.

TCRβ cluster analysis. CDR3β amino acid sequences were obtained through pooling top 500 most abundant CDRs of all patients and were used to construct clone network analysis using GLIPH[15]. GLIPH clusters TCRs based on global similarity, that is, CDR3B fragments within the same cluster are required to be different by one amino acid at most, and this difference must be at the same amino acid location in all fragments within the cluster. Based on the presence of unique motifs in a given dataset, GLIPH was used to calculate a probability of the occurrence of these unique motifs relative to their

expected frequency in a naïve TCR dataset. We included the HLA types, an important factor affecting the TCR3 sequence, in GLIPH analysis. WebLogo (https://weblogo.berkeley.edu/logo.cgi) was used to generate sequence logos, with columns of amino acids for each position in the sequence. A column height represented conservation of the sequence at that position, while a height of the amino acid within the column showed relative frequency.

Diffusion component analysis. A diffusion map was used as a nonlinear dimensionality reduction technique to find major non-linear components of variation across cells to be associated with biological processes[2]. Top ten diffusion components were computed using the destiny Bioconductor package, which implements diffusion maps as described in Philipp Angerer et al[16]. We selected t = 1 diffusion steps and this approximated diffusion of information for each cell through its 20 nearest neighbors in our data. Due to the computation complexity, we randomly chose 500 cells from each sample for a diffusion map. We repeated the random choice and obtained the same results.

To check the pathways contributing cell transcriptional phenotypes, we used the regression analysis between the AUC scores (calculated with signature genes of biological processes, such as differentiation and T cell activation) against an order of cells by diffusion maps to examine their contributions on components of diffusion maps.

Differential expression of genes and heatmap generation. Differentially expressed genes were defined with FindMarkers in Seurat, by comparing gene expression in one cell subset with that in all others. Genes with P value < 0.05 and Log (average fold change) > 0.1 were regarded as differentially expressed genes. Heatmaps and network visualization were generated with ggplot2 and heatmap2 in the R package.

Gene ontology, pathway and network analyses. Gene ontology analysis was performed with the R package topGO v2.26 using the algorithm elim, a minimum node size of 10 and genes that passed the filtering threshold, and further included in the STRING network, as a background gene list[17]. Set Enrichment Analysis (GSEA; http://software.broadinstitute.org/gsea) is a widely used pathway analysis tool that determines whether pre-defined gene sets show statistically significant, concordant differences between two biological states. Fast GSEA (FGSEA; https://bioconductor.org/packages/release/bioc/html/fgsea.html) was performed via the fgsea R/Bioconductor package. An expression level change of each gene was used as a ranking metric input with the REACTOME and KEGG pathways collected in the Molecular Signatures Database (MSigDB). The Cytoscape plugin jActiveModulesTopo was utilized to identify expression-active connected subnetworks in a gene association network collected in STRING[18,19]. A top scoring module was selected as the most related subnetwork.

Real-time reverse transcriptase PCR (RT-PCR). A PCR array 384 well created by Qiagen (Frederick, MD) was used to check expression of 84 genes for the JAK-STAT signaling pathway (PAHS-039ZE-4). Total RNA was extracted from magnetic bead-sorted cells using the Qiagen RNeasy Mini kit, converted to complementary DNA and used for the PCR Array. Data analysis was accomplished using the ΔΔCt method (Qiagen DataAnalysis WebPortal, https://geneglobe.qiagen.com/us/analyze).

Supplementary Results

scRNA-seq of T cells in T-LGLL patients. We had obtained large cell collections from 13 T-LGLL patients who had participated in a clinical trial of a monoclonal antibody, alemtuzumab[1]; most had refractory disease and therefore had been treated with other modalities earlier (Supplementary Table 1). $CD3^+$ cell populations were subjected to scRNA-seq and TCR profiling using the 10x Single Cell V(D)J + 5′ Gene Expression platform (Fig. 1a). Metrics for scRNA-seq and TCR profiling of 32 samples are shown in Supplementary Data 1, respectively.

Cell clustering. A standard approach to assign cell types in scRNA-seq is to cluster cells by similarity of transcripts and then to impute cell identity by comparison of highly expressed genes in each cluster to known signature gene sets.

There are numerous algorithms of clustering, but there is no clear one which performs better than others. However, PhenoGraph, which is originally developed to cluster Cytof data, has been adapted to scRNA-seq and appears to have gained community appreciation with a good reputation, and it is implemented in Seurat, cellranger and Scanpy[2]. We first verified a presence of $CD4^+$ and $CD8^+$ cell types using PhenoGraph clustering. We used AUCell to quantify our CD4 and CD8 signature gene sets for per single cells (AUC representing proportions of expressed genes in the signatures and their relative expression compared to all other genes). As expected[20], we observed variation in a immune cell composition of each subject (Supplementary Fig. 2): a $CD4^+$ T cell and a T cell fractions constituted 12 - 73%, respectively. A $CD8^+$ T cell fraction was between 27% and 88% (Fig. 1d).

Integration of data across all subjects to build a T cell atlas for T-LGLL. We merged data from $CD3^+$ cells of all samples for systematic comparison across patients and healthy donors. We observed that cells from the same patient were often more similar than cells of the same lineage across patients

(Supplementary Fig. 1b). Clustering of cells was likely influenced by both batch effects and sample specificity. To remove technical effects, sva/ComBat was used to remove batch effects, and after batch normalization, $CD4^+$ and $CD8^+$ T cells were grouped, resulting in little evidence of individual subject effects. (Fig. 1b and Supplementary Fig. 1).

We used entropy to quantify patient specificity and found that the clusters varied widely in their degrees of patient mixing. Compared with the entropy-based measure of permutated data, while cells within individual samples were still more similar, batch effects were greatly corrected by sva, with significantly improved mixing of cells across patients ($P < 0.0001$; Supplementary Fig. 1f, g). After correction for batch effects and individual sample variations (see Supplementary Methods), $CD4^+$ and $CD8^+$ T cells formed two groups (Fig. 1b). PhenoGraph, yielded a total of 125 clusters, and the numbers were too large for interpretation. Therefore, we represented each cluster by its centroid and used second-round PhenoGraph to group centroids into metaclusters. Finally, we obtained 10 metaclusters, in which metacluster 0, 4, 5, 6 and 8 were for $CD8^+$ T cells and metacluster 1, 2, 3, 7 and 9 were for $CD4^+$ T cells (Supplementary Fig. 1d).

Finally, we found that a high variation in the immune cell composition of each patient (Fig. 1f). CD8 expression, for example, was relatively uniform among cells from healthy donors, in contrast in T-LGLL, some patient samples showed increased CD8 expression (Fig. 1c). This was expected because the clonal expansion of $CD8^+$ T cells in T-LGLL resulted in a higher number of CD8 cells. There was not difference between T-LGLL and healthy donors in CD4 expression, indicating that no extreme clonal expansion in a $CD4^+$ T cell population. As expected, clonal expansion happened mainly in $CD8^+$ T cells but not in $CD4^+$ T cells, consistent with results of flow cytometry.

To construct a global atlas of cells and their biological annotations, we merged data across all cells from healthy donors and patients, revealing diverse sets of 125 clusters from PhenoGraph.

To assign each cluster to a cell type, we utilized a Fisher test and identified subtypes of $CD4^+$ and $CD8^+$ T cell clusters. Annotations were confirmed manually from expression of canonical markers (Supplementary Fig. 2b). $CD8^+$ and $CD4^+$ clusters were further split into naive, central memory, effector memory and Treg subclusters. We identified the most known major $CD3^+$ immune cell types, including naïve, memory and effector populations in $CD4^+$ and $CD8^+$ T cells (Supplementary Fig. 2). This provides the research community a large atlas of T cells for T-LGLL research in future.

Concordance of *TCRB* gene expression detected in scRNA-seq and scTCR-seq. Same as other studies, the *TRAV* genes had lower expression than *TRBV* genes do (Supplementary Fig. 3a; $P = 0.04$), which leads to a low detection rate of α chain in scTCR-seq. We compared counts of cells with TCR encoded by different *TRBV* genes and expression levels of corresponding *TRVB* genes in scRNA-seq. There was a high correlation between counts of TCR-captured cells and scRNA-seq expression of TRBVs, demonstrating concordance of scRNA-seq and scTCR-seq, and high quality of our datasets (Supplementary Fig. 3b).

Loss of TCR repertoire diversity demonstrated by abnormal size distribution of CDR3. Lengths of CDR3 regions may affect TCR structures and thus T cell functions[21]. We defined normal CDR3 size profiles by comparing CDR3 size distributions among healthy donors (Fig. 2f). In both $CD4^+$ and $CD8^+$ T cells, CDR3 sizes were typically distributed in a Gaussian manner, with 10 - 12 different size classes of 30 - 60 nucleotides (equivalent 10 - 20 amino acids) at 3 nucleotide intervals. In T-LGLL patients, lengths of the most frequent β-CDR3 sequences ranged from 13 to 15 amino acids[21]. Sequence patterns in some T-LGLL resembled those in healthy donors. But in patients with T cell monoclonal or oligoclonal expansion, CDR3 sizes showed abnormal size distribution patterns and were concentrated in a few sizes (16 amino acids for UPN10) (Fig. 2f and Supplemental Fig. 6).

TCR repertoires in T-LGLL follow a power law distribution but at a larger scale. A single cell approach allows analysis of paired αβ sequences of TCR. We quantified a clone size distribution of paired αβ CDR3 sequences[10,22]. In both patients and healthy donors, $CD8^+$ T cells' clone size frequency distributions of both single and paired αβ chains fitted a heavily tailed power law distribution (Supplementary Figs. 7 and 8), characterized by a linear behavior on a bi-logarithmic scale (a negative linear relationship between logarithmic expression of clone frequency and clone sizes; Fig. 2g). Compared to healthy donors, there were some very large clones in patients. In a power law distribution, a slope corresponds to an exponent in the power law and, in effects, is a measure of population diversity. Therefore, we used a slope as a metric to quantify TCR repertoire diversity: the greater a value of the slope, the higher diversity. We observed higher slope values in patients as compared to those in controls (Fig. 2h), again indicating loss of diversity and clonal expansion of T cells in T-LGLL. There were different power law distributions between $CD4^+$ and $CD8^+$ T cell. A $CD8^+$ T cell repertoire deviated from the power law behavior at the tail. Most samples had insufficient $CD4^+$ T cells with detected CDR3 sequences to fit a power law relationship, but there was an approximate negative relationship between clone sizes and frequency in assessed samples (Supplementary Fig. 9). These results supported a predominance of $CD8^+$ T cell clonal expansion in T-LGLL.

Characteristics of cytomegalovirus (CMV) and *STAT3* mutations in T-LGLL patients. It was suggested that T-LGLL lymphocytosis is likely to be the result of long-term stimulation by viral antigens[23]. Chronic CMV antigen stimulation has been postulated as a potential driver for T-LGLL lymphocytosis as its reactivation has been associated with T-LGLL lymphocytosis[23]. In our study, clonal expansion in patients who had serologic evidence of CMV infection was much higher than in seronegative patients ($P = 0.05$). There was no association between *STAT3* mutations and clonal expansion ($P = 0.74$).

Among 13 patients, eight had *STAT3* mutations and eight showed serologic evidence of CMV infection. We found a marginal negative correlation between the presence of a *STAT3* mutation and CMV infection (and T cell clonal expansion associated with this prevalent virus; $P = 0.09$). Patient ages were not associated with clonal expansion ($P = 0.33$).

CDR3 sequence annotation with VDJdb. To characterize other potential common antigens (and microbes), we imputed viral epitope binding from CDR3 sequences by comparison of reported virus-specific CDR3 sequences. In both patients and healthy donors, the most prevalent virus-specific CDR3 sequences were contributed from CMV, EBV, InfluenzaA, HomoSapients, HIV-1, DENV, YFV, MCMV, SARS-CoV-2, M.tuberculosis, HCV, LCMV, PlasmodiumBerghei, SIV and HTLV (Supplementary Fig. 20a), but they only constituted up to 1.26% of total T cells in patients and healthy donors, respectively. There was a good correlation of these virus-specific CDR3 sequence frequency in patients and in healthy donors (Supplementary Fig. 20b); only two had marginally higher frequency in patients than in healthy controls (EBV:EBNA3A:FLRGRAYGL, with $P = 0.07$; and CMV:pp65:NLVPMVATV, with $P = 0.09$). Clonal expansion in T-LGLL therefore could not be explained by exposure to common known viruses. (Our analysis was limited by the small number of subjects in the VDJdb database; large-scale curated repositories would enhance antigen identification from TCR profiling.)

MYC in T-LGLL. MYC's effects of proliferation and apoptosis are indeed contextual. In the current study, we focused on its pro-apoptotic function for the following reasons. First, T-LGLL cells are resistant to activation-induced cell death, and apoptosis is considered important in T-LGLL pathogenesis[24,25]. MYC can control T cell death via FasL[26]. Second, because MYC expression in T-LGLL was too low for accurate measurement, we instead created a network with MYC and its

neighboring genes in STRING (https://string-db.org/). From this network, two subnetworks were created for genes with only apoptosis or proliferation functionality (an apoptotic network) or proliferation (a proliferation network). Then we calculated the activities of the gene subnetworks with AddModuleScores in Seurat for each cell. ANOVA analysis of healthy, pre- and post-treatment samples showed significant difference in activities of the apoptotic network but not the proliferation network. Thus integrative analysis of MYC and its neighboring genes suggested proapoptotic functions of MYC in T-LGLL, which can be tested in vitro (as with *MYC* knockdown in T-LGLL cells). Function of MYC in the context of T-LGLL could be better assessed by functional experiments, but in vitro assays with primary samples are not easy to do (partly due to heterogeneity of samples, with or without *STAT3* mutations, TCR clones and so on), and also does not necessarily reflect pathophysiology in vivo.

Supplementary Table 1 Clinical characteristics of patients.

UPN	Age	Sex	Hematologic presentation	Prior therapies	Response to treatment		STAT3 mutation	T-cell receptor gene PCR		Clone size (% in T cells)	CMV IgG prior to treatment	Samples
					3M	6M		Immunodominant clone				
1	51	F	Neutropenia	Pred, CsA, splenectomy, growth factors	CR	CR	c.1840A>C (p.Ser614Arg)	TRVβ 13.6	Oligoclonal	87.6	Positive	Pre, 3M_CR
4	77	M	Anemia	None	PR	Relapsed	None	TRVβ 2	Monoclonal	76.6	Positive	Pre, 3M_PR
6	39	M	Anemia	MTX, CsA, CTX, ATG, predisone	NR	NE, off	Normal	TRVβ 2	Monoclonal	9.5	Negative	Pre
8	51	M	Anemia	Fludarabine, CsA	CR	CR	None	TRVβ 8	Monoclonal	34.9	Positive	Pre, 6M_CR
10	61	M	Neutropenia	MTX, CsA, spenectomy, prednisone	CR	CR	c.1919A>T (p.Tyr640Phe)	TRVβ 7.2	Monoclonal	26.6	Positive	Pre, 3M_CR
12	82	M	Anemia	CsA, MTX, CTX, growh factors	CR	CR	None	TRVβ 8	Monoclonal	70.3	Negative	Pre, 3M_CR
13	27	F	Anemia	CsA, IVIG, growth factors, rituximab	PR	PR	c.1919A>T (p.Tyr640Phe)	TRVβ 13.2	Monoclonal	14.3	Negative	Pre, 6M_PR
14	66	F	Neutropenia	MTX, CsA, CTX	NR	PR	None	TRVβ 2	Monoclonal	42.3	Negative	Pre, 6M_PR
15	48	F	Pancytopenia	MTX, Predisone, CTX, CsA, growh factors	NR	NR	c.1981G>T (p.Asp661Tyr)	TRVβ 2	Monoclonal	19.3	Positive	Pre, 6M_NR
17	43	M	Anemia	MTX, CsA, growth factors	NR	NR, off	c.1981G>T (p.Asp661Tyr)	TRVβ 14	Oligoclonal	44.1	Positive	Pre, 6M_NR
18	72	F	Anemia, neutropenia	MTX, CsA, growth factors	PR	Relapsed	None	TRVβ 8	Oligoclonal	32.4	Negative	Pre, 3M_PR
19	29	F	Anemia	MTX, Prednisone, CTX	PR	PR	None	TRVβ 17	Monoclonal	79	Negative	Pre, 3M_PR
24	71	F	Anemia	Pred, MTX, CsA, rituximab, CTX	CR	CR	c.1981G>T (p.Asp661Tyr)	TRVβ 23	Monoclonal	33.9	Positive	Pre, 3M_CR

Supplementary Table 1 Clinical characteristics of patients (continued).

UPN	HLA type
1	HLA-A*33:HLA-A*101:HLA-B*18:HLA-B*51:HLA-Cw*121:HLA-Cw*402:HLA-DRB1*10011501:HLA-DRB*5*01:HLA-DQ*0501:HLA-DQ*06
4	HLA-A*01:HLA-A*0201:HLA-B*08:HLA-B*44:HLA-Cw*05:HLA-Cw*07:HLA-DRB_*3*01:HLA-DRB1*01:HLA-DRB1*03:HLA-DQ*02:HLA-DQ*05
6	HLA-A*24:HLA-A*31:HLA-B*07:HLA-B*08:HLA-Cw*07:HLA-DRB1*01:HLA-DRB1*15:HLA-DRB_*5*01:HLA-DQ*05:HLA-DQ*06
8	HLA-A*0201:HLA-A*01:HLA-A*02:HLA-B*08:HLA-B*1402:HLA-Cw*07:HLA-Cw*08:HLA-DRB1*03:HLA-DRB1*11:HLA-DRB_*3*01:HLA-DRB_*3*02:HLA-DQ*02:HLA-DQ*03
10	HLA-A*0201:HLA-A*24:HLA-B*13:HLA-B*15:HLA-Cw*03:HLA-Cw*06:HLA-DRB_*4*01:HLA-DRB1*04:HLA-DRB1*07:HLA-DQ*02:HLA-DQ*0302
12	HLA-A*01:HLA-A*02:HLA-A*0201:HLA-B*08:HLA-B*40:HLA-Cw*03:HLA-Cw*07:HLA-DRB1*03:HLA-DRB1*13:HLA-DRB_*3*0101:3*0301:HLA-DQ*02:HLA-DQ*06
13	HLA-A*01:HLA-A*68:HLA-B*49:HLA-B*57:HLA-Cw*7:HLA-DRB1*03:HLA-DRB_*3*00:HLA-DRB1*11:HLA-DQ*03:HLA-DQ*04
14	HLA-A*11:HLA-A*68:HLA-B*14:HLA-B*35:HLA-Cw*04:HLA-Cw*08:HLA-DRB1*01:HLA-DRB1*13:HLA-DRB_*3*0101:HLA-DQ*03:HLA-DQ*05
15	HLA-A*02:HLA-A*03:HLA-B*14:HLA-B*51:HLA-Cw*02:HLA-Cw*08:HLA-DRB_*3*0301:4*01:HLA-DRB1*04:HLA-DRB1*13:HLA-DQ*03:HLA-DQ*06
17	HLA-A*01:HLA-A*29:HLA-B*08:HLA-B*44:HLA-Cw*07:HLA-Cw*16:HLA-DRB_*3*02:HLA-DRB1*08:HLA-DRB1*14:HLA-DQ*0402:HLA-DQ*0503
18	HLA-A*03:HLA-A*24:HLA-B*08:HLA-B*15:HLA-Cw*07:DQB1*02:01:HLA-DRB1*05:01:DRB1*01:HLA-DRB1*03:DRB_*3*01:01
19	HLA-A*02:HLA-A*31:HLA-B*35:HLA-B*37:HLA-Cw*04:HLA-Cw*06:DQB1*5:DRB1*1
24	HLA-A*02:HLA-A*11:HLA-B*35:HLA-B*45:HLA-Cw*04:HLA-Cw*06:HLA-DRB1*11:HLA-DRB1*14:HLA-DQ*03:HLA-DQ*05

UPN, unique patient number; F, female; M, male; 3M/6M, 3 months/6 months; CMV, cytomegalovirus; CsA, cyclosporine; MTX, methotrexate; CTX, cyclophosphamide; ATG, anti-thymocyte globulin; CR, complete response; PR, partial response; NR, non-response; NE, not evaluable.

Supplementary Table 2 α and β chains detected in single cells.

ID	UPN/ HD	Samples	All cells	Cells with α chain	Cell with β chain	Cells with both chanins	Cells with one α chain	Cells with two α chains	Cells with three α chains	Cells with one β chain	Cells with two β chains	Cells with three β chains	Cells with one α and one β chains
**Sample 1**	UPN24	Pre	7572	5237	7247	4912	4955	275	7	6761	462	23	4337
**Sample 2**	UPN24	3M_CR	11210	4907	10934	4631	4760	142	5	9602	1226	95	3780
**Sample 3**	UPN10	Pre	7995	5106	7726	4837	4923	179	4	7346	364	15	4412
**Sample 4**	UPN10	3M_CR	25761	15140	25136	14515	14279	802	53	20471	4104	508	10589
**Sample 5**	UPN19	Pre	18918	14897	18081	14060	13223	1495	166	15484	2305	274	10905
**Sample 6**	UPN19	3M_PR	20316	14873	19345	13902	13433	1299	129	16179	2788	336	10485
**Sample 7**	HD1	HD1	14888	9542	14324	8978	9064	446	30	12440	1729	141	7242
**Sample 8**	HD2	HD2	19192	9318	17158	7284	9265	50	3	16624	488	45	6944
**Sample 9**	UPN18	Pre	22489	10214	21519	9244	10017	193	4	17604	3380	482	7023
**Sample 10**	UPN18	3M_PR	32121	21336	30132	19347	18888	2106	310	21097	6988	1707	11444
**Sample 11**	UPN4	Pre	62055	46906	59706	44557	44914	1886	99	46241	11615	1686	32492
**Sample 12**	UPN4	3M_PR	23699	20216	23167	19684	19079	1046	87	21245	1812	106	17398
**Sample 13**	UPN17	Pre	32083	23805	29773	21495	20890	2502	359	22625	5850	1138	14041
**Sample 14**	UPN17	6M_NR	928	428	873	378	398	30	0	863	10	0	347
**Sample 15**	HD3	HD3	10147	5783	9722	5358	5596	182	5	8807	859	51	4673
**Sample 16**	HD4	HD4	9055	5933	8673	5551	5663	262	8	8027	605	40	4887
**Sample 17**	UPN1	Pre	8013	6265	7311	5563	5873	367	25	6865	419	27	4924
**Sample 18**	UPN1	3M_CR	11748	9737	11298	9287	8658	1018	54	10265	962	69	7693
**Sample 19**	UPN12	Pre	8474	5842	7721	5089	5394	430	18	7236	456	29	4398
**Sample 20**	UPN12	3M_CR	10783	9638	10434	9289	8042	1452	133	9474	897	61	7285
**Sample 21**	UPN8	Pre	4402	1985	3993	1576	1962	23	0	3886	106	1	1501
**Sample 22**	UPN13	Pre	7091	3924	6415	3248	3849	75	0	6109	292	14	3019
**Sample 23**	HD5	HD5	46781	35816	44590	33625	31080	4000	636	28239	11857	3602	18063
**Sample 24**	HD6	HD6	2374	1125	2118	873	1092	33	0	2067	51	0	828
**Sample 25**	UPN8	6M_CR	2404	835	2282	713	828	7	0	2224	57	1	692
**Sample 26**	UPN13	6M_PR	2720	1177	2549	1006	1170	7	0	2481	68	0	976
**Sample 27**	UPN14	Pre	3700	2088	3392	1781	2056	32	0	3309	81	2	1711
**Sample 28**	UPN14	6M_NR	3599	2359	3261	2024	2313	46	0	3158	99	4	1930
**Sample 29**	UPN15	Pre	5783	2461	5546	2224	2455	6	0	5367	176	3	2150
**Sample 30**	UPN15	6M_NR	6837	3995	6561	3719	3982	13	0	6517	44	0	3685
**Sample 31**	UPN6	Pre	1146	309	906	70	309	0	0	904	2	0	69
**Sample 32**	HD7	HD7	2600	1121	2395	916	1115	5	1	2320	73	2	892

UPN, unique patient number; HD, healthy donor; Pre, pre-treatment; 3M/6M, 3 months/6 months; CR, complete response; PR, partial response.

Supplementary Table 3 TCR specificity groups defined by GLIPH analysis.

Rank	Convergence group (CRG)	CRG contains clones	Frequency in all cells
**1**	CRG-CASSPGTNYGYTF_size_21	21	9053
**2**	CRG-CASIVGSYNEQFF_size_238	238	2633
**3**	CRG-CASRAGETEAFF_size_237	237	1750
**4**	CRG-CASSFEETQYF_size_242	242	1699
**5**	CRG-CASSLVGGSYEQYF_size_61	61	560
**6**	CRG-CASRARGGNQPQHF_size_37	37	465
**7**	CRG-CASSLTSTDTQYF_size_43	43	334
**8**	CRG-CASSPGFSYEQYF_size_46	46	263
**9**	CRG-CASSLGHNTDTQYF_size_43	43	201
**10**	CRG-CASSLDWETQYF_size_35	35	173
**11**	CRG-CASSLAGNTGELFF_size_33	33	171
**12**	CRG-CASSLAGYAYNEQFF_size_42	42	150
**13**	CRG-CASSIQGNQPQHF_size_26	26	119
**14**	CRG-CATDTGDSNQPQHF_size_10	10	103
**15**	CRG-CASSLYNQPQHF_size_19	19	93
**16**	CRG-CASSPDNYGYTF_size_18	18	92
**17**	CRG-CASSPTGWETQYF_size_15	15	90
**18**	CRG-CASSENYSNQPQHF_size_6	6	79
**19**	CRG-CASSLGTVNTGELFF_size_13	13	72
**20**	CRG-CASSLTAGSSYEQYF_size_9	9	72
**21**	CRG-CASSDYEQYF_size_10	10	68
**22**	CRG-CASSLLSSYNEQFF_size_14	14	68
**23**	CRG-CASSLGLQETQYF_size_16	16	67
**24**	CRG-CAISESGSSYEQYF_size_9	9	64
**25**	CRG-CASRRDSSYEQYF_size_14	14	54
**26**	CRG-CASSFAGMNTEAFF_size_12	12	51
**27**	CRG-CASSPPSGVTDTQYF_size_6	6	49
**28**	CRG-CASRTGSTGELFF_size_11	11	43
**29**	CRG-CASSPLGSSYNEQFF_size_12	12	39
**30**	CRG-CASAPGLAGGEQFF_size_6	6	38
**31**	CRG-CASSIGTAYNEQFF_size_6	6	38
**32**	CRG-CASSLGGYSNQPQHF_size_7	7	38
**33**	CRG-CASSPPQGNTEAFF_size_9	9	38
**34**	CRG-CASSRDSNYGYTF_size_9	9	38
**35**	CRG-CASSSDSGGTDTQYF_size_6	6	38
**36**	CRG-CASSSQAGGYNEQFF_size_7	7	37
**37**	CRG-CASSLGLAGYNEQFF_size_8	8	34
**38**	CRG-CSGGRLNTEAFF_size_16	16	34
**39**	CRG-CSASFNEQFF_size_6	6	33
**40**	CRG-CSVDGSSYEQYF_size_10	10	33
**41**	CRG-CAISEGGEQETQYF_size_6	6	32
**42**	CRG-CASTYSGANVLTF_size_8	8	25
**43**	CRG-CASSLAQGSETQYF_size_6	6	24
**44**	CRG-CASRGDGYEQYF_size_7	7	23
**45**	CRG-CASSLDTSPLHF_size_6	6	21
**46**	CRG-CASGPNTIYF_size_6	6	20
**47**	CRG-CASSSGLAGTDTQYF_size_6	6	18
**48**	CRG-CASRRGGEQYF_size_8	8	15
**49**	CRG-CASRGGGETQYF_size_6	6	7

GLIPH, Grouping of Lymphocyte Interactions by Paratope Hotspots.

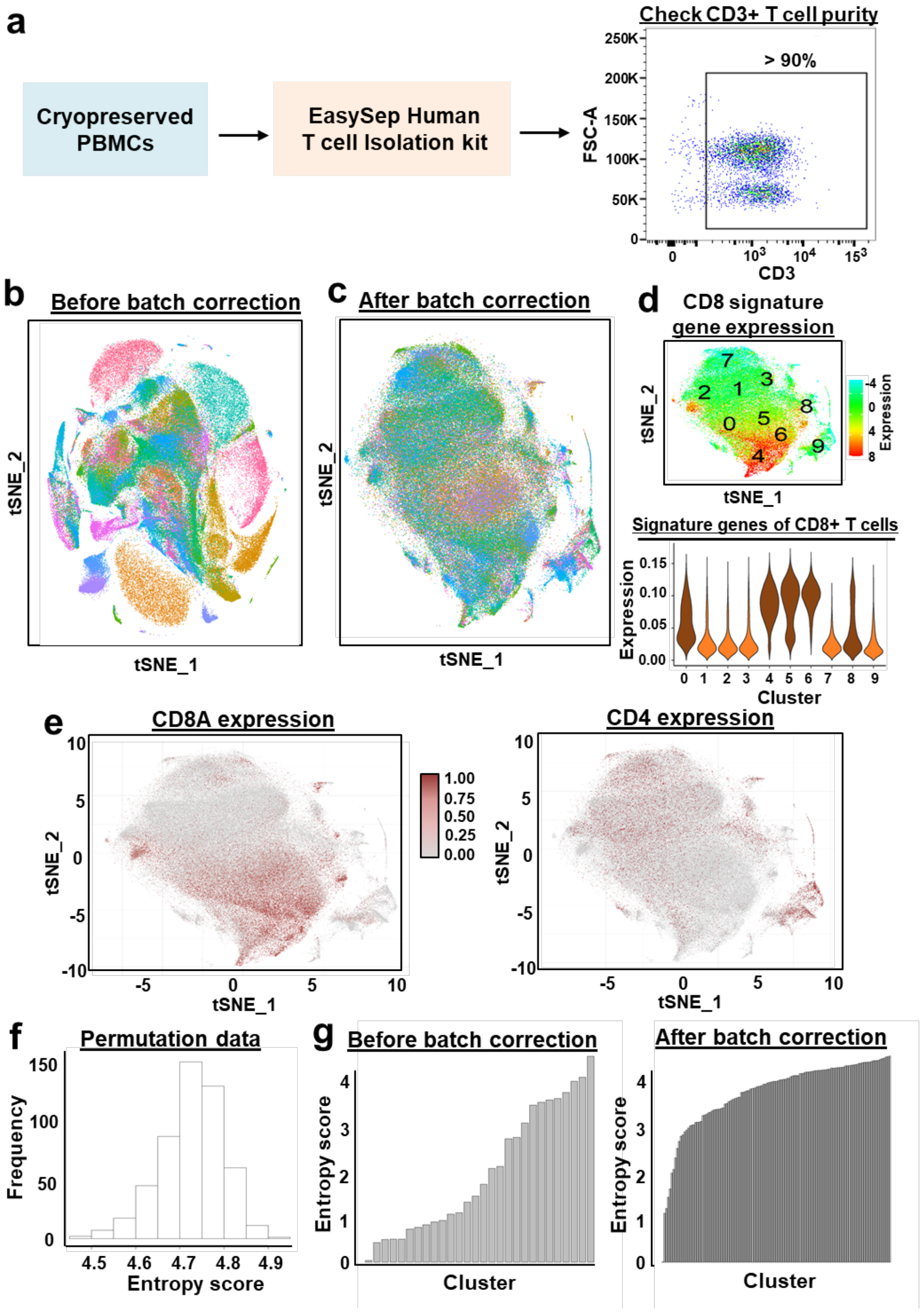

Supplementary Fig. 1 Data merging and removal of batch effects. a Cryopreserved PBMCs were used to enrich T cells using the EasySep Human T cell Isolation kit, with purity (detected by flow cytometry staining with anti-human CD3) after enrichment > 90%. **b** t-SNE plots by expression data before batch correction (colored by samples). **c** t-SNE plots by expression data after batch correction (colored by samples). **d** Expression of CD8 signature genes of metaclusters identified by PhonoGraph algorithm. **e** The same t-SNE plot shown in Fig. **1c**, colored by CD8A and CD4 expression. **f** A histogram plot of entropy scores of sample distribution on permutation data, with entropy scores on x-axis and frequency on y-axis. **g** Entropy scores of sample distribution (y-axis) of clusters (x-axis) and before (left) and after (right) batch corrections.

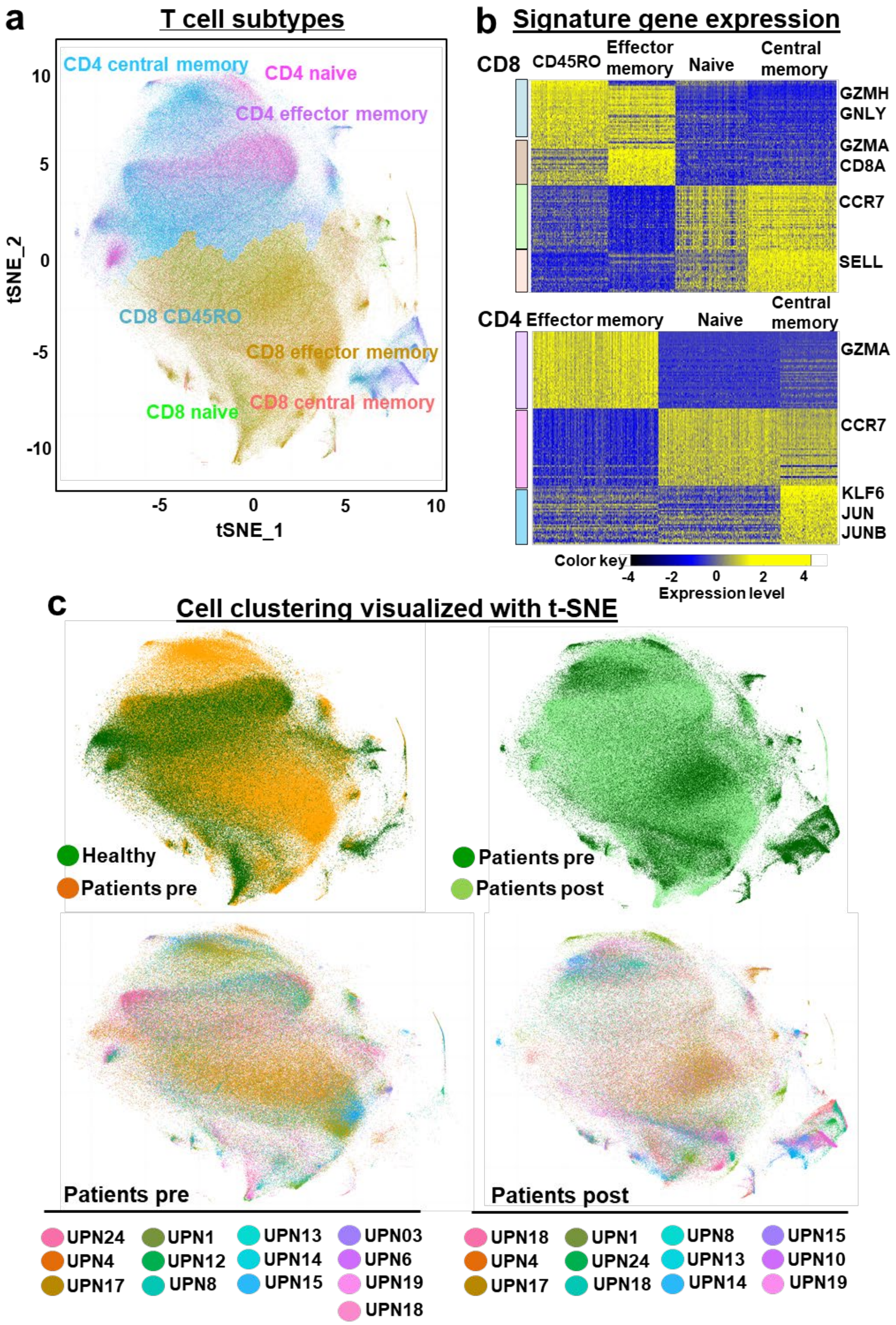

Supplementary Fig. 2 Characterization of T cell subsets. a The same t-SNE plot shown in Fig. **1b**, colored by T cell subtypes defined by calculating the area under the curve (AUC) scores with GSE93777 for each cell. **b** A heatmap showing expression of signature genes of each T cell subtypes. **c** The same t-SNE plot shown in Fig. **1b**, colored by patients and healthy donors; patients pre- and post-treatments; individual patients' samples pre- and post-treatments, respectively.

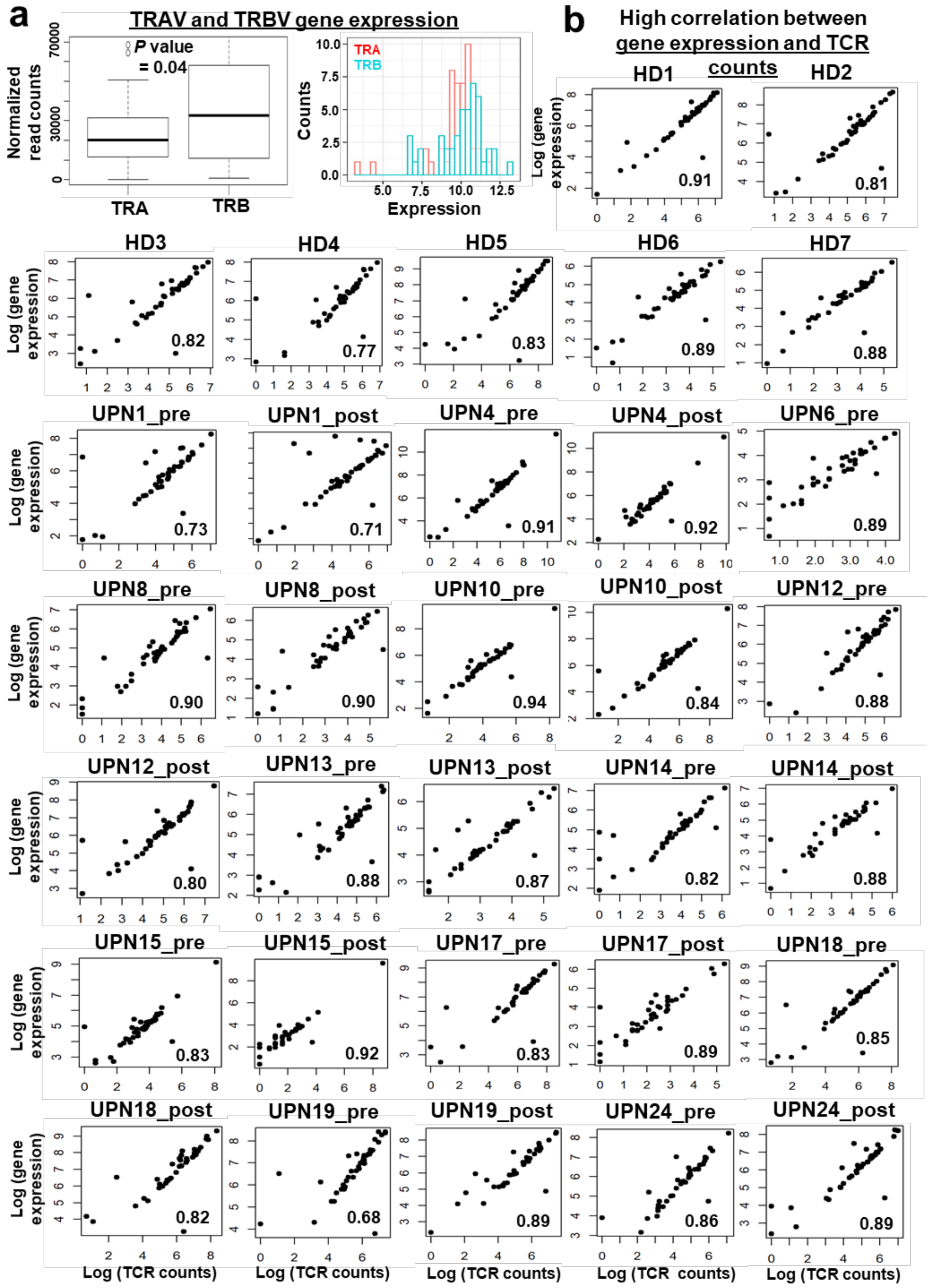

Supplementary Fig. 3 Expression of *TRAV* and *TRBV* genes in scRNA-seq. a Shown are 25% to 75% response ranges (top and bottom lines of boxes), and minima and maxima (bars). A two-sided unpaired t-test. *P* value was indicated in the figure. **b** A high correlation between counts of captured unique CDRs by scTCR-seq and expression levels of TRVB genes that encoded corresponding CDRs of TCR in scRNA-seq. TCR counts in Log on x-axis and expression of corresponding TRVB genes in Log on y-axis.

Vβ/Vα and matching Jβ/Jα

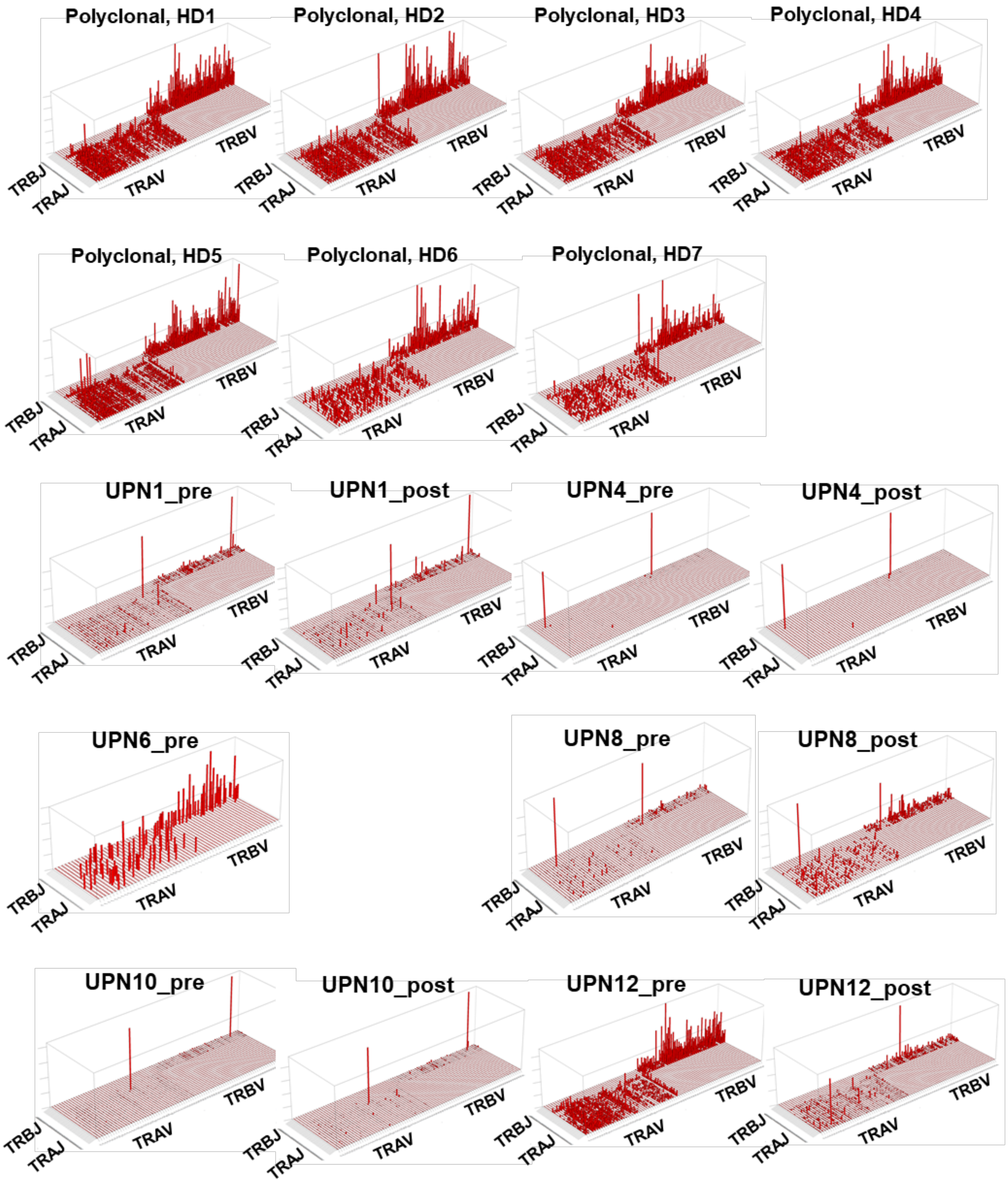

Supplementary Fig. 4 Skyscraper plots showing Vβ/Vα and matching Jβ/Jα in healthy donors and patients. Skyscraper plots show Vβ/Vα and matching Jβ/Jα in healthy donors (HD1 - HD7) and patients (UPNs 1, 4, 6, 8, 10 and 12,) pre- and post-alemtuzumab treatments. A UPN6 post-treatment sample was not available.

Vβ/Vα and matching Jβ/Jα

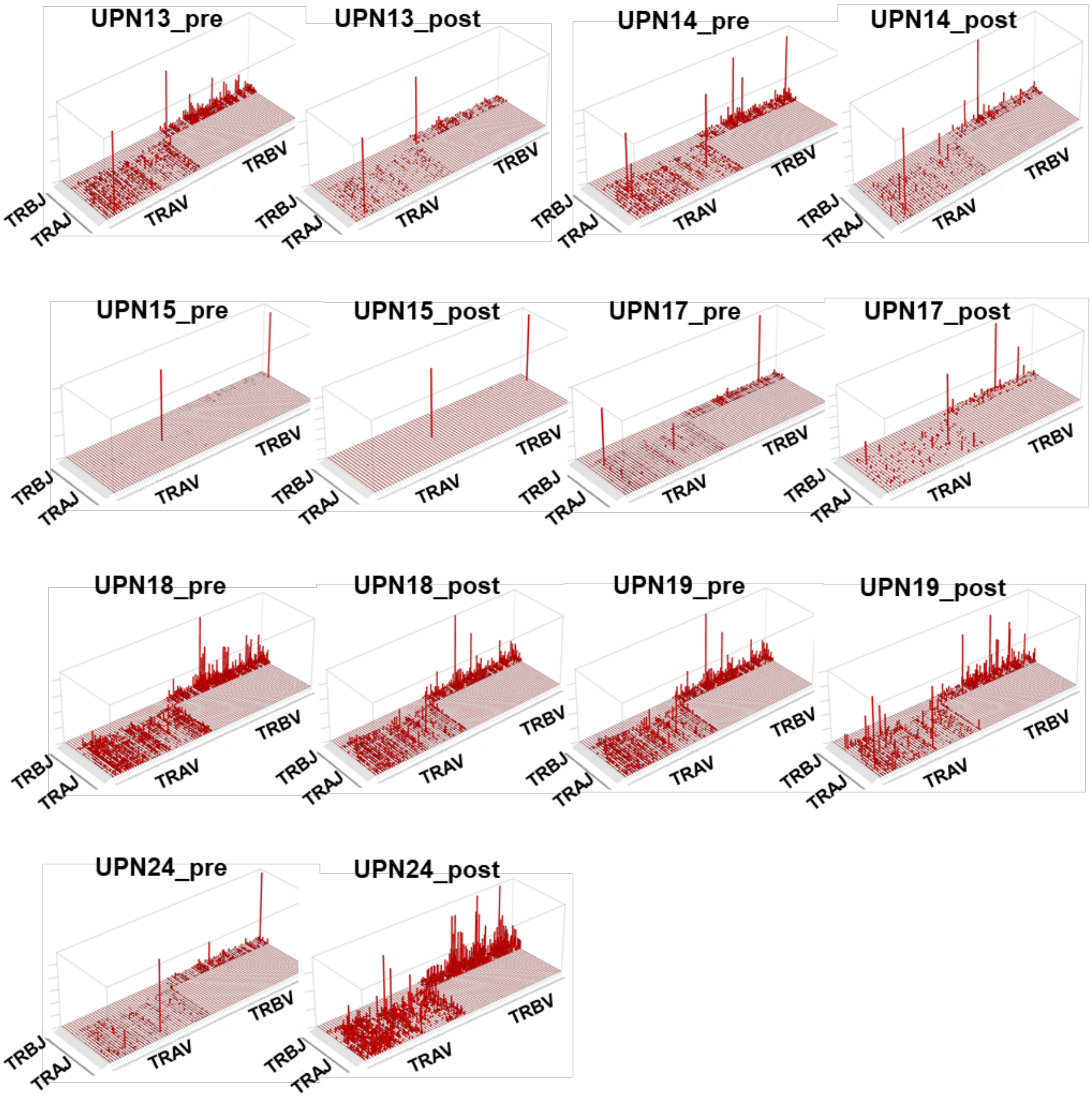

Supplementary Fig. 5 Skyscraper plots showing Vβ/Vα and matching Jβ/Jα in patients. Skyscraper plots show Vβ/Vα and matching Jβ/Jα in patients (UPNs 13, 14, 15, 17, 18, 19 and 24) pre- and post-alemtuzumab treatments.

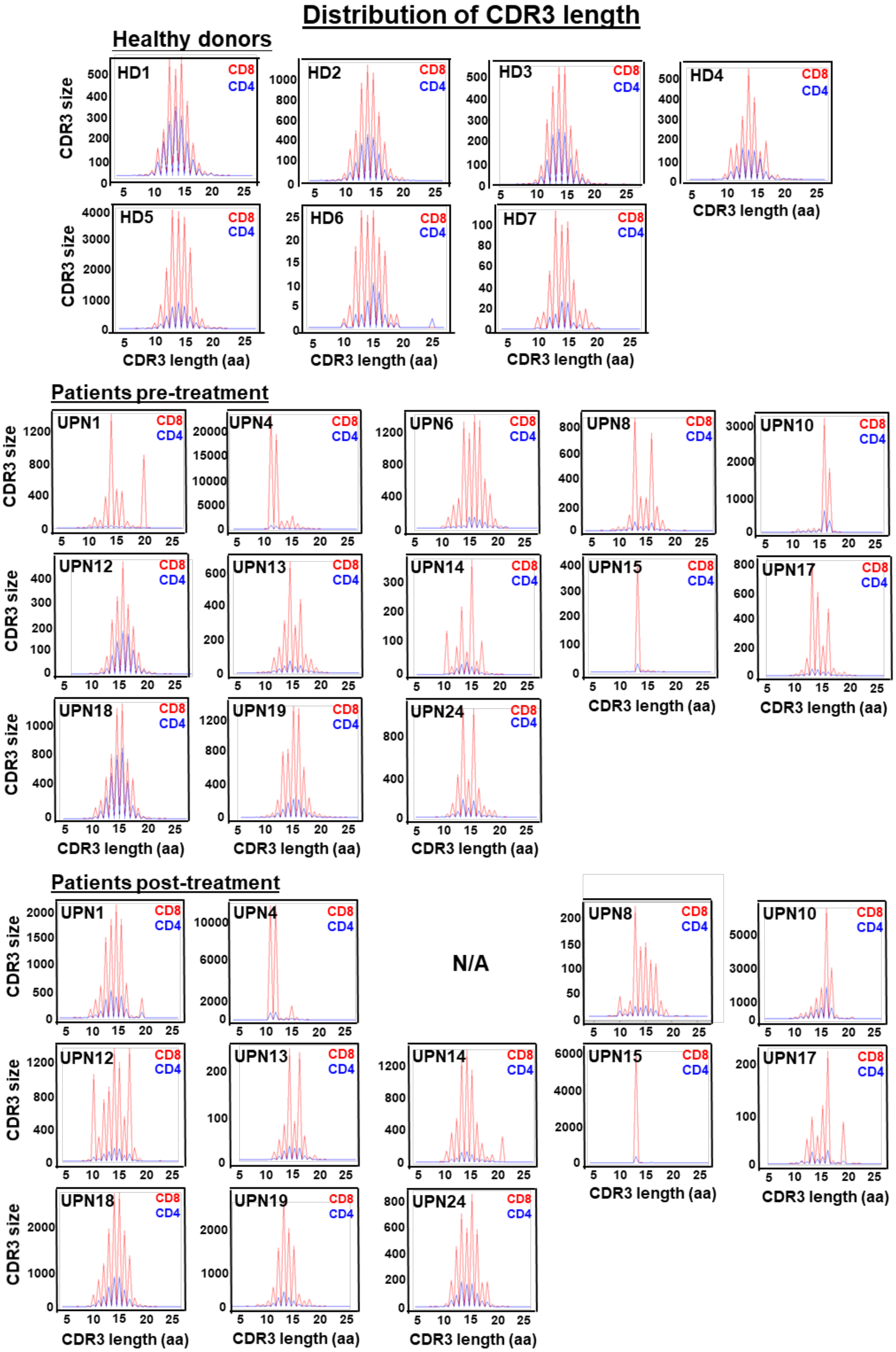

Supplementary Fig. 6 Distribution of CDR3 lengths in healthy donors and patients. Red and blue curves indicate CD8^{+} and CD4^{+} T cells, respectively. x-axis, CDR3 length in amino acid (aa); y-axis, CDR3 size in cell counts.

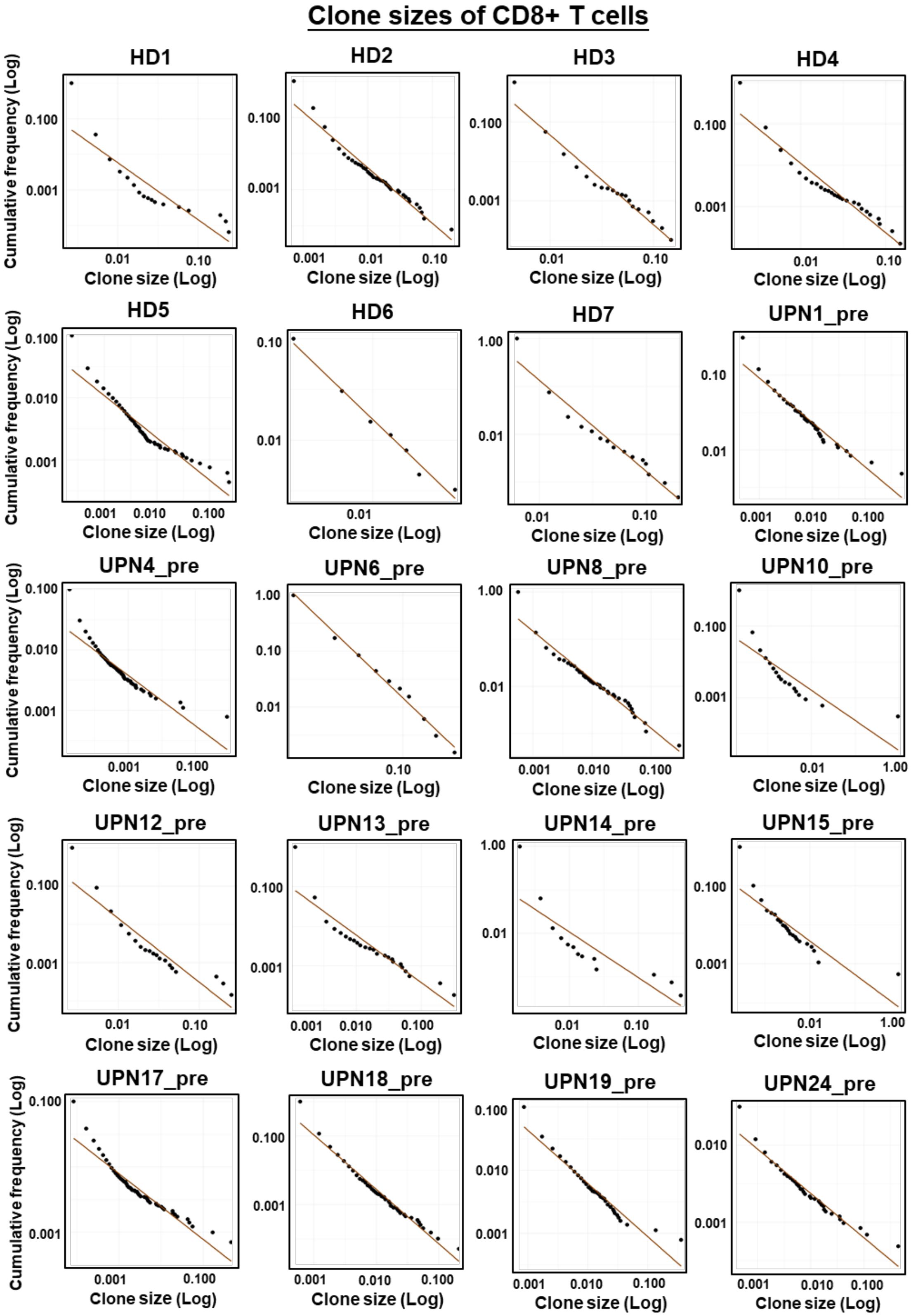

Supplementary Fig. 7 Clone sizes in CD8⁺ T cells of healthy donors and patients. Clone sizes were plotted in $CD8^+$ T cells of healthy donors (HD1 – HD7) and patients (UPNs 1, 4, 6, 8, 10, 12, 13, 14, 15, 17, 18, 19 and 24) pre-treatment with clone sizes in Log on x-axis and Log of cumulative frequency on y-axis.

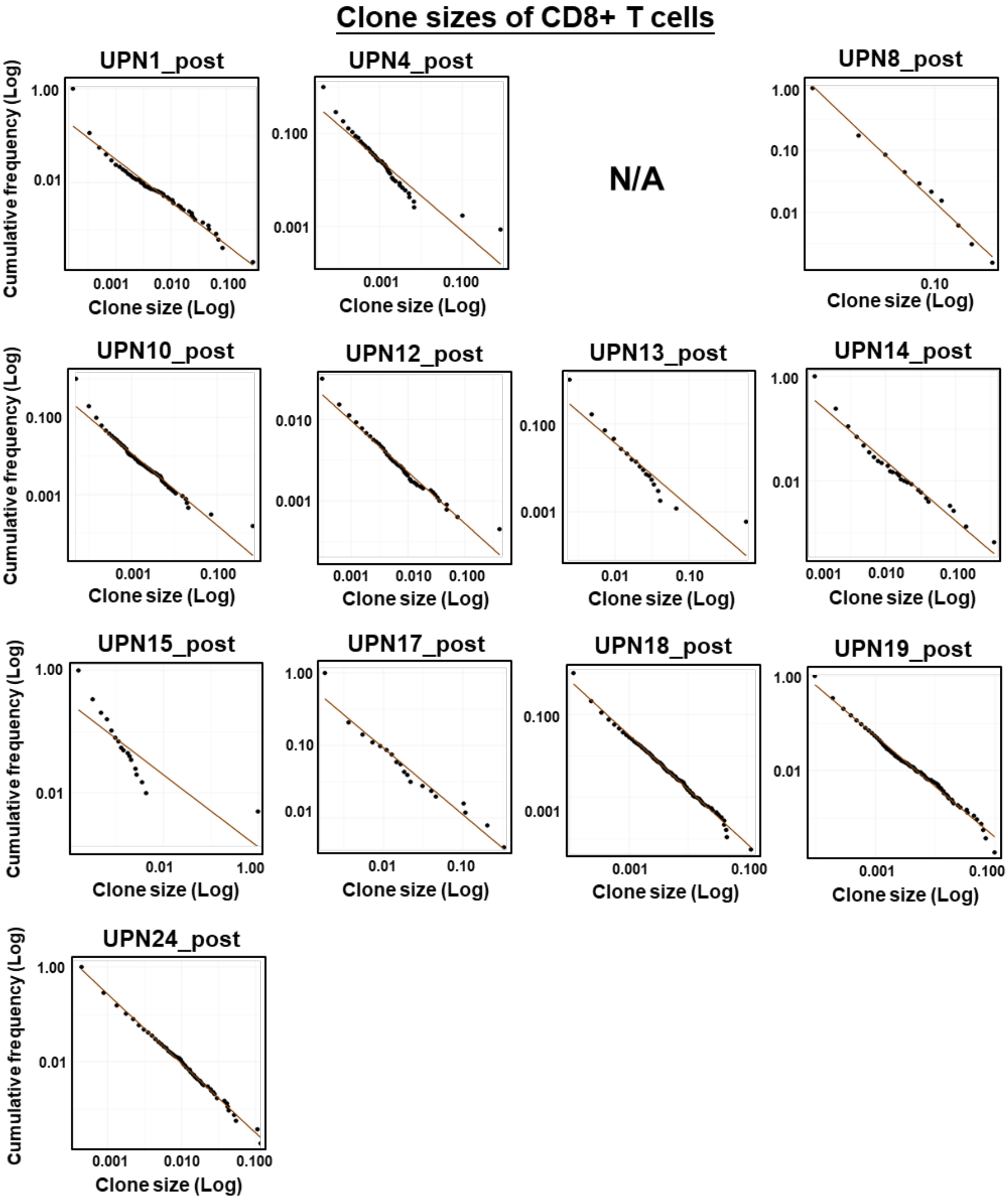

Supplementary Fig. 8 Clone sizes in CD8$^+$ T cells of patients. Clone sizes were plotted in CD8$^+$ T cells of patients (UPNs 1, 4, 8, 10, 12, 13, 14, 15, 17, 18, 19 and 24) post-alemtuzumab treatment with clone sizes in Log on x-axis and Log of cumulative frequency on y-axis. A UPN6 post-treatment sample was not available.

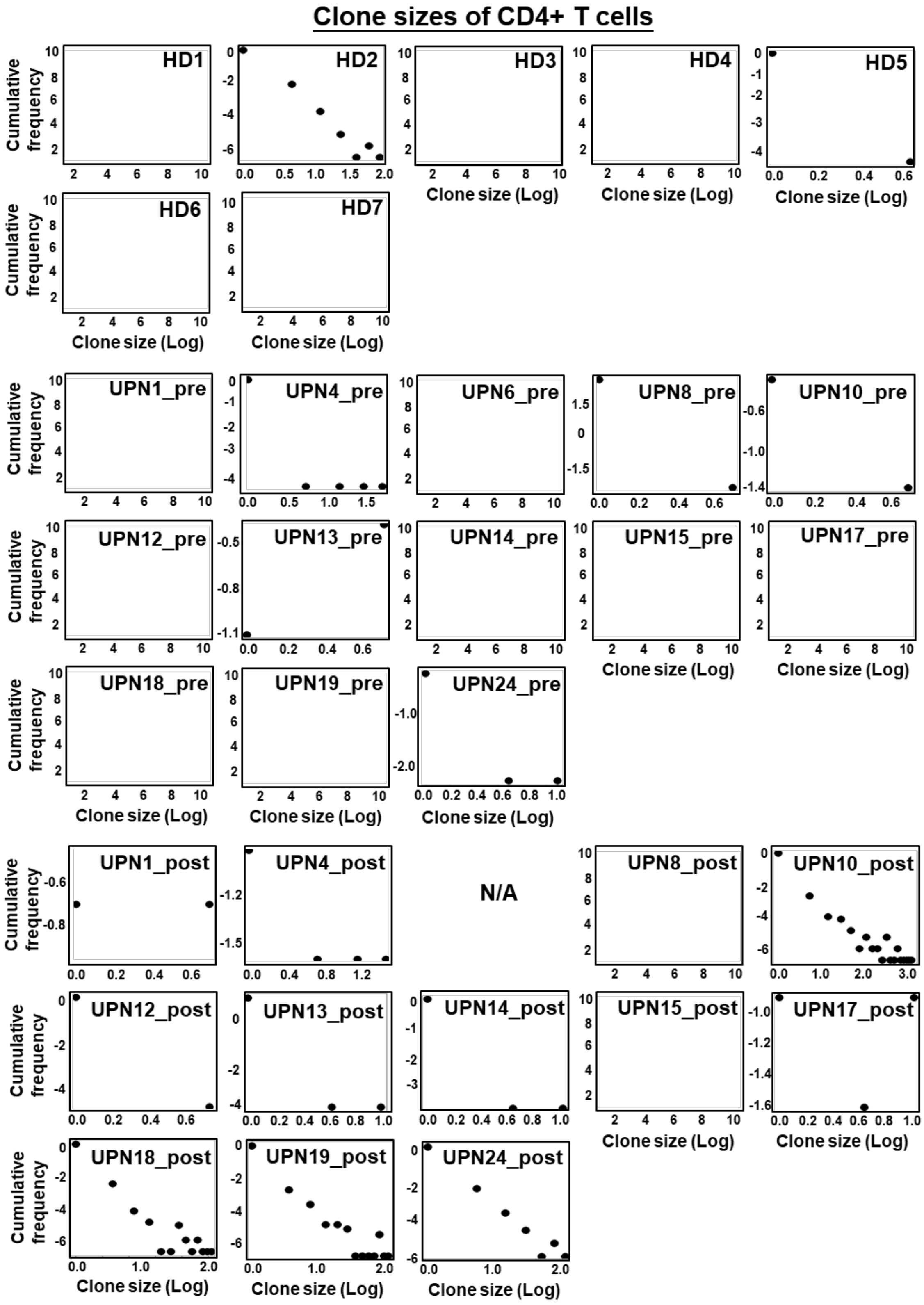

Supplementary Fig. 9 Clone sizes in CD4$^+$ T cells of healthy donors and patients. Clone sizes were plotted in CD4$^+$ T cells of healthy donors (HD1 – HD7) and patients (UPNs 1, 4, 6, 8, 10, 12, 13, 14, 15, 17, 18, 19 and 24) pre- and post-alemtuzumab treatments with clone sizes on Log on x-axis and Log of cumulative frequency on y-axis. A UPN6 post-treatment sample was not available.

Homology assessment: top 500 TCR clones

		Patients (UPN)																								Healthy (HD)						
		1		4		6	8		10		12		13		14		15		17		18		19		24		1	2	3	4	5	6	7
		pre	post	pre	post	pre	pre	post	pre	post	pre	post	pre	post	pre	post	pre	post	pre	post	pre	post	pre	post	pre	post							
Patients (UPN) 1	pre	500	75	0	0	0	0	0	0	0	1	2	0	0	0	0	0	0	1	0	0	0	1	0	0	1	0	1	1	0	0	0	0
	post	75	500	0	2	0	0	0	0	0	0	0	0	0	0	0	0	0	1	0	1	1	0	0	0	0	1	0	0	1	0	0	0
4	pre	0	0	500	110	0	0	0	0	0	2	1	0	1	0	0	0	0	0	0	0	1	1	1	0	1	1	1	1	0	0	0	0
	post	0	2	110	500	10	0	0	0	0	1	1	1	0	0	1	1	0	0	0	0	1	1	0	0	0	1	0	1	0	0	0	0
6	pre	0	0	0	10	500	1	1	0	0	0	1	0	0	0	1	0	0	0	0	1	0	0	0	0	0	1	0	0	0	0	0	0
8	pre	0	0	0	0	1	500	85	0	0	1	1	0	0	0	0	0	0	1	0	0	0	0	0	2	0	1	0	0	2	0	0	0
	post	0	0	0	0	1	85	500	1	0	1	1	0	0	0	0	0	0	2	0	0	0	0	0	0	0	2	0	1	0	1	0	0
10	pre	0	0	0	0	0	0	1	500	90	1	0	0	0	2	0	0	0	0	0	1	0	1	0	0	0	1	0	1	0	1	0	1
	post	0	0	0	0	0	0	0	90	500	0	1	0	1	0	0	0	0	0	0	0	0	0	0	0	0	0	0	0	0	0	0	0
12	pre	1	0	2	1	0	1	1	1	0	500	55	2	0	0	0	0	0	0	1	2	1	1	0	0	1	1	0	1	1	1	1	0
	post	2	0	1	1	1	1	1	0	1	55	500	1	1	1	0	0	0	0	1	0	0	0	0	1	2	0	0	1	0	1	0	0
13	pre	0	0	0	1	0	0	0	0	0	2	1	500	21	0	0	0	0	1	1	0	0	0	1	1	0	0	0	0	1	0	1	0
	post	0	0	1	0	0	0	0	0	1	0	1	21	500	0	0	0	0	0	0	0	0	0	0	0	0	1	0	0	1	0	0	1
14	pre	0	0	0	0	0	0	0	2	0	0	1	0	0	500	13	0	0	0	0	0	0	2	0	0	0	0	0	0	0	0	0	0
	post	0	0	0	1	1	0	0	0	0	0	0	0	0	13	500	0	0	1	1	1	1	1	0	2	0	0	0	0	0	0	0	0
15	pre	0	0	0	0	0	0	0	0	0	0	0	0	0	0	0	500	37	0	0	1	0	0	0	0	1	0	0	0	1	0	0	0
	post	0	0	0	1	0	0	0	0	0	0	0	0	0	0	0	37	500	0	1	0	0	0	0	0	0	0	0	0	0	1	0	0
17	pre	1	1	0	0	0	1	2	0	0	0	0	1	0	0	1	0	0	500	43	1	1	0	0	0	0	0	0	0	1	0	1	0
	post	0	0	0	0	0	0	0	0	0	1	1	1	0	0	1	1	0	43	500	0	0	1	0	0	1	0	0	0	0	1	0	0
18	pre	0	1	0	0	1	0	0	1	0	2	0	0	0	0	1	0	1	1	0	500	164	2	0	0	0	1	1	0	0	0	1	0
	post	0	1	1	1	0	0	0	0	0	1	0	0	0	0	1	0	0	1	0	164	500	0	1	0	0	1	1	1	2	0	1	0
19	pre	1	0	1	1	0	0	0	1	0	1	0	0	0	2	1	0	0	0	1	2	0	500	24	1	0	2	0	0	0	2	0	1
	post	0	0	1	0	0	0	0	0	0	0	0	1	0	0	0	0	0	0	0	0	1	24	500	0	0	1	1	1	0	0	0	0
24	pre	0	0	0	0	0	2	0	0	0	0	1	1	0	0	2	0	0	0	0	0	0	1	0	500	126	0	0	1	1	0	0	0
	post	1	0	1	0	0	0	0	0	0	1	2	0	0	0	0	0	1	0	1	0	0	0	0	126	500	0	0	1	0	0	0	0
Healthy (HD)	1	0	1	1	1	1	1	2	1	0	1	0	0	1	0	0	0	0	0	0	1	1	2	1	0	0	500	2	4	1	1	0	2
	2	1	0	1	0	0	0	0	0	0	0	0	0	0	0	0	0	0	0	0	1	1	0	1	0	0	2	500	1	1	1	0	0
	3	1	0	1	1	0	0	1	1	0	1	1	0	0	0	0	0	0	0	0	0	1	0	1	1	1	4	1	500	1	1	0	0
	4	0	1	0	0	0	2	0	0	0	1	0	1	1	0	0	0	1	1	0	0	2	0	0	1	0	1	1	1	500	0	1	0
	5	0	0	0	0	0	0	1	1	0	1	1	0	0	0	0	1	0	0	1	0	0	2	0	0	0	1	1	1	0	500	0	0
	6	0	0	0	0	0	0	0	0	0	1	0	1	0	0	0	0	0	1	0	1	1	0	0	0	0	0	0	0	1	0	500	0
	7	0	0	0	0	0	0	0	1	0	0	0	0	1	0	0	0	0	0	0	0	0	1	0	0	0	2	0	0	0	0	0	500

Number of Shared TCR sequences: <5 | 5 - 10 | 10 - 20 | 20 - 50 | >50

Supplementary Fig. 10 Lack of common T cell clonotypes was seen in T-LGLL patients in our study: top 500 TCR clones. A heatmap plot showing sharing among top 500 TCR clones of patients and healthy donors. On both x- and y-axes, there were samples of patients and healthy donors, and paired samples of the same patients were adjacent. Numbers indicate counts of identical TCR clones shared among samples. A color scheme (dark orange to dark blue) indicates the number of shared CDR sequences from high to low. Although by increasing the number of clones examined and increasing resolution, there were more clones shared among samples, majority of clones were only shared in the same patient before and after treatments, and only found at basal levels similarly in other patients and in healthy donors.

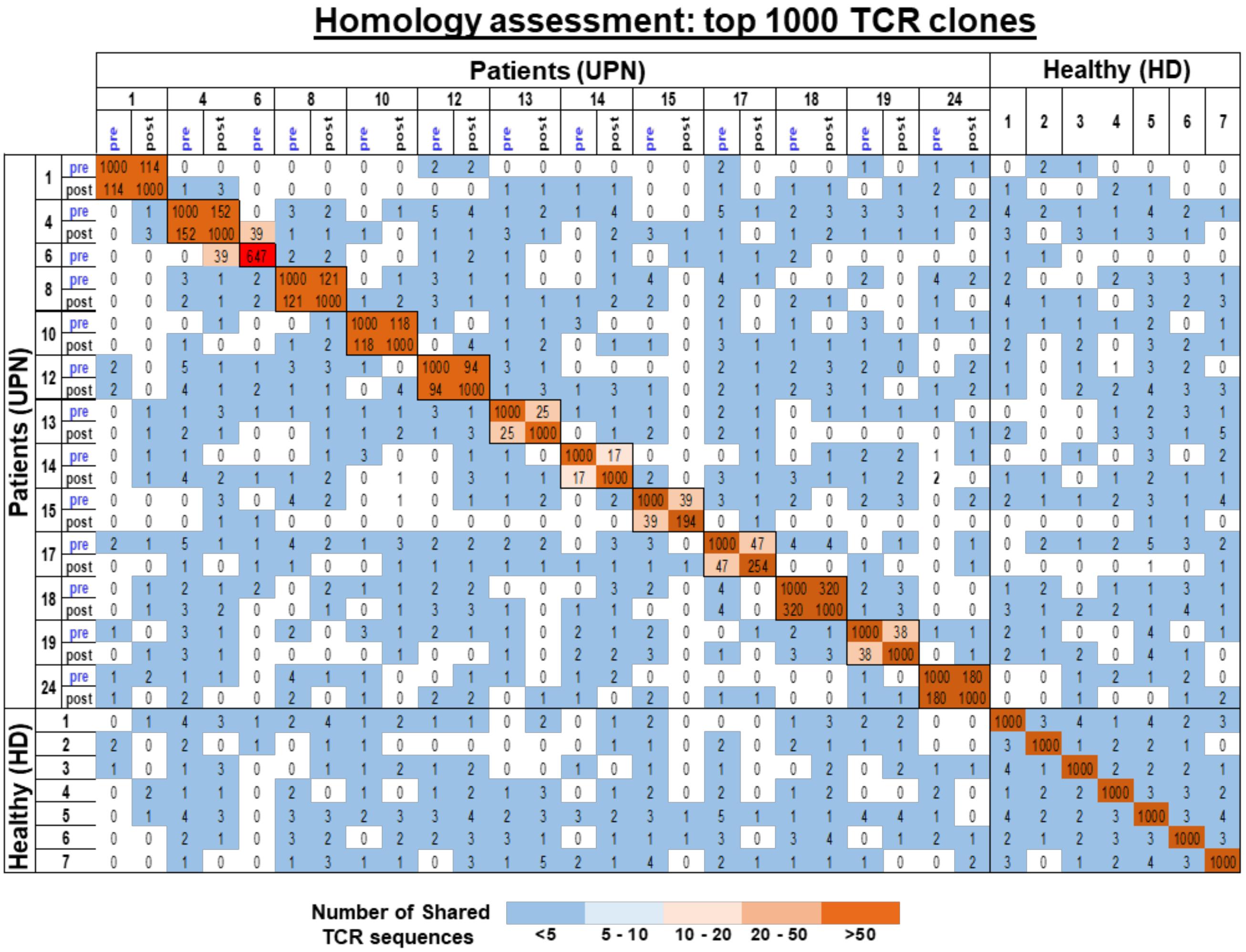

Supplementary Fig. 11 Lack of common T cell clonotypes was also seen in T-LGLL patients in our study: top 1000 TCR clones. A heatmap plot showing sharing among top 1000 TCR clones of patients and healthy donors. On both x- and y-axes, there were samples of patients and healthy donors, and paired samples of the same patients were adjacent. Numbers indicate counts of identical TCR clones shared among samples. A color scheme (dark orange to dark blue) indicates the number of shared CDR sequences from high to low. Although by increasing the number of clones examined and increasing resolution, there were more clones shared among samples, majority of clones were only shared in the same patient before and after treatments, and only found at basal levels similarly in other patients and in healthy donors.

a **Homology assessment: overlapping with clones in two references**

TCR in our data	Sample	Cells No.	Cells (%)	TCR in literature	Literature
CASSQGRGSGGNTIYF	UPN24_post	6	0.06555944	CASSQGRG	Clemente MJ, et al. Blood.2013
CASSQGRGANTGELFF	UPN10_post	2	0.007705644	CASSQGRG	Clemente MJ, et al. Blood.2013
CASSQGRGDSDNEQFF	UPN18_pre	2	0.02385781	CASSQGRG	Clemente MJ, et al. Blood.2013
CASSQGRGLGSPLHF	UPN18_post	6	0.0254205	CASSQGRG	Clemente MJ, et al. Blood.2013
CASSQGRGQGAGETQYF	UPN4_pre	2	0.003275574	CASSQGRG	Clemente MJ, et al. Blood.2013
CASSQGRGSQETQYF	UPN4_pre	2	0.003275574	CASSQGRG	Clemente MJ, et al. Blood.2013
CASSQGRGQGAGETQYF	UPN4_post	1	0.003967624	CASSQGRG	Clemente MJ, et al. Blood.2013
CASSQGRGDEQFF	UPN17_pre	3	0.0153335	CASSQGRG	Clemente MJ, et al. Blood.2013
CASSQGRGPSQPQHF	UPN12_post	2	0.0232369	CASSQGRG	Clemente MJ, et al. Blood.2013
CASSQGRGGQPQHF	UPN8_pre	1	0.0243843	CASSQGRG	Clemente MJ, et al. Blood.2013
CASSQGRGSQNTEAFF	UPN8_pre	1	0.0243843	CASSQGRG	Clemente MJ, et al. Blood.2013
CASSQGRGLSYEQYF	UPN13_post	1	0.03821169	CASSQGRG	Clemente MJ, et al. Blood.2013
CASSQGRGSSTDTQYF	UPN14_pre	1	0.03644315	CASSQGRG	Clemente MJ, et al. Blood.2013
CASSQGRGSGEQYF	UPN14_post	1	0.02969121	CASSQGRG	Clemente MJ, et al. Blood.2013
CASSQGRGGPYNEQFF	UPN15_pre	2	0.0349162	CASSQGRG	Clemente MJ, et al. Blood.2013
CASSQGRGPGYEQYF	UPN15_pre	1	0.0174581	CASSQGRG	Clemente MJ, et al. Blood.2013
CASSQGRGRVRNEQFF	HD1	4	0.1255887	CASSQGRG	Clemente MJ, et al. Blood.2013
CASSQGRGVNYGYTF	HD5	19	0.1346945	CASSQGRG	Clemente MJ, et al. Blood.2013
CASSQGRGGSTYEQYF	HD5	3	0.02126755	CASSQGRG	Clemente MJ, et al. Blood.2013
CASSQGRGTEAFF	HD5	3	0.02126755	CASSQGRG	Clemente MJ, et al. Blood.2013
CASSQGRGDEQFF	HD6	1	0.0461042	CASSQGRG	Clemente MJ, et al. Blood.2013
CASSQGRGLETQYF	HD6	1	0.0461042	CASSQGRG	Clemente MJ, et al. Blood.2013
CASSLGGQPQHF	UPN18_post	8	0.033894	ASSLGGQPQH	Clemente MJ, et al. Blood. 2011

b **Share TCR usage in the Blood Advances paper**

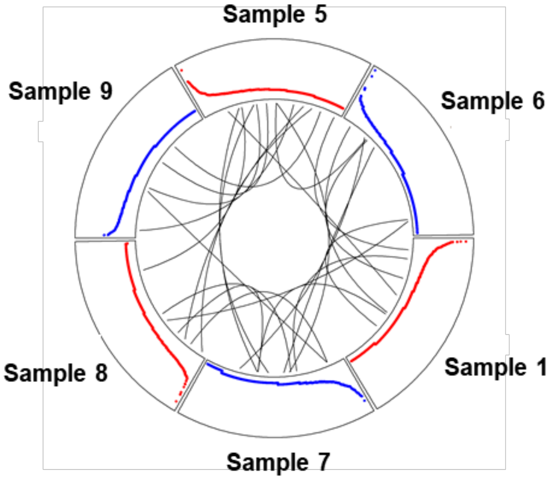

Supplementary Fig. 12 Lack of common T cell clonotypes in T-LGLL patients of three independent studies. a T cell clonotypes reported in two other T-LGLLL cohorts were defined in our patients, and also in healthy donors (Clemente MJ, et al. *Blood* 122, 4077-4085 (2013); Clemente MJ, et al. *Blood* 118, 4384-4393 (2011)). **b** Circos plots where segments in circles represent sharing of identical CDR3 sequences among six patients in a Blood Advances study (Kerr, C. M. et al. *Blood Adv*. 3, 917-921 (2019)). Black lines indicate arcs connecting patients sharing identically rearranged CDR3 sequences among individuals. Red and blue curves are proportional to clone sizes.

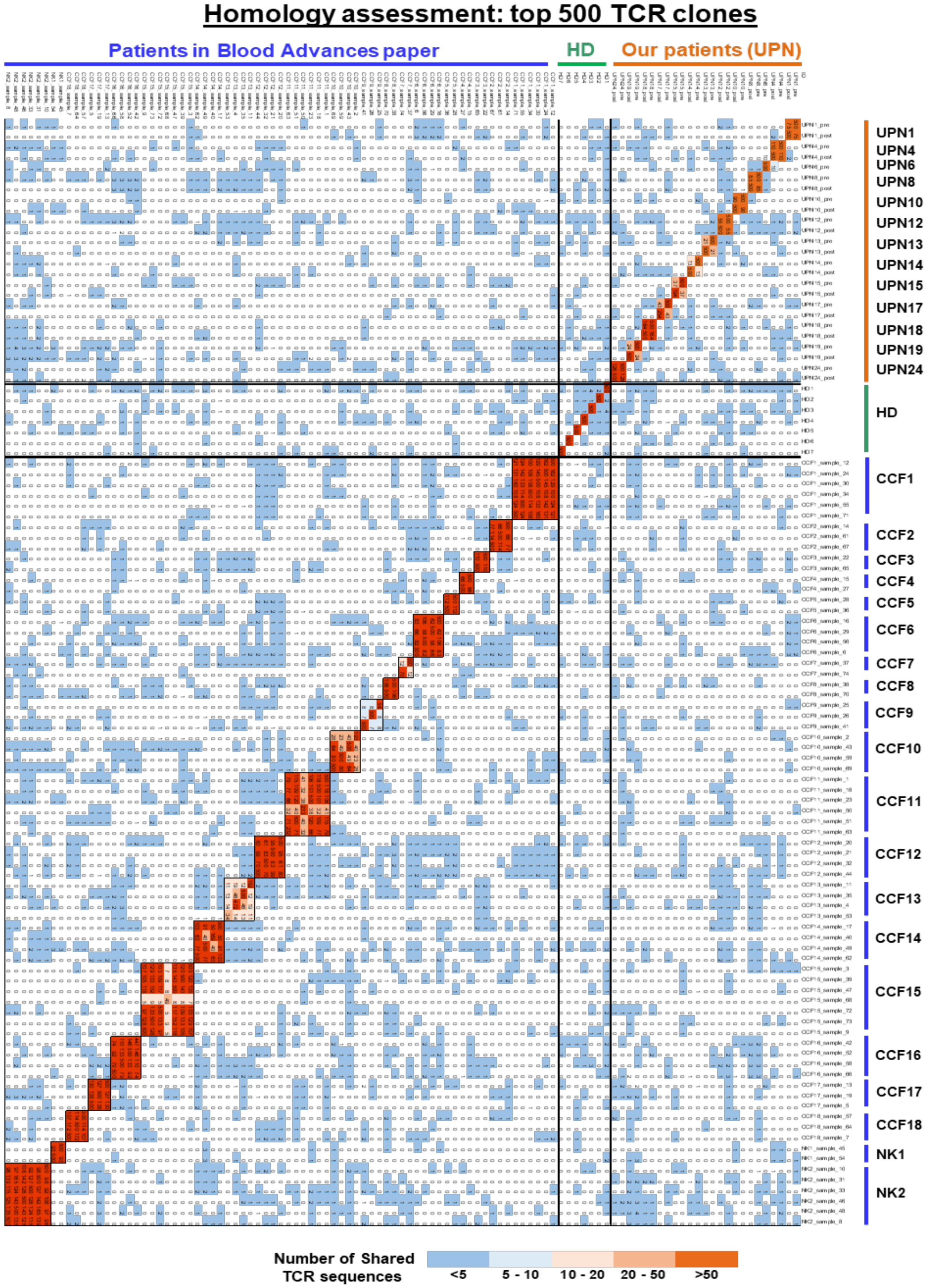

Supplementary Fig. 13 Lack of common T cell clonotypes in T-LGLL patients in ours and an independent study. A heatmap plot showing sharing among top 500 TCR clones of serial samples of 13 patients and seven healthy donors in our cohort, and 20 patients in a Blood Advances study (Kerr, C. M. et al. *Blood Adv*. 3, 917-921 (2019)). On both x- and y-axes, there were samples of patients and healthy donors, and paired samples of the same patient were adjacent. Numbers indicate counts of identical TCR clones shared among samples. A color scheme (dark orange to dark blue) represents the number of shared CDR sequences from high to low. HD, healthy donor; UPN, unique patient number.

Sequences and corresponding weblogs of top TCR specificity groups

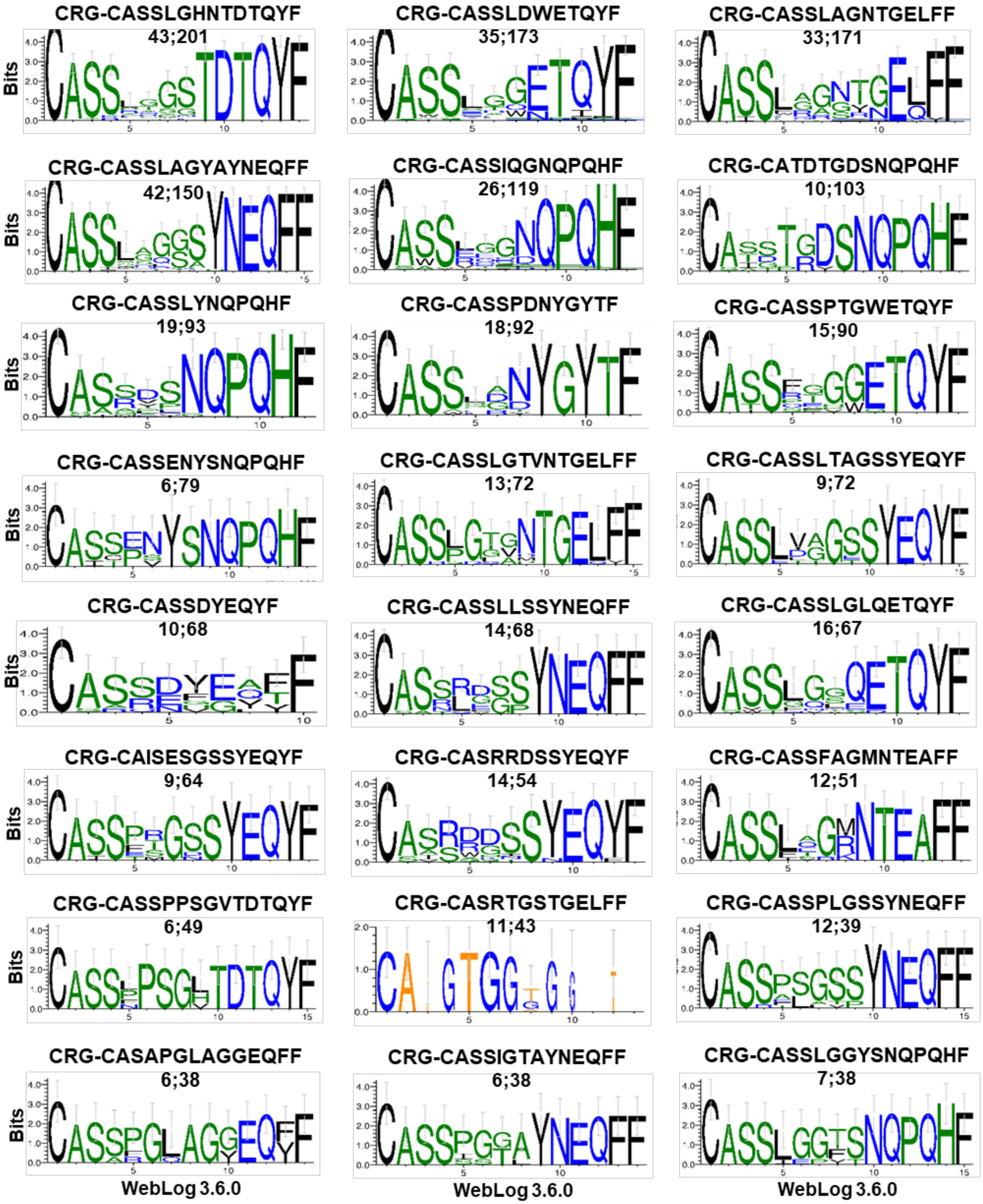

Supplementary Fig. 14 Sequences and corresponding weblogs of top TCR specificity groups with more than five different clones. In numbers A;B following CRG sequences, the A indicates the number of clones contained in this CRG; the B indicates frequency of this CRG in all cells.

Sequences and corresponding weblogs of top TCR specificity groups

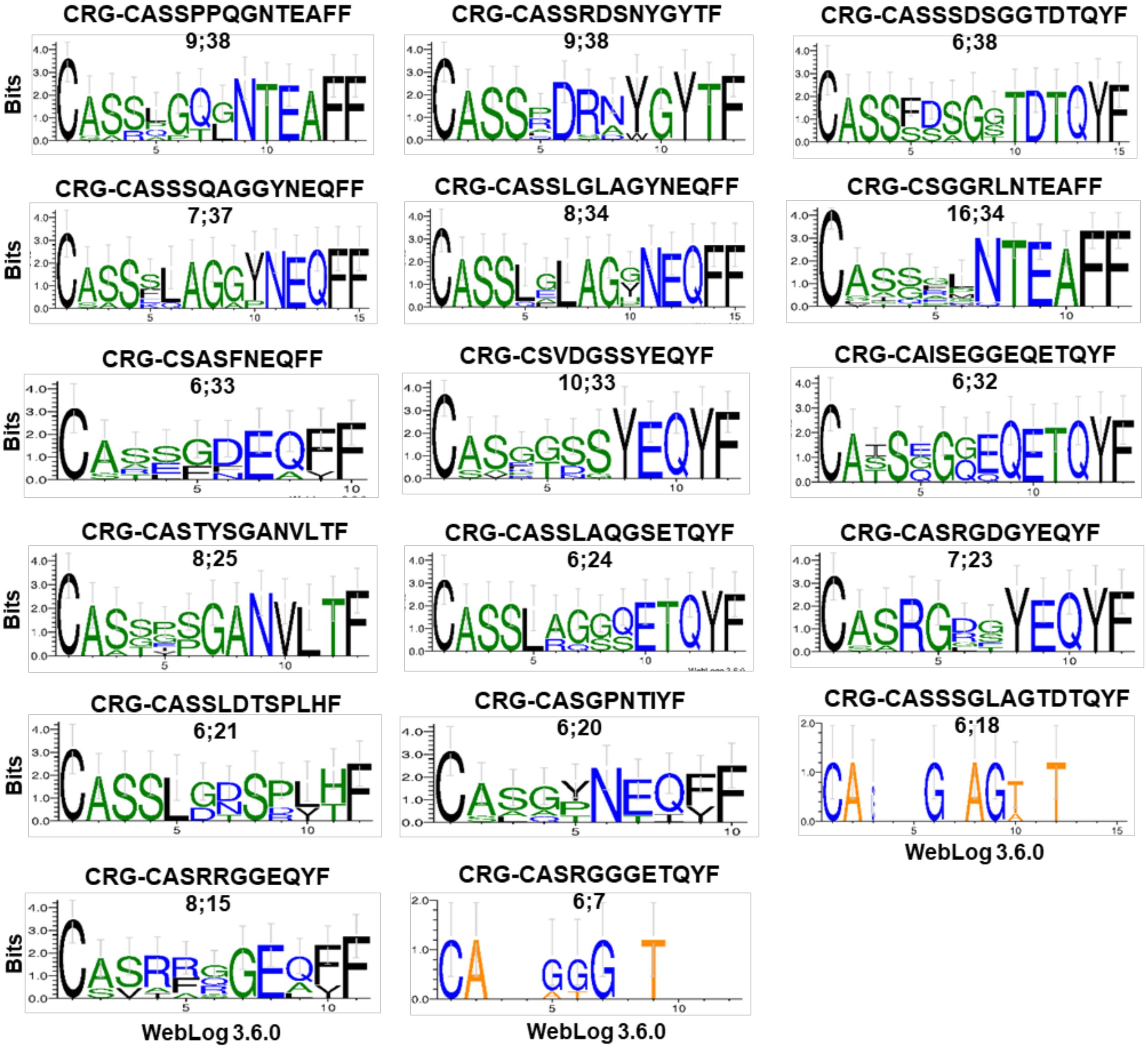

Supplementary Fig. 15 Sequences and corresponding weblogs of top TCR specificity groups with more than five different clones (continued from Supplementary Fig. 14). In numbers A;B following CRG sequences, the A indicates the number of clones contained in this CRG; the B indicates frequency of this CRG in all cells.

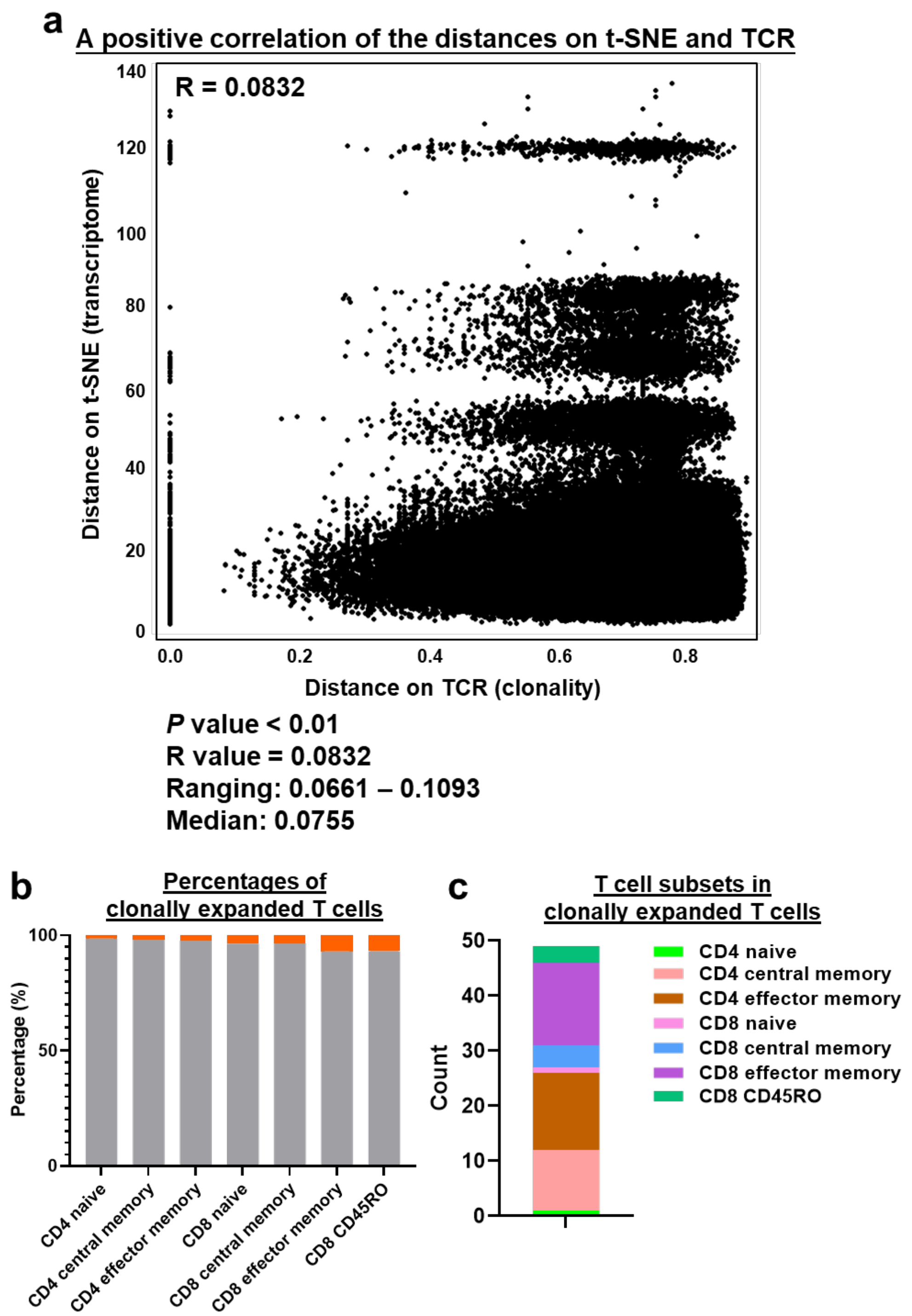

Supplementary Fig. 16 TCR usage shapes T cell phenotypes. a Calculation of correlation of distances on a t-SNE plot (based on transcriptome) and distances on a TCR plot (based on clonality) for single T cells. A distance on t-SNE (transcriptome) was in a positive correlation with a distance on TCR (clonality) with a *P* value < 0.01. A Pearson correlation test. **b** A bar chart showing percentages of clonally expanded T cells in T cell subsets. Only a small proportion of T cells was defined to be clonally expanded based on identical CDR3 sequences, but there were more in effector memory T cells and $CD45RO^+CD8^+$ T cells. **c** T cell subsets were assessed in clonally expanded T cells, and the majority of expanded T cells were phenotypically effector memory T cells and $CD45RO^+CD8^+$ T cells.

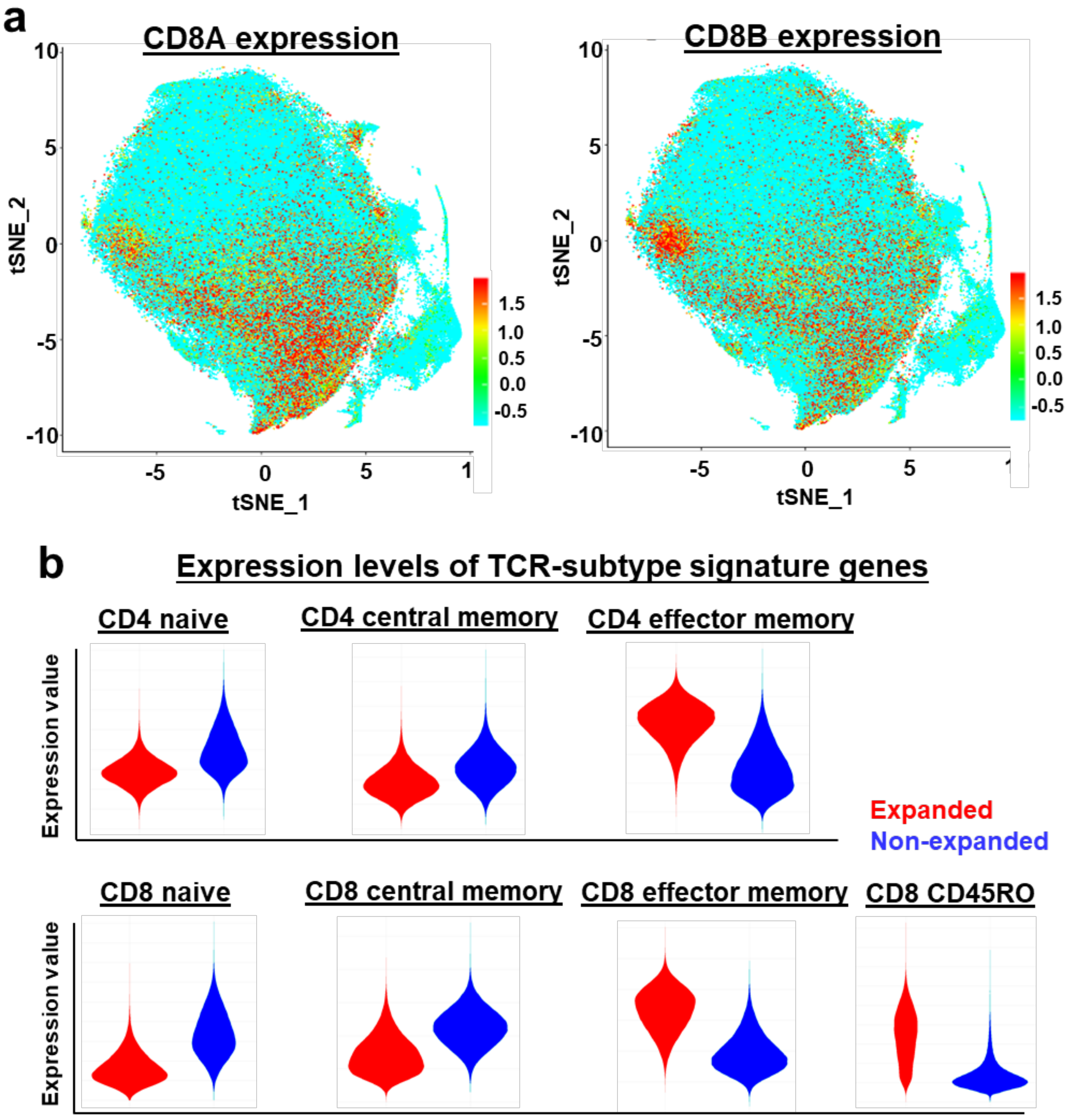

Supplementary Fig. 17 Clonally expanded T cells are mostly effector memory T cells. a The same t-SNE plot in Fig. **4a**, colored with CD8A and CD8B expression. **b** Expression levels (defined by AUC scores) of TCR subtype signature genes (naïve, central memory and effector memory) in expanded and non-expanded $CD4^+$ and $CD8^+$ T cells.

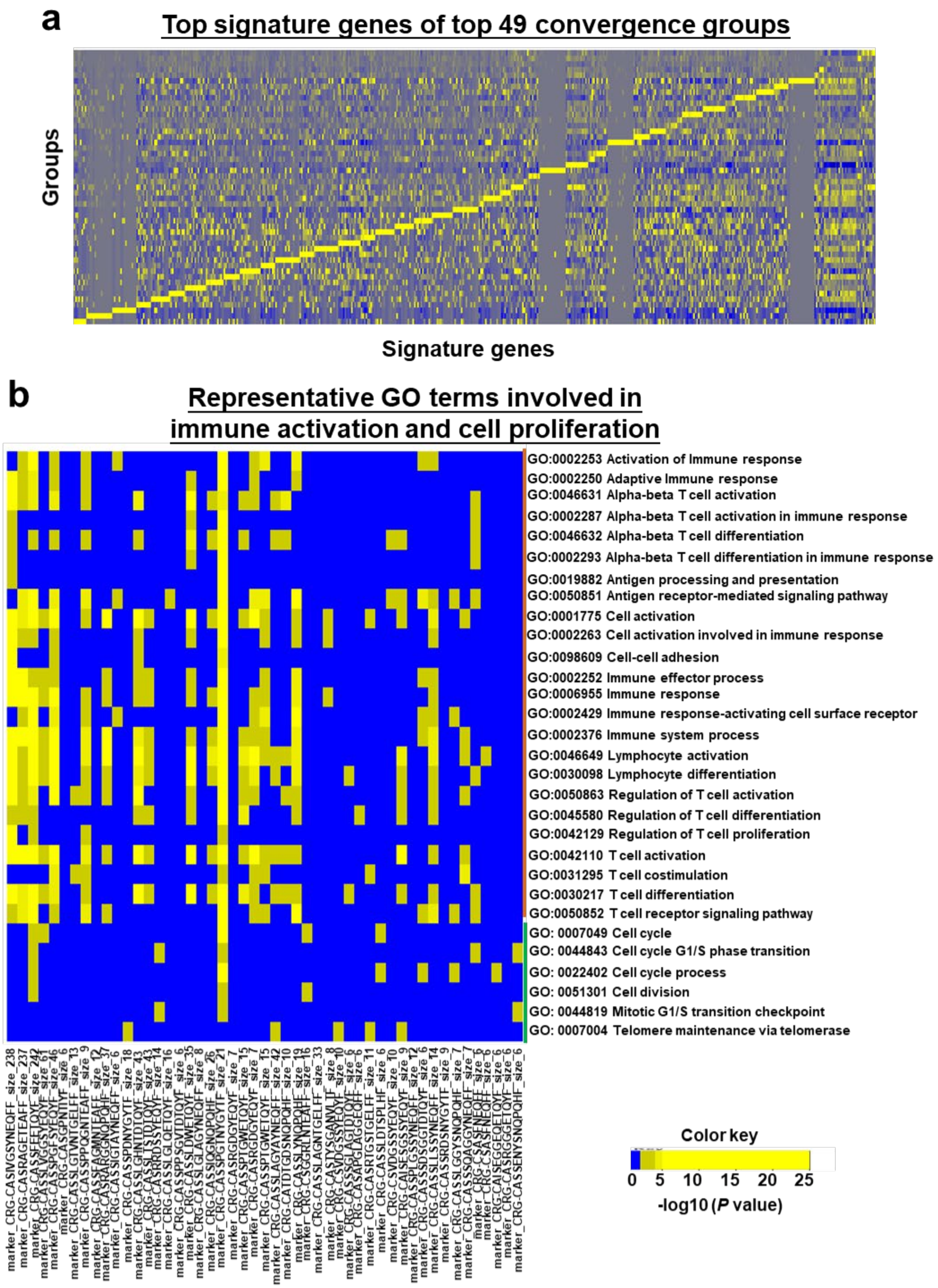

Supplementary Fig. 18 Transcriptome analysis of GLIPH-clustered TCRs. a A heatmap showing top signature genes of top 49 CRGs defined by Grouping of Lymphocyte Interactions by Paratope Hotspots (GLIPH) analysis. Differentially expressed genes were defined by comparing gene expression of T cells in a specific GLIPH CRG with all other cells. **b** GO terms of defined CRG specific genes were analyzed and -log10 (*P* value) of representative GO terms involved in immune activation and cell proliferation were plotted. A Fisher's exact test.

a **Top four convergence groups enriched in patients**

Group	Count in T-LGL	Count in healthy	*P* value
CRG-CASSPGTNYGYTF_size_21	3075	17	0.0000
CRG-CASIVGSYNEQFF_size_238	1415	559	0.0000
CRG-CASRAGETEAFF_size_237	639	212	0.0000
CRG-CASSLVGGSYEQYF_size_61	232	59	0.0000

Total cell count: T-LGL patients 233,152 and healthy donors 150,513.

b **Top four most prevalent viruses in patients**

Epitope	Antigen	Source organism	Clone size			*P* value
			Patients-pre	Patients-post	Healhty donors	(higher in pre versus HD)
FLRGRAYGL	Nuclear antigen EBNA-3	Human herpesvirus 4 (Epstein-Barr virus)	12	12	0	0.006
KLGGALQAK	55 kDa immediate-early protein 1	Human herpesvirus 5 strain AD169 (Human cytomegalovirus (strain AD169))	72	51	24	0.025
GILGFVFTL	Matrix protein 1	Influenza A virus	47	33	14	0.031
GLCTLVAML	Transcriptional regulator IE63 homolog	Human herpesvirus 4 (Epstein-Barr virus)	44	15	13	0.035

Supplementary Fig. 19 T-LGLL specific CRGs and imputed potential common antigens. **a** Top four CRGs enriched in T-LGLL patients rather than in healthy donors. **b** A table of top four most prevalent viruses in T-LGLL patients rather than in healthy donors, imputed from reported virus-specific CDR3 sequences in TCRmatch. A Fisher's exact test. *P* values are shown in the figure.

a **The most prevalent viruses in patients and healthy donors**

Antigen species	Sum in pre	Sum in HD	Patient_pre total	HD total	% in pre	% in HD
CMV	428	198	33994	15695	1.26	1.26
EBV	159	57	33994	15695	0.47	0.36
Influenza A	109	44	33994	15695	0.32	0.28
Homo sapiens*	64	24	33994	15695	0.19	0.15
HIV-1	47	15	33994	15695	0.14	0.10
DENV	13	8	33994	15695	0.04	0.05
YFV	12	6	33994	15695	0.04	0.04
MCMV	7	3	33994	15695	0.02	0.02
SARS-CoV-2	5	3	33994	15695	0.01	0.02
M. tuberculosis	4	0	33994	15695	0.01	0.00
HCV	3	4	33994	15695	0.01	0.03
LCMV	2	3	33994	15695	0.01	0.02
Plasmodium berghei	1	0	33994	15695	0.00	0.00
SIV	0	2	33994	15695	0.00	0.01
HTLV	0	1	33994	15695	0.00	0.01

*Homo sapiens (virus types as antigen species), with annotation "Parent species of the antigen, to the best clade resolution possible (e.g. HIV-1, HIV1*HXB2)", as stated at https://github.com/antigenomics/vdjdb-db/blob/master/README.md.

b **A linear correlation of virus-specific CDR3 sequences**

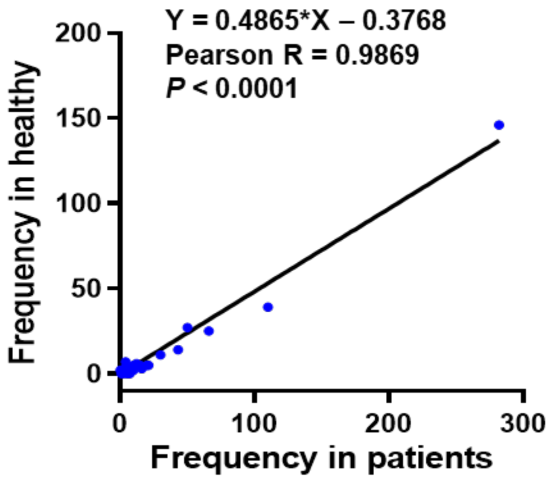

Supplementary Fig. 20 Lack of specific common antigens in T-LGLL. a A table of the most prevalent viruses in patients and healthy donors, imputed from reported virus-specific CDR3 sequences in VDJdb. **b** Frequency of virus-specific CDR3 sequences in T-LGLL patients and healthy donors were in a linear correlation. A Pearson correlation test. *P* value is shown in the figure.

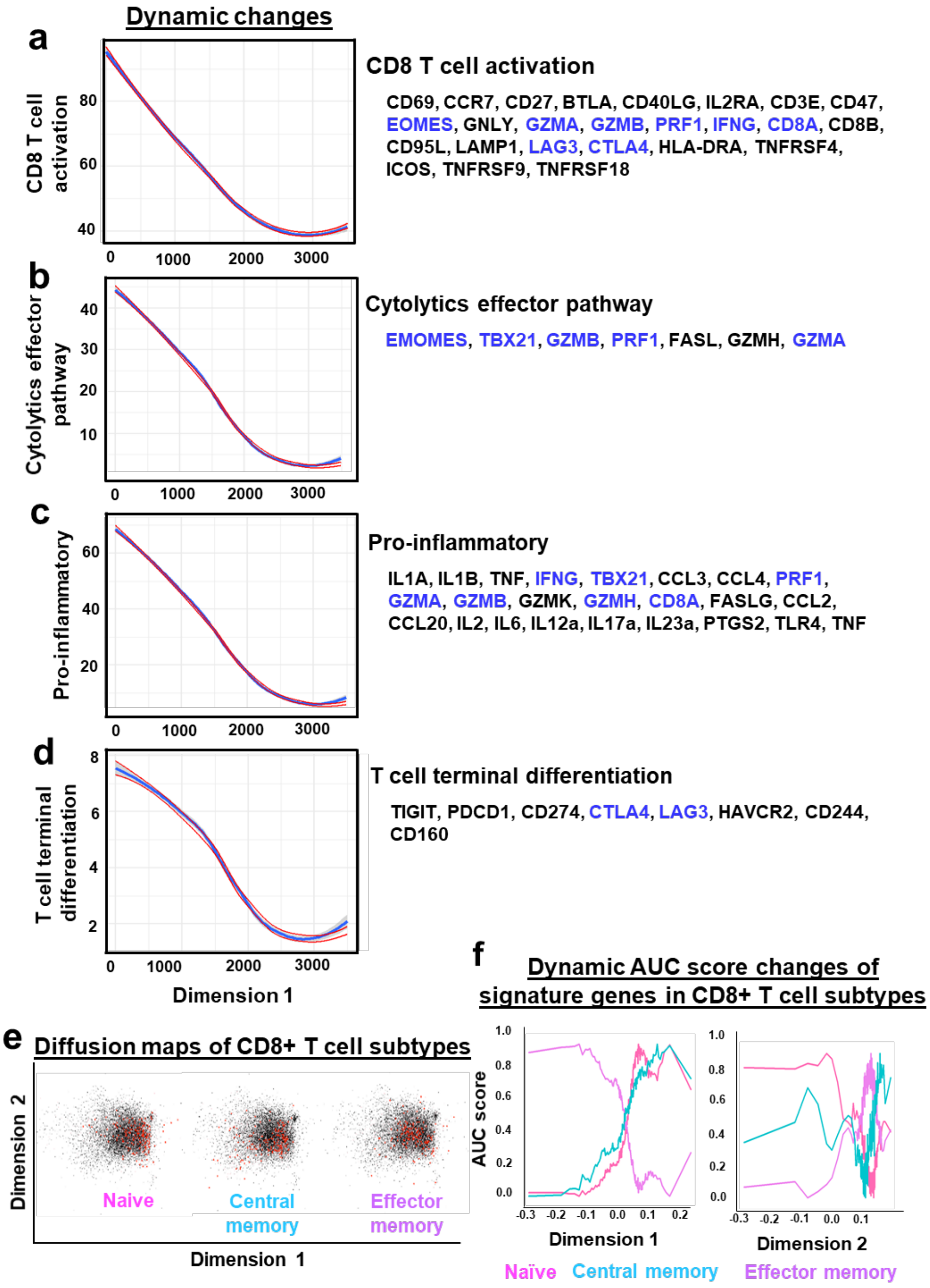

Supplementary Fig. 21 Gene modules work synergistically in shaping T cell phenotypes. Curves indicate dynamic changes of CD8^{+} T cell activation (**a**), the cytolytics effector pathway (**b**), pro-inflammatory genes (**c**) and T cell terminal differentiation (**d**) on dimension 1 (x-axis) revealed on diffusion maps in Figure **4d**. Imputed expression of above four components were plotted on y-axis. A red line indicates 5 – 95% interval, illustrated by shaded area; a blue line indicates a medium. Gene lists of CD8^{+} T cell activation, the cytolytics effector pathway, pro-inflammatory and T cell terminal differentiation components are shown on the right. **e** A diffusion map of CD8^{+} T cells on dimension 1 and dimension 2, which are colored (red) with cell identity as naïve, central memory or effector memory T cell subsets, and all other cells are colored as grey background. **f** Dynamic changes of AUC scores of naïve (pink), central memory (blue) and effector memory (purple) signature genes on dimension 1 and dimension 2, respectively.

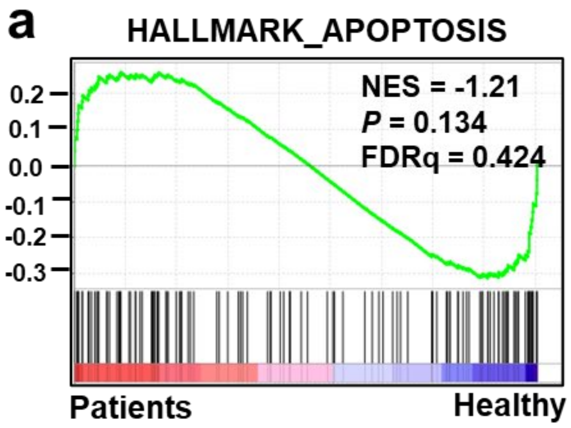

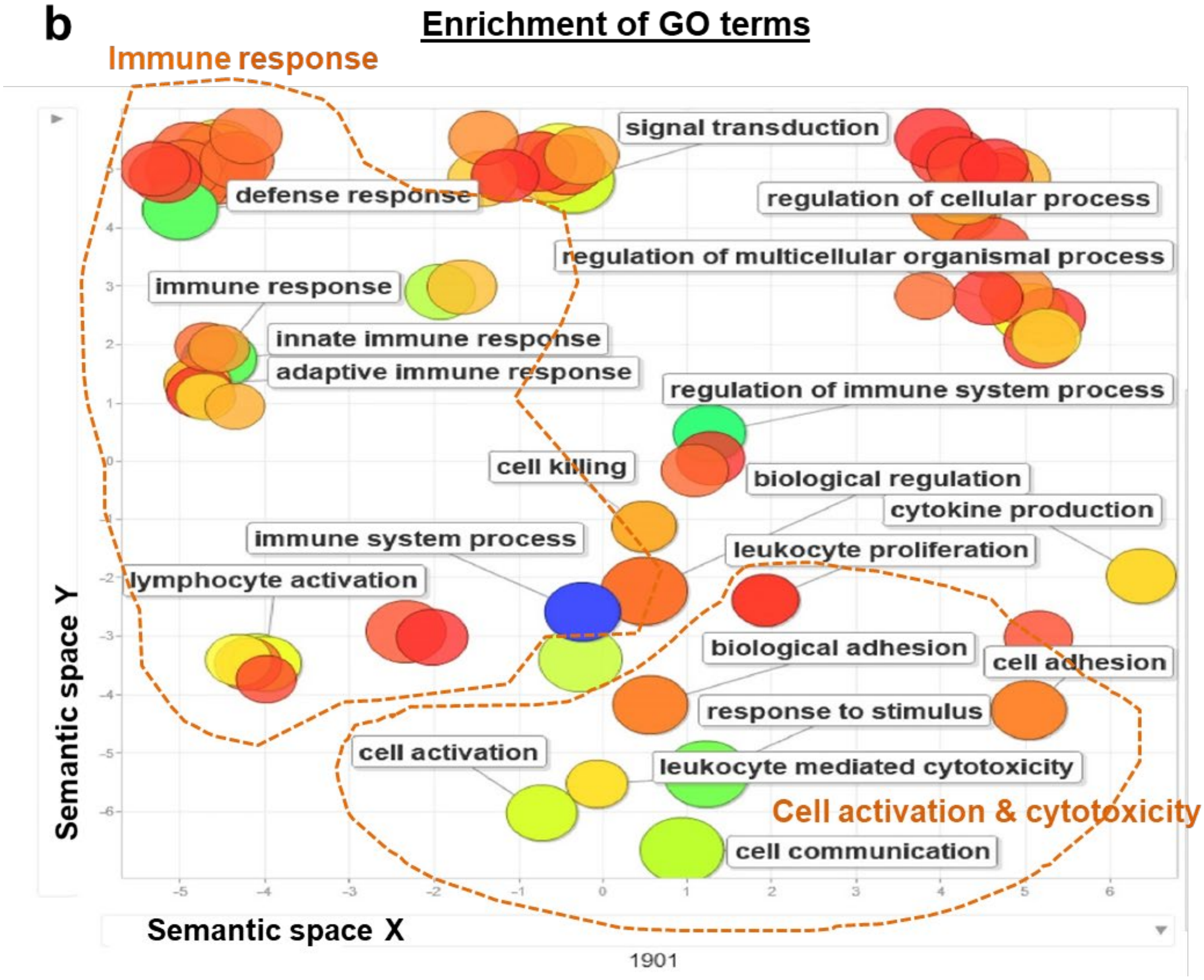

Supplementary Fig. 22 Dysregulated gene programs in T-LGLL. a A GSEA plot of a HALLMARK_Apoptosis gene set with expressed genes in T-LGLL patients compared to those in healthy donors. GESA based on a Kolmogorov Smirnov test. **b** A REVIGO plot showing enrichment of GO terms (generated using differentially expressed genes in T-LGLL patients) in cell activation, cytotoxicity and immune response.

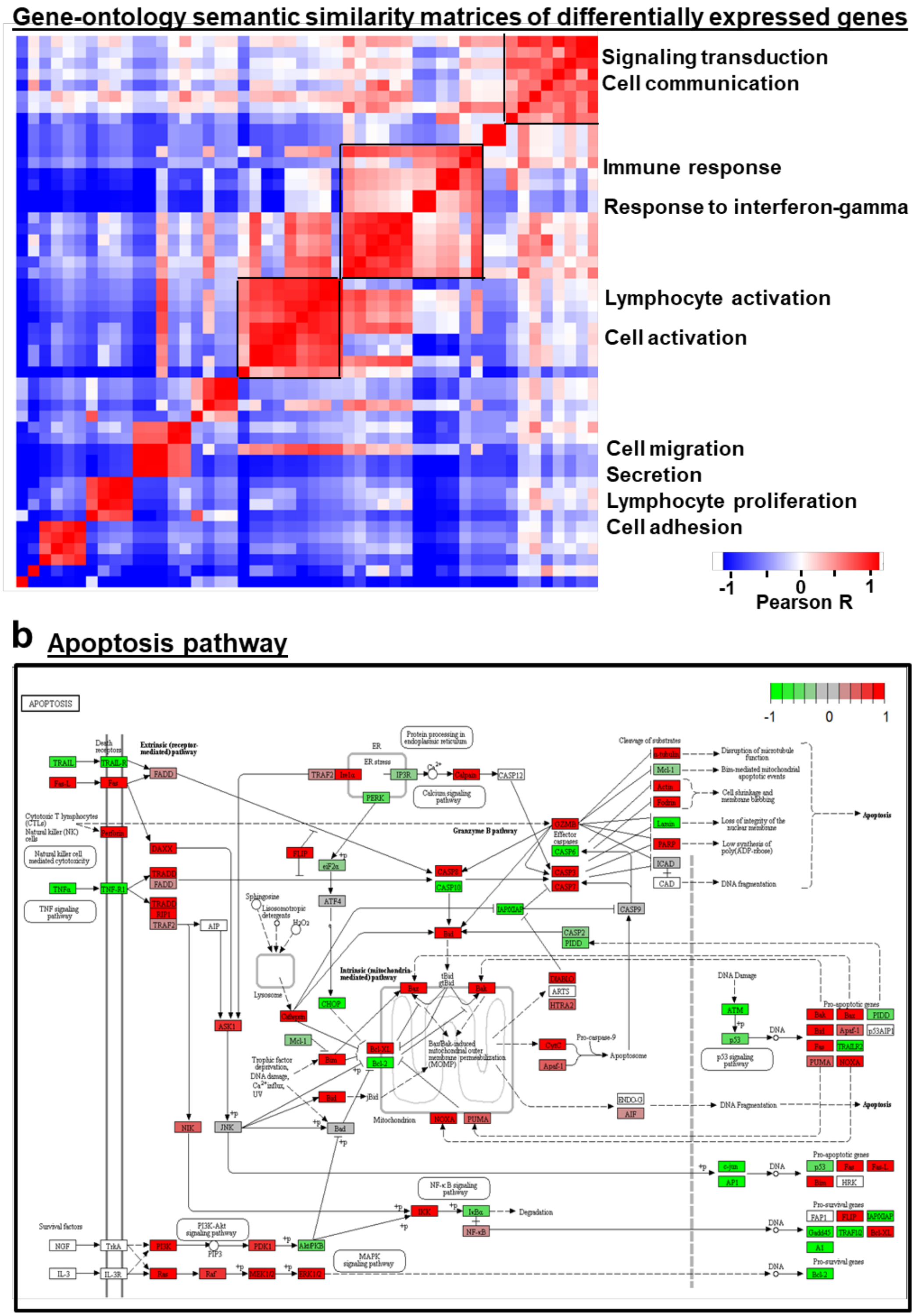

Supplementary Fig. 23 Dysregulated pathways in T-LGLL. a A gene-ontology semantic similarity matrix of differentially expressed genes in T-LGLL. Gene ontology terms involved in similar functional matrices were adjacent and formed a block with Pearson R values ranging from -1 to 1. Terms noted on the right side depict common biological processes of the block of Gene-ontology terms. **b** A KEGG graph of the cell apoptosis pathway. Red, genes upregulated in T-LGLL; green, genes downregulated in T-LGLL.

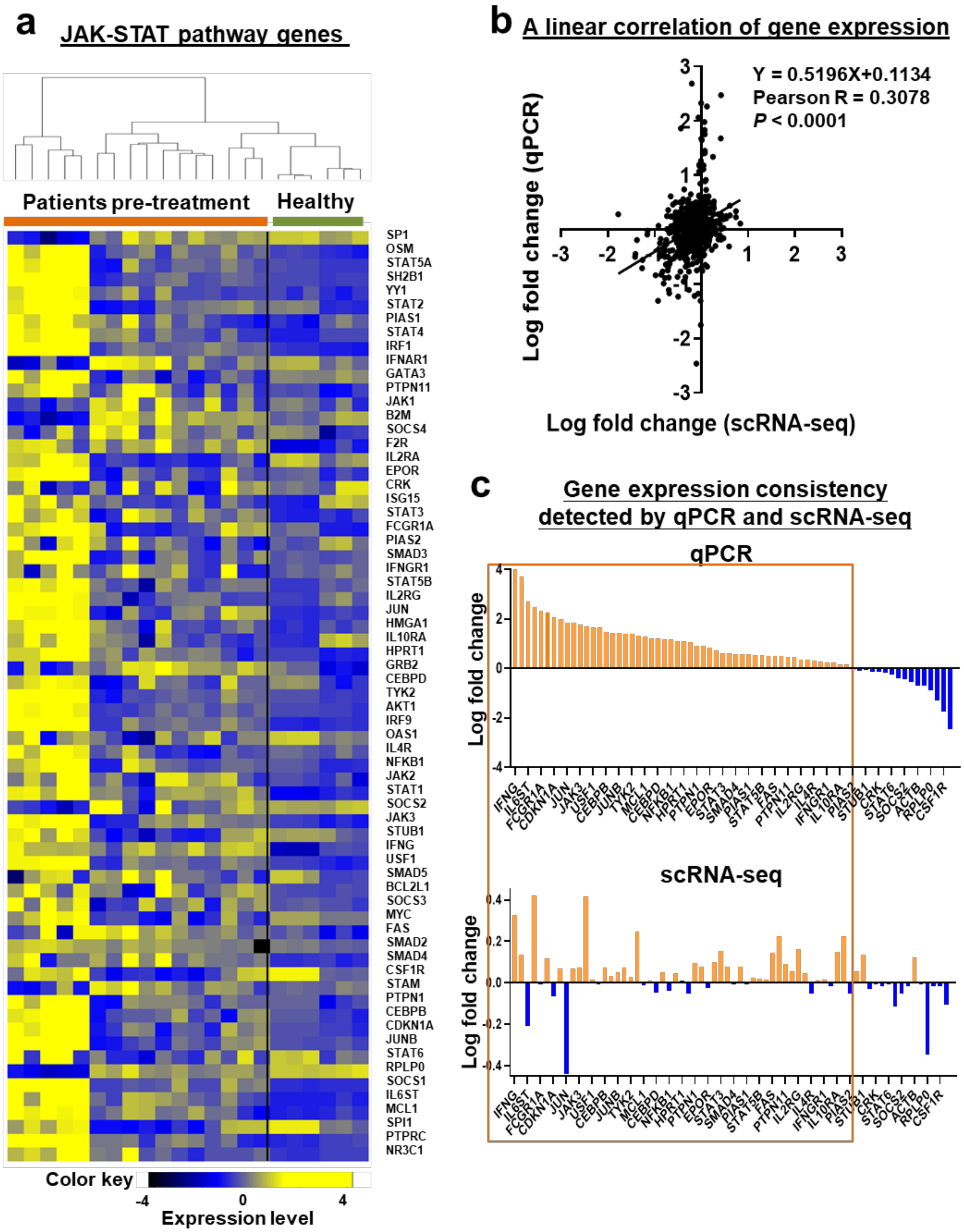

Supplementary Fig. 24 Consistency of gene expression detected using scRNA-seq and qPCR. a A heatmap of JAK-STAT pathway genes generated using scRNA-seq data, showing consistent upregulation of these genes in T-LGLL. **b** A linear correlation of gene expression alteration (log fold changes) using scRNA-seq and qPCR. A Pearson correlation test. *P* value is shown in the figure. **c** Fold changes of these genes using scRNA-seq and qPCR were largely to the same directions.

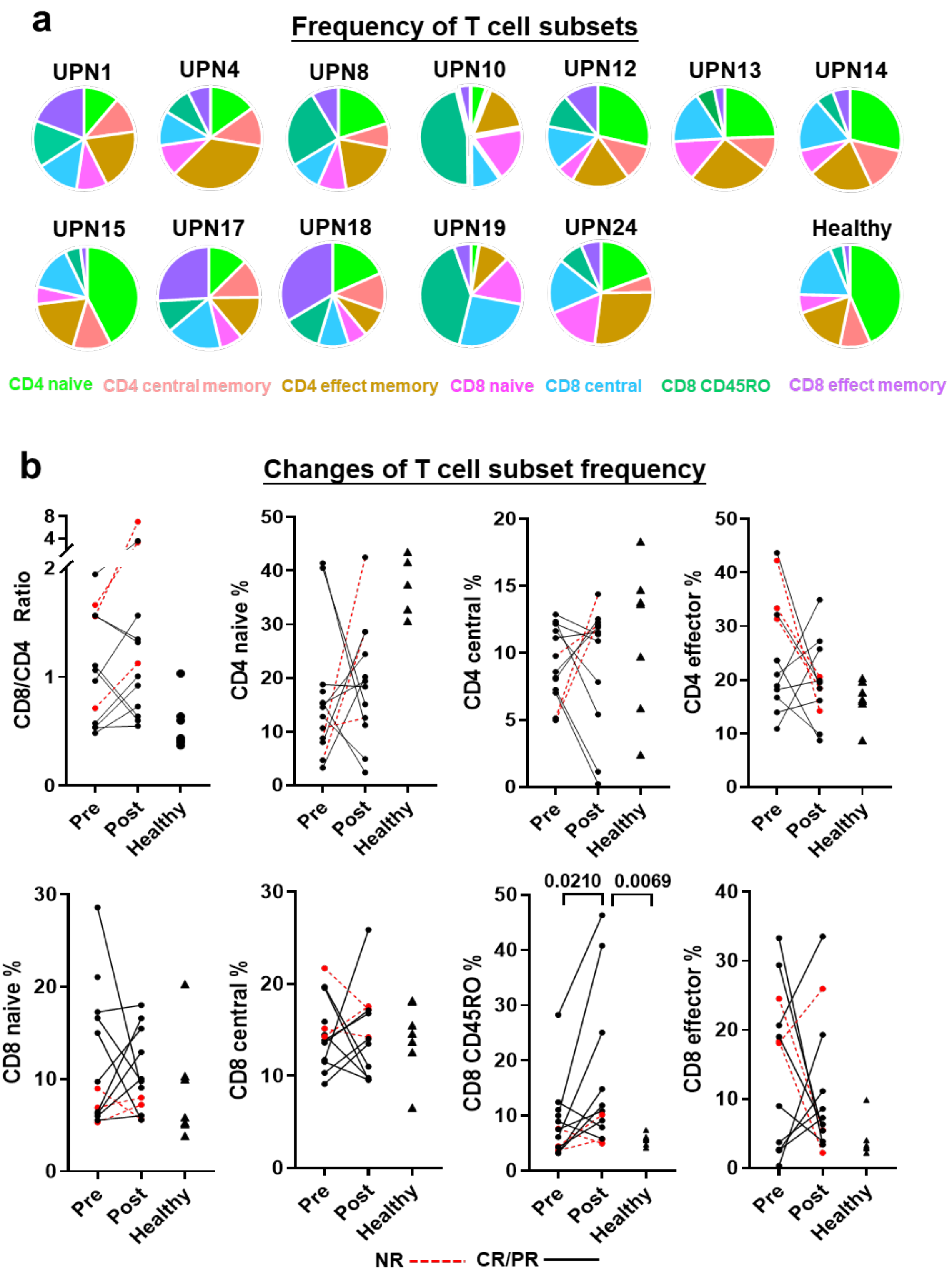

Supplementary Fig. 25 Changes of T cell subsets after treatment. a Pie charts showing percentages of T cell subsets in individual patients after treatment and healthy donors. A color scheme is the same in Fig. **1e, f**. **b** Plots of changes of T cell subsets in patients after treatment, compared to those before treatment and healthy controls. A two-sided paired t-test between patients' samples before and after treatments (n = 12); a two-sided unpaired t-test between patients (n = 13) and healthy donors (n = 7). *P* values are shown in the figure.

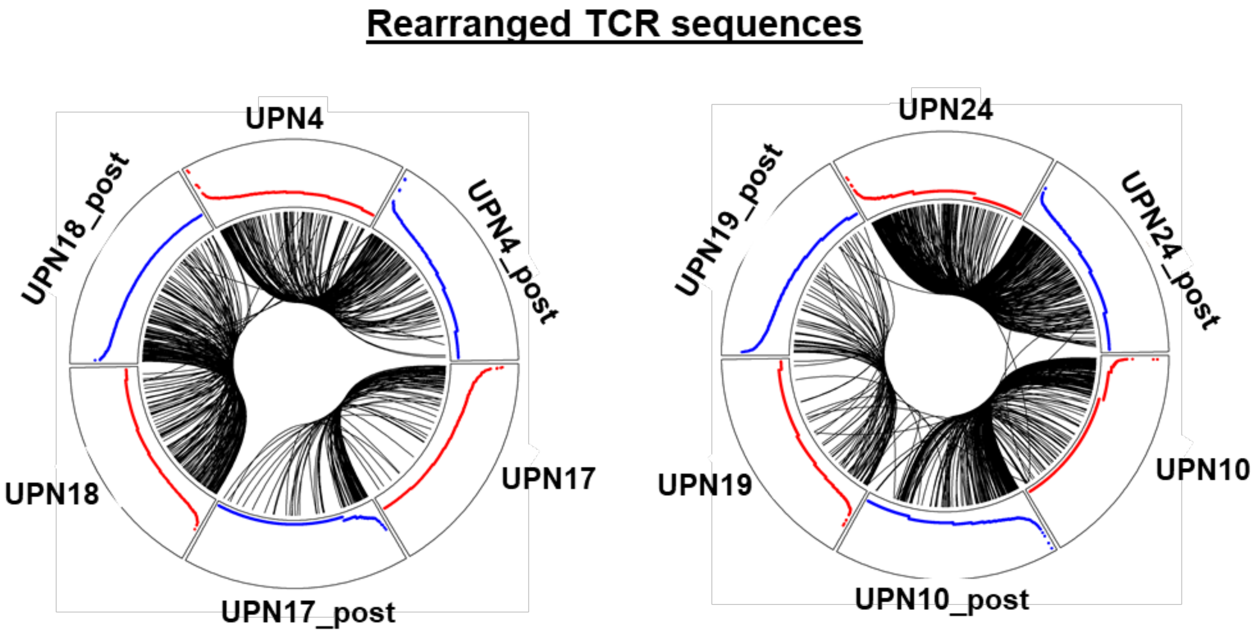

Supplementary Fig. 26 Immunosuppressive treatment modulates clonality in T-LGLL. Circos plots where segments in circles represent individual cells yielding rearranged TCR sequences among patients or between two visits of patients. Black lines indicate arcs connecting cells sharing identically rearranged CDR3 sequences. Plots on the left and right show sharing of identical CDR3 sequences among UPNs 4, 17 and 18, and UPNs 10, 19 and 24, respectively. Red and blue curves indicate sample before and after treatments, respectively; both are proportional to clone sizes.

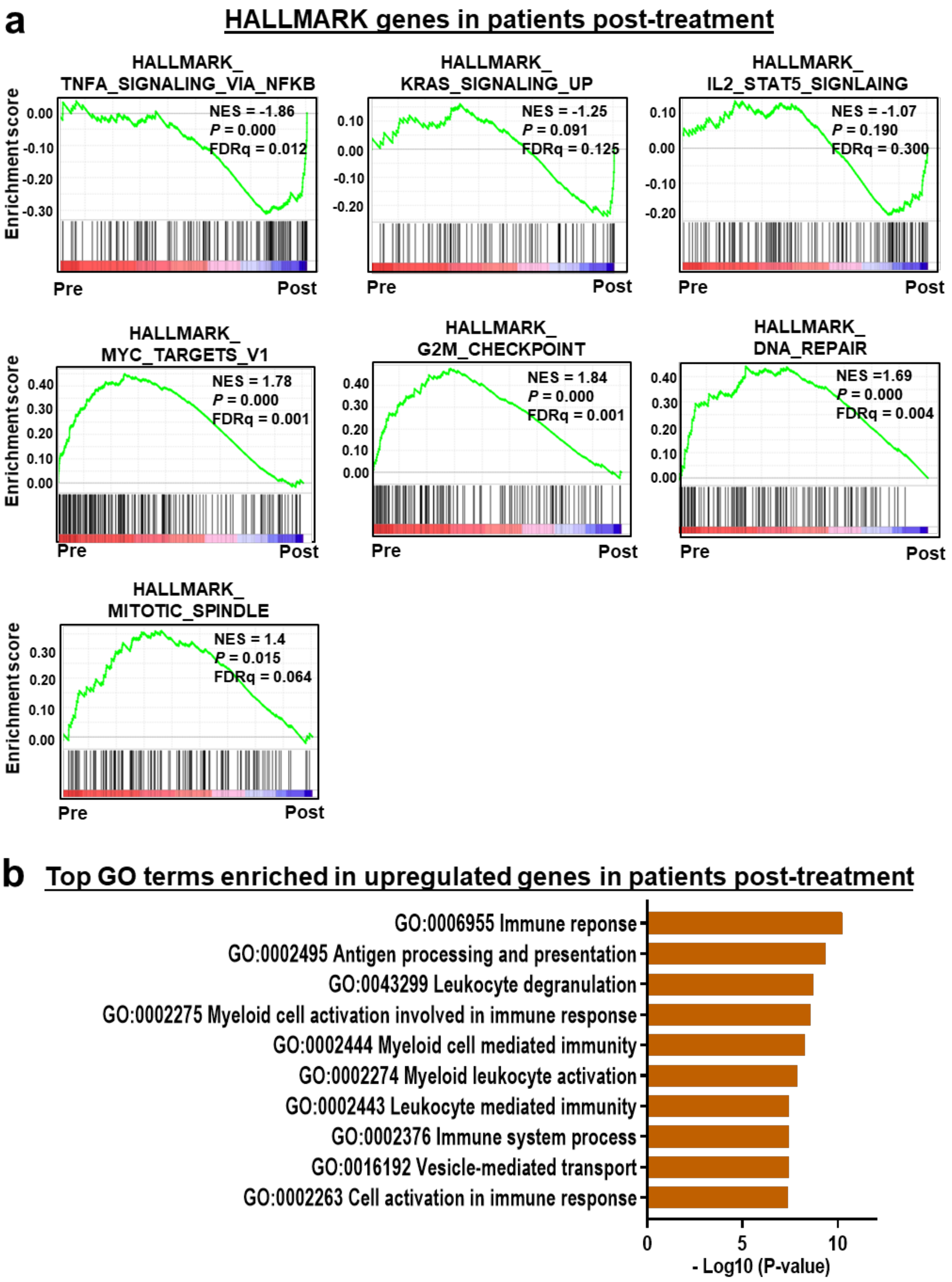

Supplementary Fig. 27 Pathway analysis of differentially expressed genes in patients post-treatment vs. pre-treatment. a GSEA plots of HALLMARK genes in T-LGLL patients after treatment, compared to those before treatment. GESA based on a Kolmogorov Smirnov test. **b** A bar chart showing top GO terms enriched in upregulated genes in T-LGLL patients after treatment, as compared to those before treatment. A Fisher’s exact test.

Four patterns of clonal kinetics of patients pre- and post-treatments
(Pattern I)

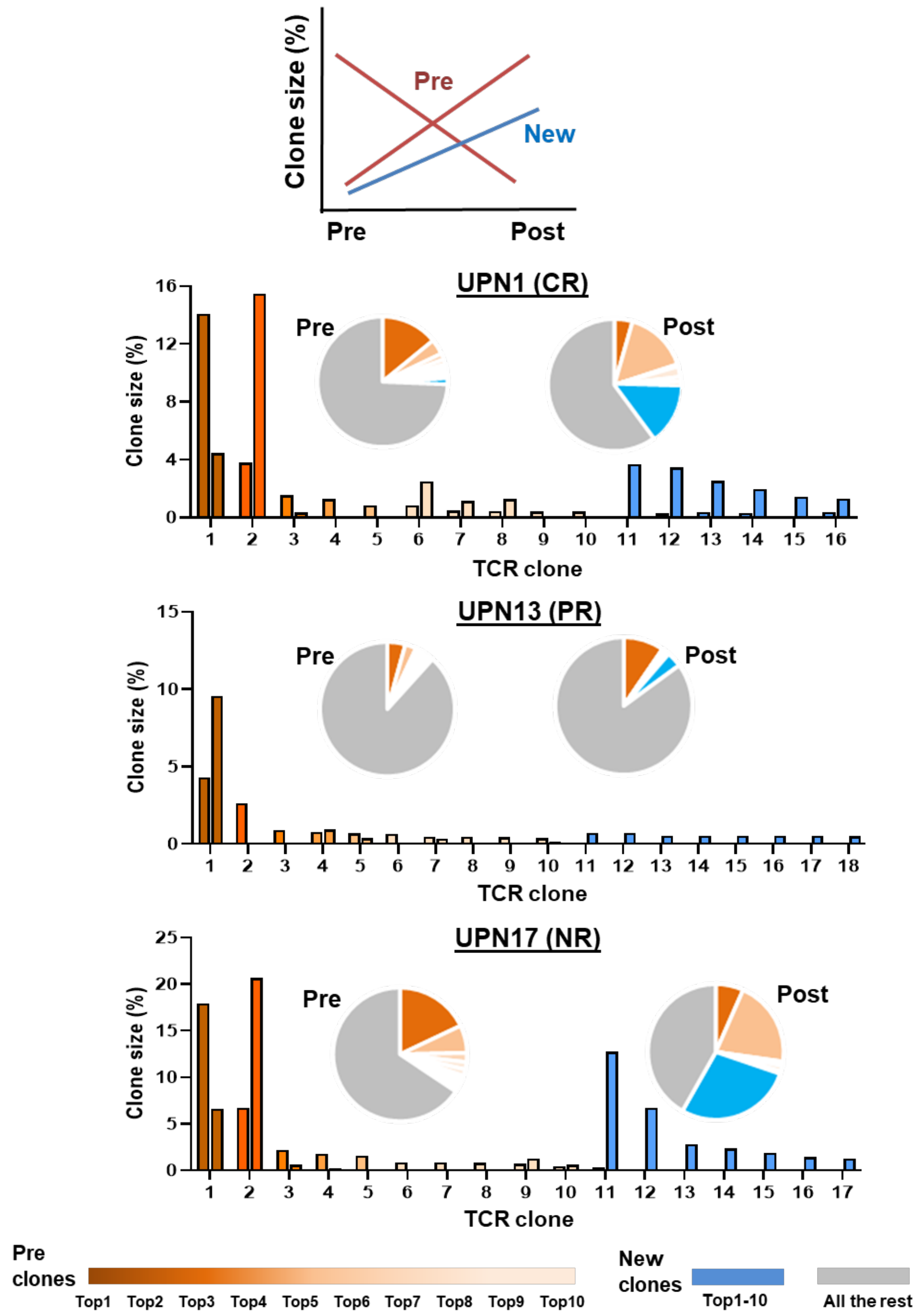

Supplementary Fig. 28 Four patterns of clonal kinetics of patients pre- and post-treatments (Pattern I). Each panel includes a diagram illustrating a pattern of clonal kinetics. **a** bar charts of clone sizes (%) and pie charts of percentages of top ten TCR clonotypes from pre- and post-treatment samples at different time points. In bar charts, paired samples of the same patient were plotted adjacent. Orange colors (ranging from dark to light orange) indicate top ten clones pre-treatment; blue colors indicate top ten clones post-treatment, but were not among top ten pre-treatment; grey colors show all the other clones.

Four patterns of clonal kinetics of patients pre- and post-treatments (Pattern II)

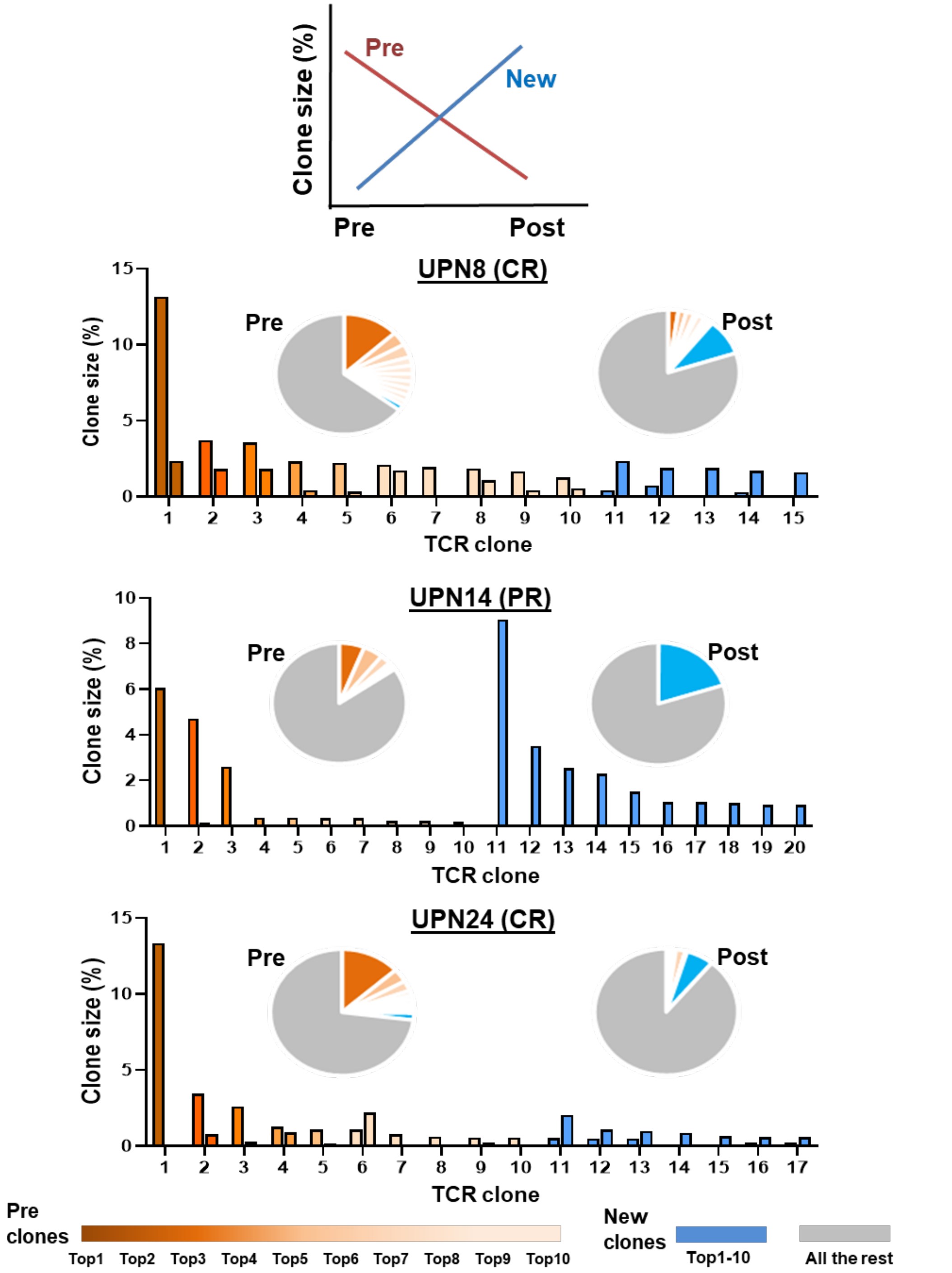

Supplementary Fig. 29 Four patterns of clonal kinetics of patients pre- and post-treatments (Pattern II). Each panel includes a diagram illustrating a pattern of clonal kinetics. **a** bar charts of clone sizes (%) and pie charts of percentages of top ten TCR clonotypes from pre- and post-treatment samples at different time points. In bar charts, paired samples of the same patient were plotted adjacent. Orange colors (ranging from dark to light orange) indicate top ten clones pre-treatment; blue colors indicate top ten clones post-treatment, but were not among top ten pre-treatment; grey colors show all the other clones.

Four patterns of clonal kinetics of patients pre- and post-treatments
(Pattern II continued)

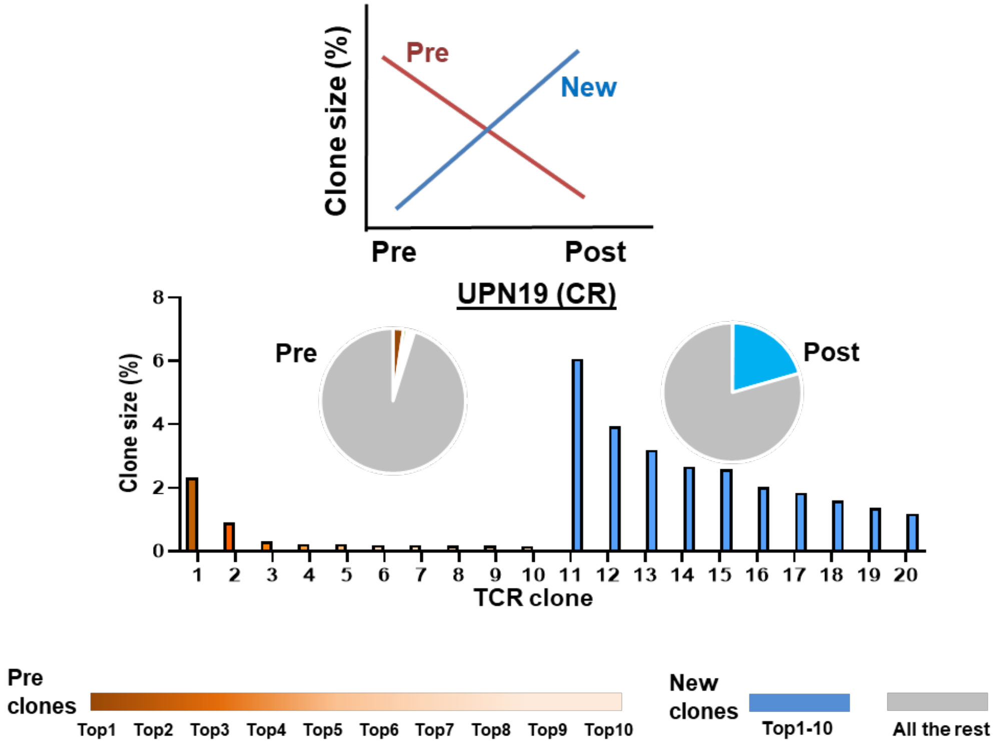

Supplementary Fig. 30 Four patterns of clonal kinetics of patients pre- and post-treatments (Pattern II, continued). Each panel includes a diagram illustrating a pattern of clonal kinetics. **a** bar charts of clone sizes (%) and pie charts of percentages of top ten TCR clonotypes from pre- and post-treatment samples at different time points. In bar charts, paired samples of the same patient were plotted adjacent. Orange colors (ranging from dark to light orange) indicate top ten clones pre-treatment; blue colors indicate top ten clones post-treatment, but were not among top ten pre-treatment; grey colors show all the other clones.

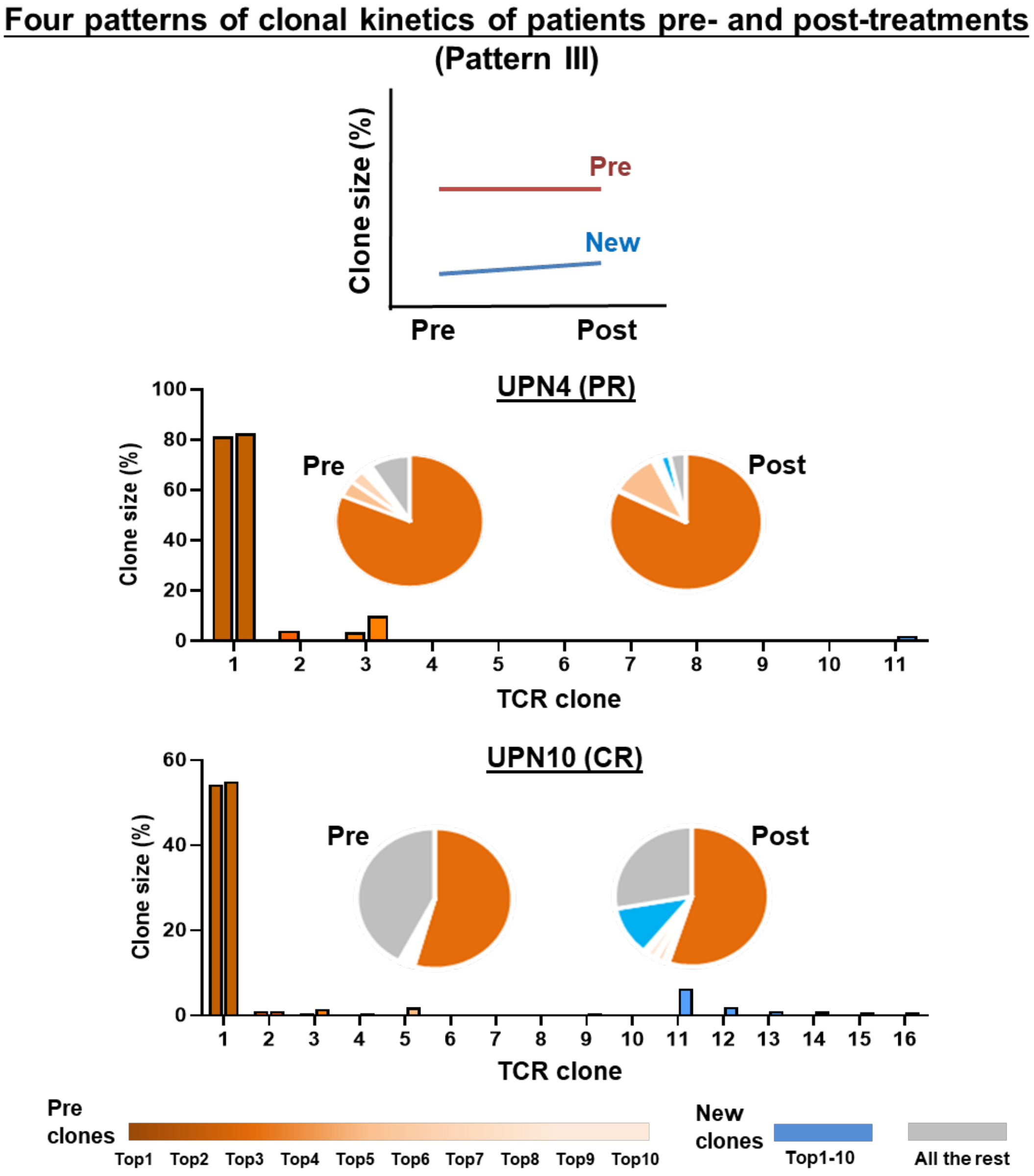

Supplementary Fig. 31 Four patterns of clonal kinetics of patients pre- and post-treatments (Pattern III). Each panel includes a diagram illustrating a pattern of clonal kinetics. **a** bar charts of clone sizes (%) and pie charts of percentages of top ten TCR clonotypes from pre- and post-treatment samples at different time points. In bar charts, paired samples of the same patient were plotted adjacent. Orange colors (ranging from dark to light orange) indicate top ten clones pre-treatment; blue colors indicate top ten clones post-treatment, but were not among top ten pre-treatment; grey colors show all the other clones.

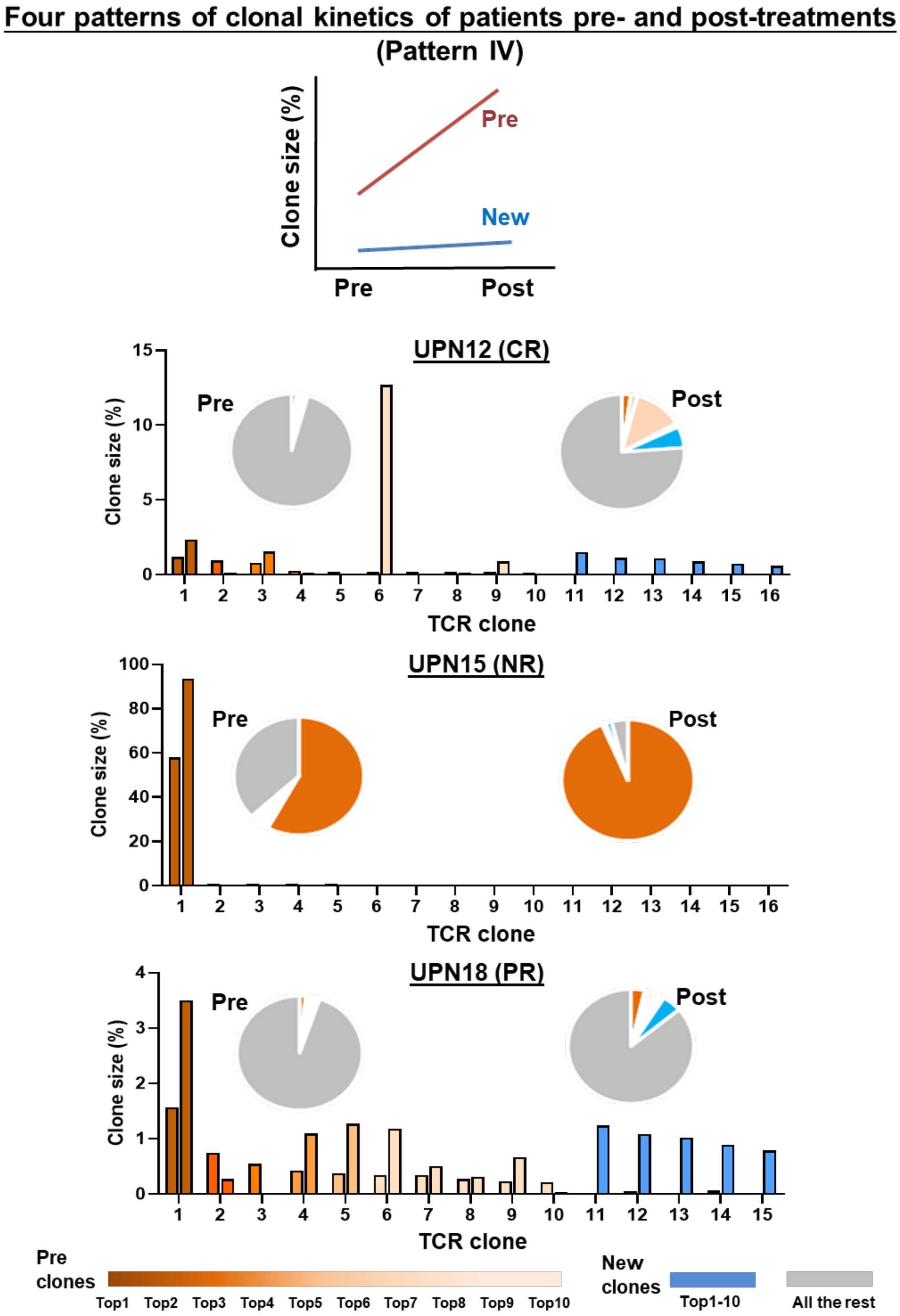

Supplementary Fig. 32 Four patterns of clonal kinetics of patients pre- and post-treatments (Pattern IV). Each panel includes a diagram illustrating a pattern of clonal kinetics. **a** bar charts of clone sizes (%) and pie charts of percentages of top ten TCR clonotypes from pre- and post-treatment samples at different time points. In bar charts, paired samples of the same patient were plotted adjacent. Orange colors (ranging from dark to light orange) indicate top ten clones pre-treatment; blue colors indicate top ten clones post-treatment, but were not among top ten pre-treatment; grey colors show all the other clones.

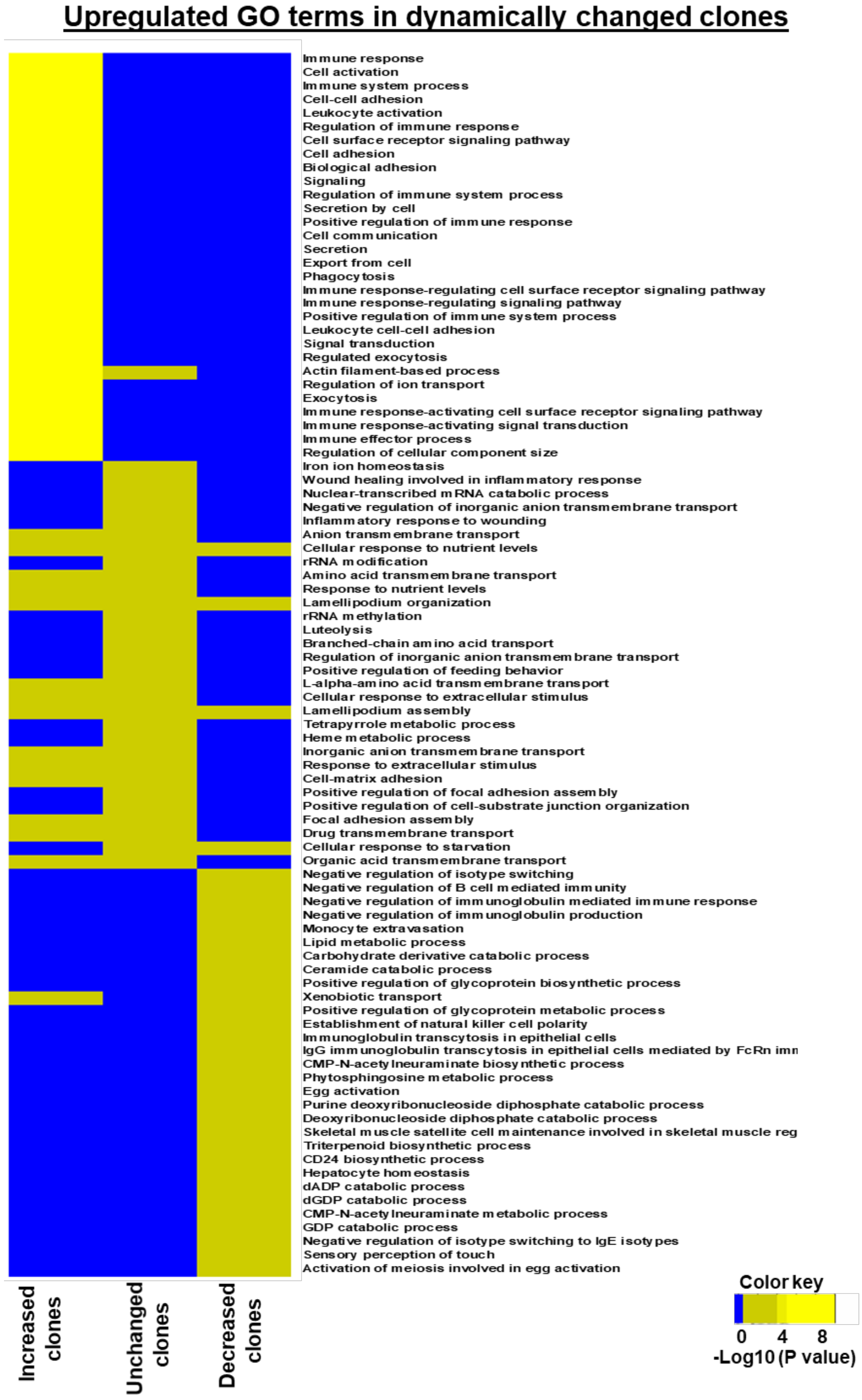

Supplementary Fig. 33 Upregulated GO terms in dynamically changed clones. A heatmap shows expression levels of upregulated GO terms in increased, unchanged and decreased clones in T-LGLL patients post- versus pre-treatment. GO terms related with immune response and cell activation were predominantly upregulated in increased clones, but not in unchanged or decreased clones. Fisher's exact test.

Downregulated GO terms in dynamically changed clones

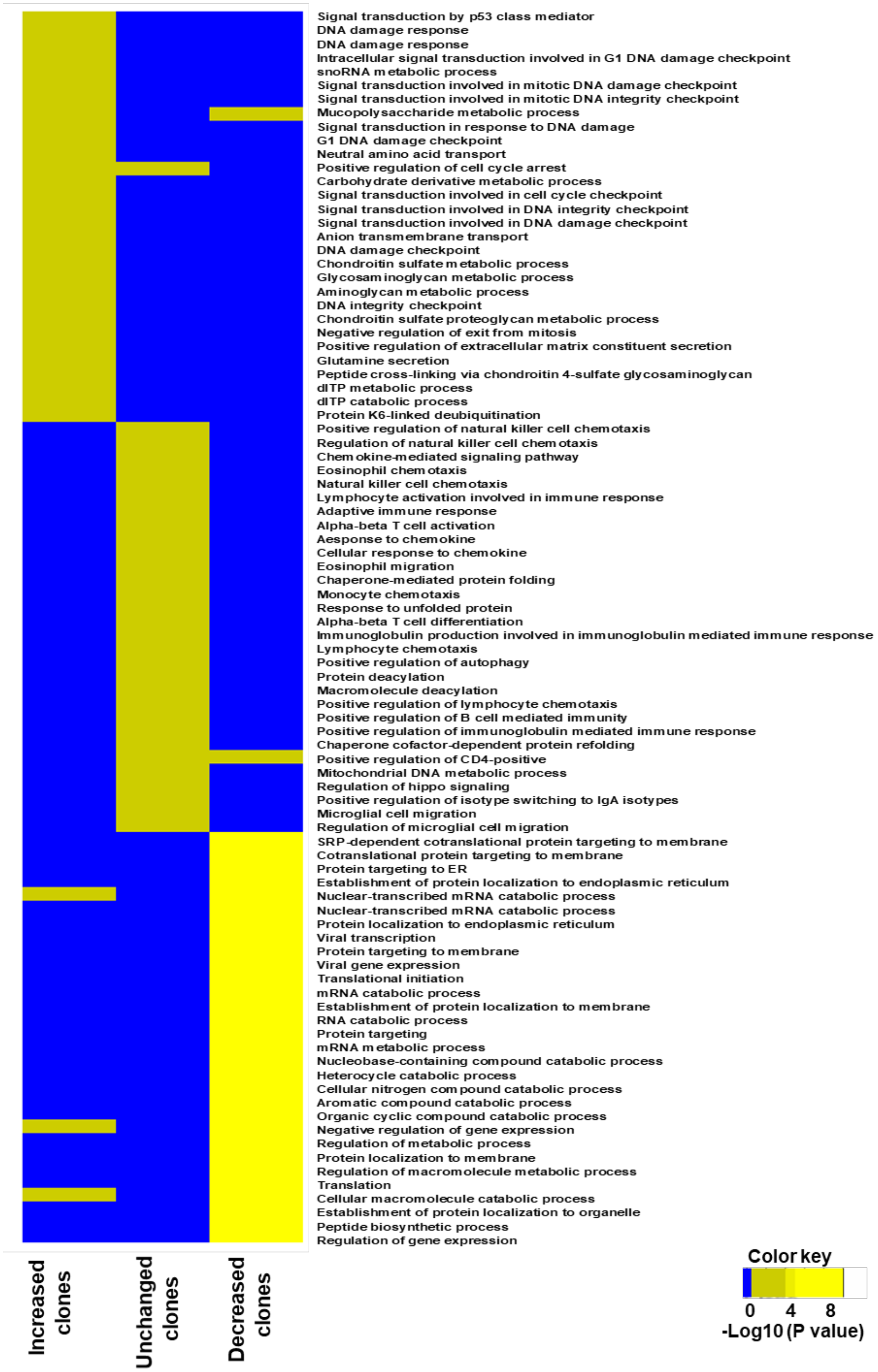

Supplementary Fig. 34 Downregulated GO terms in dynamically changed clones. A heatmap shows expression levels of downregulated GO terms in increased, unchanged and decreased clones in T-LGLL patients post-treatment vs. pre-treatment. GO terms related with immune response and protein translation were decreased in unchanged or decreased clones, but not in increased clones. A Fisher's exact test.

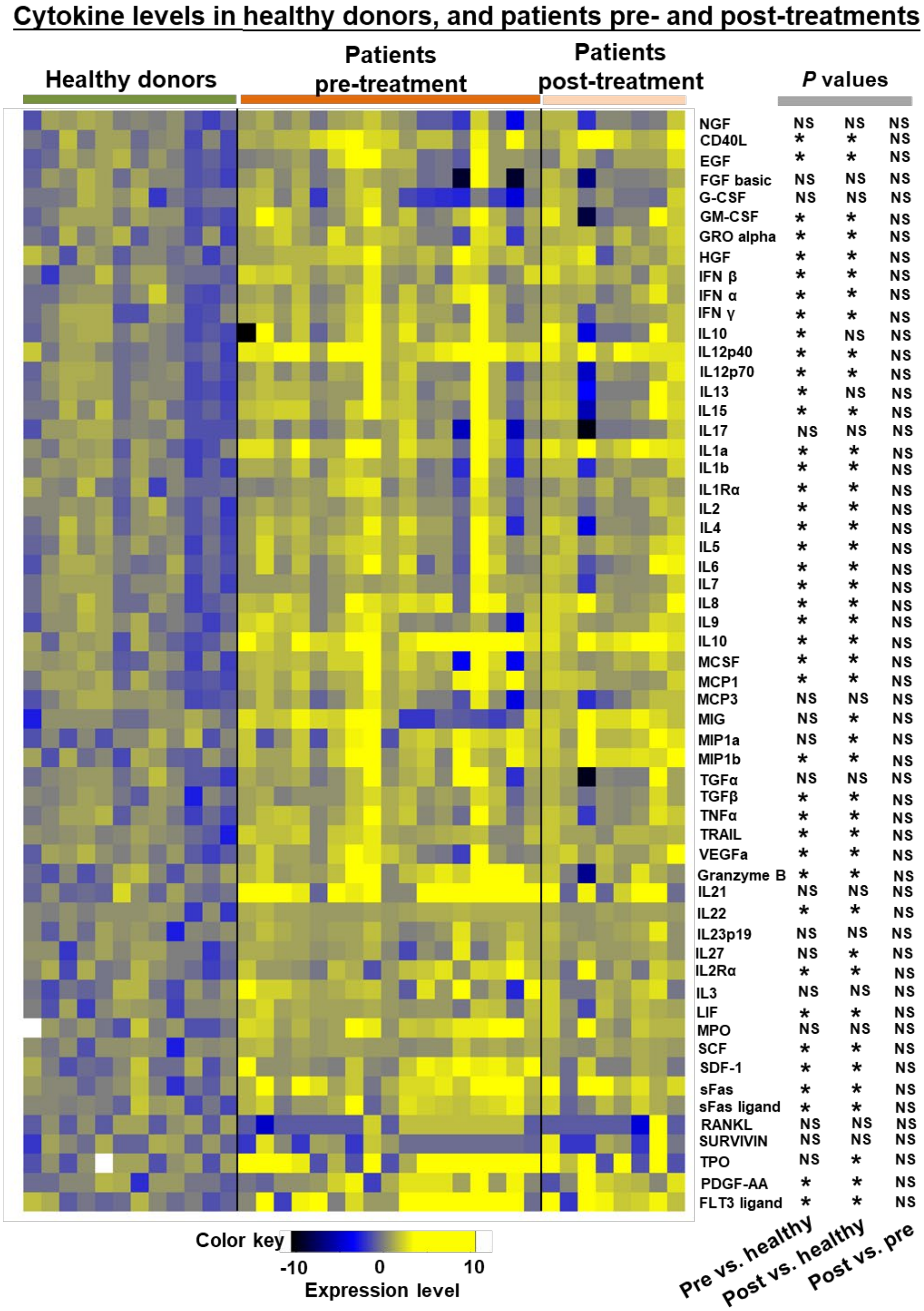

Supplementary Fig. 35 Cytokine levels in healthy donors and T-LGLL patients pre- and post-treatments. A heatmap shows cytokine levels in T-LGLL patients before and after treatments, and in healthy donors. These cytokines were measured in T-LGLL patients (n = 17) enrolled in our original clinical trial, included but not limited to the current cohort, with an independent group of healthy donors (n = 12). Three columns on the right indicate *P* values by comparing pre-treatment vs. healthy, post-treatment vs. healthy, and post-treatment vs. pre-treatment. A two-sided paired t-test between patients' samples before and after treatments (available paired samples, n = 8); a two-sided unpaired t-test between patients and healthy donors. **P* value < 0.05; NS, not significant.

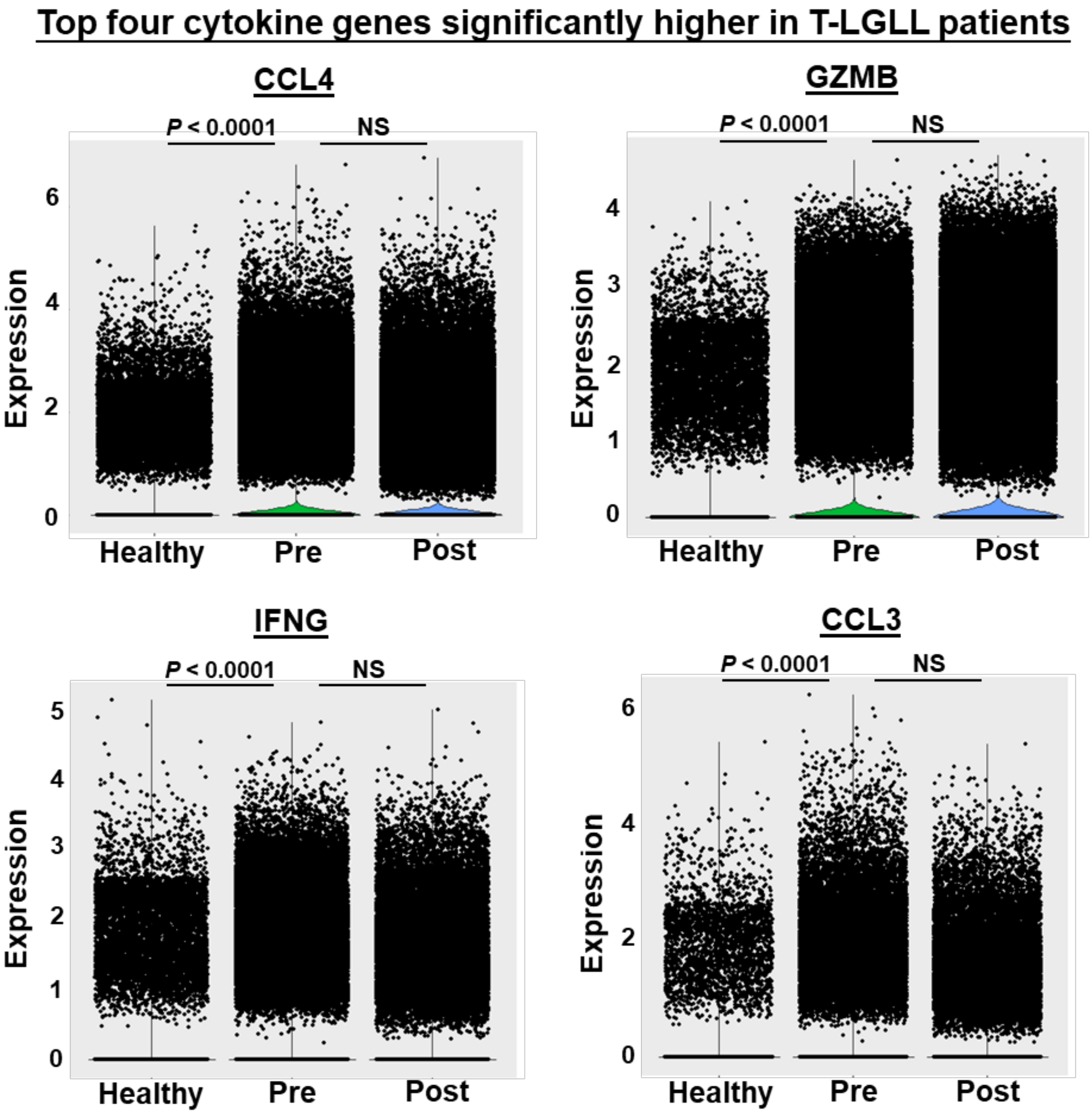

Supplementary Fig. 36 Top four cytokine genes significantly higher in T-LGLL patients. Expression levels of genes of these cytokines in the current single-cell sequencing study were evaluated (13 patients and seven healthy donors), and most of them were not detectable or were expressed only in small percentages of cells. Top four cytokine genes that were most significantly higher in T-LGLL patients were CCL4, GZMB, IFNG and CCL3. A two-sided paired t-test between patients' samples before and after treatments (n = 12); a two-sided unpaired t-test between patients (n = 13) and healthy donors (n = 7). *P* < 0.0001: as software generated *P* < 0.0001, due to a very small *P* value, an exact *P* value was unavailable; NS, not significant.

Supplementary References

1. Dumitriu, B. et al. Alemtuzumab in T-cell large granular lymphocytic leukaemia: interim results from a single-arm, open-label, phase 2 study. *Lancet Haematol.* **3**, e22-29 (2016).
2. Azizi, E. et al. Single-Cell Map of Diverse Immune Phenotypes in the Breast Tumor Microenvironment. *Cell* **174**, 1293-1308 (2018).
3. Redmond, D., Poran, A. & Elemento, O. Single-cell TCRseq: paired recovery of entire T-cell alpha and beta chain transcripts in T-cell receptors from single-cell RNAseq. *Genome Med.* **8**, 80 (2016).
4. Butler, A. et al. Integrating single-cell transcriptomic data across different conditions, technologies, and species. *Nat. Biotechnol.* **36**, 411-420 (2018).
5. Leek, J. T. et al. The sva package for removing batch effects and other unwanted variation in high-throughput experiments. *Bioinformatics* **28**, 882-883 (2012).
6. Zhang, F., Wu, Y. & Tian, W. A novel approach to remove the batch effect of single-cell data. *Cell Discov.* **5**, 46 (2019).
7. Tasaki, S. et al. Multi-omics monitoring of drug response in rheumatoid arthritis in pursuit of molecular remission. *Nat. Commun..* **9**, 2755 (2018).
8. Rosati, E. et al. Overview of methodologies for T-cell receptor repertoire analysis. *BMC Biotechnol.* **17**, 61 (2017).
9. Oakes, T. et al. Quantitative Characterization of the T Cell Receptor Repertoire of Naive and Memory Subsets Using an Integrated Experimental and Computational Pipeline Which Is Robust, Economical, and Versatile. *Front. Immunol.* **8**, 1267 (2017).
10. Desponds, J., Mora, T. & Walczak, A.M. Fluctuating fitness shapes the clone-size distribution of immune repertoires. *Proc. Natl. Acad. Sci. USA* **113**, 274-279 (2016).

11. Clemente, M. J. et al. Deep sequencing of the T-cell receptor repertoire in CD8+ T-large granular lymphocyte leukemia identifies signature landscapes. *Blood* **122**, 4077-4085 (2013).

12. Clemente, M. J. et al. Clonal drift demonstrates unexpected dynamics of the T-cell repertoire in T-large granular lymphocyte leukemia. *Blood* **118**, 4384-4393 (2011).

13 Chronister, D. et al. TCRMatch: Predicting T-Cell Receptor Specificity Based on Sequence Similarity to Previously Characterized Receptors. *Front. Immunol.* **12**, 673 (2021).

14 Ward, F. et al. The Immune Epitope Database and Analysis Resource in Epitope Discovery and Synthetic Vaccine Design. *Front. Immunol.* **8**, 278 (2017).

15. Glanville, J. et al. Identifying specificity groups in the T cell receptor repertoire. *Nature* **547**, 94-98 (2017).

16. P Angerer, et al. destiny: diffusion maps for large-scale single-cell data in R. *Bioinformatics* **32**, 1241-1243 (2016).

17. Alexa, A., Rahnenfuhrer, J. & Lengauer, T. Improved scoring of functional groups from gene expression data by decorrelating GO graph structure. *Bioinformatics* **22**, 1600-1607 (2006).

18. Shannon, P. et al. Cytoscape: a software environment for integrated models of biomolecular interaction networks. *Genome Res.* **13**, 2498-2504 (2003).

19. Cline, M. S. et al. Integration of biological networks and gene expression data using Cytoscape. *Nat. Protoc.* **2**, 2366-2382 (2007).

20. Shah, M. V. et al. Molecular profiling of LGL leukemia reveals role of sphingolipid signaling in survival of cytotoxic lymphocytes. *Blood* **112**, 770-781 (2008).

21. Lu, J. et al. Analysis of T-cell repertoire in hepatitis-associated aplastic anemia. *Blood* **103**, 4588-4593 (2004).

22. Alstott, J., Bullmore, E. & Plenz, D. Powerlaw: a Python package for analysis of heavy-tailed distributions. *PLOS One* **9**, e85777 (2014).

23. Qiu, Z.Y. et al. Large granular lymphocytosis after transplantation. *Oncotarget.* **8**, 81697-81708 (2017).

24. Lamy, T., Moignet, A. & Loughran, T.P. Jr. LGL leukemia: from pathogenesis to treatment. *Blood* **129**, 1082-1094 (2017).

25. Shah, M. V. et al. Molecular profiling of LGL leukemia reveals role of sphingolipid signaling in survival of cytotoxic lymphocytes. *Blood* **112**, 770-781 (2008)..

26. Brunner, T. et al. Expression of Fas ligand in activated T cells is regulated by c-Myc. *J. Biol. Chem.* **275**, 9767-9772 (2000).
