# Supplementary material for: Single-cell RNA sequencing coupled to TCR profiling of large granular lymphocyte leukemia T cells
Source: Nat Commun. 2022 Apr 11;13:1982. doi: 10.1038/s41467-022-29175-x (PMC9001664; doi:10.1038/s41467-022-29175-x)
Supplement: Supplementary file 2 — Description of Additional Supplementary Files [file 41467_2022_29175_MOESM2_ESM.docx]

**Description of Additional Supplementary Files**

Supplementary Data 1. Metrics summary of scRNA-seq and TCR profiling.

Supplementary Data 2. Overlap of TCR usage with a study in Blood Advances.

Supplementary Data 3. A clone specific gene list.

Supplementary Data 4. Clone specific GO terms.

Supplementary Data 5. Antigen sources of expanded TCRs

Supplementary Data 6. Gene set enrichment analysis results of differentially expressed genes in

T-LGLL patients.
