# Supplementary material for: Single-cell RNA sequencing coupled to TCR profiling of large granular lymphocyte leukemia T cells
Source: Nat Commun. 2022 Apr 11;13:1982. doi: 10.1038/s41467-022-29175-x (PMC9001664; doi:10.1038/s41467-022-29175-x)
Supplement: Supplementary file 9 — Reporting Summary [file 41467_2022_29175_MOESM9_ESM.pdf]

nature portfolio

Corresponding author(s): Shouguo Gao

Last updated by author(s): Dec 2, 2021

Reporting Summary

Nature Portfolio wishes to improve the reproducibility of the work that we publish. This form provides structure for consistency and transparency in reporting. For further information on Nature Portfolio policies, see our Editorial Policies and the Editorial Policy Checklist.

Statistics

For all statistical analyses, confirm that the following items are present in the figure legend, table legend, main text, or Methods section.

n/a	Confirmed	
☐	☒	The exact sample size (*n*) for each experimental group/condition, given as a discrete number and unit of measurement
☐	☒	A statement on whether measurements were taken from distinct samples or whether the same sample was measured repeatedly
☐	☒	The statistical test(s) used AND whether they are one- or two-sided<br>*Only common tests should be described solely by name; describe more complex techniques in the Methods section.*
☒	☐	A description of all covariates tested
☐	☒	A description of any assumptions or corrections, such as tests of normality and adjustment for multiple comparisons
☐	☒	A full description of the statistical parameters including central tendency (e.g. means) or other basic estimates (e.g. regression coefficient) AND variation (e.g. standard deviation) or associated estimates of uncertainty (e.g. confidence intervals)
☐	☒	For null hypothesis testing, the test statistic (e.g. *F*, *t*, *r*) with confidence intervals, effect sizes, degrees of freedom and *P* value noted<br>*Give P values as exact values whenever suitable.*
☒	☐	For Bayesian analysis, information on the choice of priors and Markov chain Monte Carlo settings
☒	☐	For hierarchical and complex designs, identification of the appropriate level for tests and full reporting of outcomes
☒	☐	Estimates of effect sizes (e.g. Cohen's *d*, Pearson's *r*), indicating how they were calculated

Our web collection on statistics for biologists contains articles on many of the points above.

Software and code

Policy information about availability of computer code

Data collection	Single-cell RNA sequencing (scRNA-seq) data were collected using an Illumina HiSeq 3000 PE150 sequencing. qRT-PCR data were collected by Qiagen (Fredrick, MD, USA), with Catalog number PAHS-039ZE-4. Flow cytometry data were collected using a Becton Dickinson Fortessa.
Data analysis	Flow cytometry data were analyzed by FlowJo (V10) software. scRNA-seq data were analyzed using the 10x CellRanger2.1.1 package, available from 10x Genomics (https://support.10xgenomics.com/single-cell-gene-expression/software/pipelines/latest/what-is-cell-ranger), the Seurat R package (http://satijalab.org/seurat/, v2.3.4), the destiny R package (version 3.5), the fgsea R package, the PhenoGraph Python package, the AUCell package in Bioconductor and the R package topGO v2.26 using the algorithm elim. TCR sequencing data were analyzed with TCRMatch (http://tools.iedb.org.tcrmatch), gliph (https://github.com/immunoengineer/gliph), WebLog (https://weblogo.berkeley.edu/logo.cgi), spoweRlaw and tCR R packages. The code is available at Github: https://github.com/shouguog/TLGL. The code is also provided in the Zenodo repository with the identifier (https://doi.org/10.5281/zenodo.5713623).

For manuscripts utilizing custom algorithms or software that are central to the research but not yet described in published literature, software must be made available to editors and reviewers. We strongly encourage code deposition in a community repository (e.g. GitHub). See the Nature Portfolio guidelines for submitting code & software for further information.

Data

Policy information about availability of data

All manuscripts must include a data availability statement. This statement should provide the following information, where applicable:

- Accession codes, unique identifiers, or web links for publicly available datasets
- A description of any restrictions on data availability
- For clinical datasets or third party data, please ensure that the statement adheres to our policy

Raw and analyzed sequencing data in this study can be found at the NCBI's Gene Expression Omnibus (series accession code GSE168859) and the Sequence Read Archive (accession code SRP310547). Third party TCR data were obtained from https://clients.adaptivebiotech.com/pub/kerr-2019-bloodadvances. Information of TCR antigens and epitopes was obtained from https://vdjdb.cdr3.net and https://www.iedb.org. Gene expression data for cell type identification were obtained at NCBI's Gene Expression Omnibus (GSE93777).

Field-specific reporting

Please select the one below that is the best fit for your research. If you are not sure, read the appropriate sections before making your selection.

☒ Life sciences ☐ Behavioural & social sciences ☐ Ecological, evolutionary & environmental sciences

For a reference copy of the document with all sections, see nature.com/documents/nr-reporting-summary-flat.pdf

Life sciences study design

All studies must disclose on these points even when the disclosure is negative.

Sample size	A sample size for the current experimental study was limited to an original clinical trial. No sample size calculation was performed: a sample size was determined arbitrarily based on availability of banked samples. Blood samples were obtained from 13 T-LGLL patients after written informed consent under the clinical protocol, 12 of whom had paired sample pre- and post-treatments. In total, ~500,000 single CD3+ T cells from 13 patients and seven healthy controls were sequenced.
Data exclusions	Sequencing data were processed and filtered using a well-established pipeline, and only single cells passing quality control were retained for further analyses.
Replication	Biological replication dose not apply to human samples, instead, we included multiple individuals in each group for comparison: 13 individuals (nine responders and four non-responders) in a patient group and seven individuals in a healthy control group.. No technical replication was performed, due to a cost of experiments.
Randomization	The original clinical protocol (NCT00345345) was designed as a non-randomized, off-label phase II study of alemtuzumab in subjects with T-LGLL.
Blinding	The original clinical protocol (NCT00345345) was designed as a non-randomized, off-label phase II study of alemtuzumab in subjects with T-LGLL. There is a single arm and blinding is not applicable.

Reporting for specific materials, systems and methods

We require information from authors about some types of materials, experimental systems and methods used in many studies. Here, indicate whether each material, system or method listed is relevant to your study. If you are not sure if a list item applies to your research, read the appropriate section before selecting a response.

Materials & experimental systems

n/a	Involved in the study	
☐	☒	Antibodies
☒	☐	Eukaryotic cell lines
☒	☐	Palaeontology and archaeology
☒	☐	Animals and other organisms
☐	☒	Human research participants
☐	☒	Clinical data
☒	☐	Dual use research of concern

Methods

n/a	Involved in the study	
☒	☐	ChIP-seq
☐	☒	Flow cytometry
☒	☐	MRI-based neuroimaging

Antibodies

Antibodies used	Antibodies used in this study were described in the Supplementary Methods, part of the manuscript.<br>1. CD3e Monoclonal Antibody (UCHT1), Qdot 605, ThermoFisher, Catalog # Q10054;

	2. V500 Mouse Anti-Human CD4, BD Biosciences, Catalog # 561488;<br>3. Human CD8 alpha Alexa Fluor® 750-conjugated Antibody, R&D systems, Catalog# FAB1509S-025;<br>4. IOTest Beta Mark TCR Vbeta Repertoire Kit, 25 Tests, RUO, Beckman Coulter, IM3497.
Validation	Flow cytometry results were not reported in the current manuscript, but only used to show consistency with our sequencing data. Flow cytometry results were reported in a previous publication of the original clinical trial, Lancet Haematol, 3(1): p. e22-9, 2016.<br>1. https://www.thermofisher.com/antibody/product/CD3e-Antibody-clone-UCHT1-Monoclonal/Q10054<br>2. https://www.bdbiosciences.com/en-us/products/reagents/flow-cytometry-reagents/research-reagents/single-color-antibodies-ruo/v500-mouse-anti-human-cd4.560768<br>3. https://www.rndsystems.com/products/human-cd8alpha-alexa-fluor-750-conjugated-antibody-37006_fab1509s<br>4. https://www.beckman.com/reagents/coulter-flow-cytometry/antibodies-and-kits/clinical-research-systems-and-kits/im3497

Human research participants

Policy information about studies involving human research participants

Population characteristics	There were 13 patients (71/F, 61/M, 29/F, 72/F, 77/M, 43/M, 51/F, 82/M, 51/M, 27/F, 66/F, 48/F and 39/M). They were all diagnosed as T-LGLL, and had prior treatments before enrollment into this study. Details were listed in Supplementary Table 1. Clinical characteristics of patients. There were seven age- and sex-matched healthy donors (39/F, 71/F, 55/F, 68/M, 41/F, 60/M and 41/M). Blood samples were obtained from these patients and healthy donors after written informed consent.
Recruitment	Patients' recruitment was reported in a previous publication of the clinical trial, Lancet Haematol, 3(1): p. e22-9, 2016. Consecutive subjects (ages 18–85) with T-LGLL were enrolled from October 1st 2006 to March 1st 2015 at the National Institutes of Health Clinical Center. Eligibility criteria included a history of cytopenias with circulating LGLs shown to be CD3 +CD8+ CD57+ T-LGLs by flow cytometry, with restricted or clonal TCR rearrangement by molecular studies. At least single-lineage cytopenia was required prior to enrollment: either absolute neutrophil count (ANC) < 500/μL; symptomatic anaemia with a haemoglobin < 9 g/dL or red cell transfusion requirement of > 2 units/month; or severe thrombocytopenia (< 20,000/μL) or moderate thrombocytopenia (< 50,000/μL) with active bleeding. Both treatment-naïve and treated T-LGLL patients were eligible for the clinical trial. Subjects with concomitant bone marrow myelodysplasia and cytogenetic abnormalities were eligible. Exclusion criteria included reactive LGLL lymphocytosis, a prior history of immunosuppressive therapy with alemtuzumab, infection not adequately responding to appropriate therapy, HIV seropositivity, pregnancy and history of carcinoma not considered cured. All patients had peripheral blood flow cytometry analysis with staining for standard, CLIA certified CD3, CD4, CD8 and CD57 antibodies performed in our clinical haematopathology laboratory at the Clinical Center. All patients prior to enrollment had to meet blood count criteria of cytopenia(s), and flow cytometric and molecular presence of T-LGLL clones.
Ethics oversight	Written informed consent was under the protocol (www.clinicaltrials.gov NCT00345345) approved by the Institutional Review Boards of National Heart, Lung, and Blood Institute, in accordance with the Declaration of Helsinki.

Note that full information on the approval of the study protocol must also be provided in the manuscript.

Clinical data

Policy information about clinical studies
All manuscripts should comply with the ICMJE guidelines for publication of clinical research and a completed CONSORT checklist must be included with all submissions.

Clinical trial registration	www.clinicaltrials.gov NCT00345345
Study protocol	www.clinicaltrials.gov NCT00345345
Data collection	Details were reported in a previous publication of the clinical trial, Lancet Haematol, 3(1): p. e22-9, 2016. The primary endpoint was haematologic response at three months after treatment.
Outcomes	Details were reported in acprevious publication of the clinical trial, Lancet Haematol, 3(1): p. e22-9, 2016. The primary endpoint was haematologic response at three months after treatment, and was assessed by measuring complete blood counts. A complete response (CR) was defined as normalization of all affected lineages, and a partial response (PR) was defined in neutropenic subjects as 100% increase in the ANC to > 500/μL, and in those with anaemia, any increase in haemoglobin of 2 g/dL or more observed in at least two serial measurements 1 week apart and sustained for one month or more without exogenous growth factors' support or transfusions. Transfusion-independence, haematologic response at six months, molecular response, relapse-free and overall survival were secondary endpoints, and were measured by collecting clinical data of transfusion amount, complete blood counts, TCRBV gene clonal analysis and patient survival. In subjects who relapsed after alemtuzumab, a second course of the same regimen was permitted per protocol. Landmark visits occurred at 3, 6 and 12 months and yearly thereafter.

Flow Cytometry

Plots

Confirm that:

☒ The axis labels state the marker and fluorochrome used (e.g. CD4-FITC).

☒ The axis scales are clearly visible. Include numbers along axes only for bottom left plot of group (a 'group' is an analysis of identical markers).

☒ All plots are contour plots with outliers or pseudocolor plots.

☒ A numerical value for number of cells or percentage (with statistics) is provided.

Methodology

Sample preparation	Flow cytometry results were not reported in the current manuscript, but only used to show consistency with our sequencing data.
Instrument	A Becton Dickinson Fortessa
Software	Data were analyzed using FlowJo software (Tree Star Inc.)
Cell population abundance	Isolated PBMCs from patients and healthy donors were cryopreserved in liquid nitrogen according to standard protocols until use. T cells were enriched with the EasySep Human T cell Isolation kit (Stemcell Technologies). Purity of enriched T cells were > 90% determined by flow cytometry.
Gating strategy	Cells were first plotted on a FSC (x-axis) and SSC (y-axis) plot and gated for single cells. And then CD3 expression levels was used to determined CD3+ T cells and others.

☒ Tick this box to confirm that a figure exemplifying the gating strategy is provided in the Supplementary Information.
